# Searching and designing potential inhibitors for SARS-CoV-2 Mpro from natural sources using atomistic and deep-learning calculations†

**DOI:** 10.1039/d1ra06534c

**Published:** 2021-11-29

**Authors:** Nguyen Minh Tam, Duc-Hung Pham, Dinh Minh Hiep, Phuong-Thao Tran, Duong Tuan Quang, Son Tung Ngo

**Affiliations:** Computational Chemistry Research Group, Ton Duc Thang University Ho Chi Minh City Vietnam; Faculty of Applied Sciences, Ton Duc Thang University Ho Chi Minh City Vietnam; Division of Immunobiology, Cincinnati Children's Hospital Medical Center Cincinnati Ohio 45229 USA; Department of Agriculture and Rural Development Ho Chi Minh City 71007 Vietnam; Hanoi University of Pharmacy Hanoi 11021 Vietnam; Department of Chemistry, Hue University, Thua Thien Hue Province Hue City Vietnam; Laboratory of Theoretical and Computational Biophysics, Ton Duc Thang University Ho Chi Minh City Vietnam ngosontung@tdtu.edu.vn

## Abstract

The spread of severe acute respiratory syndrome coronavirus 2 novel coronavirus (SARS-CoV-2) worldwide has caused the coronavirus disease 2019 (COVID-19) pandemic. A hundred million people were infected, resulting in several millions of death worldwide. In order to prevent viral replication, scientists have been aiming to prevent the biological activity of the SARS-CoV-2 main protease (3CL pro or Mpro). In this work, we demonstrate that using a reasonable combination of deep-learning calculations and atomistic simulations could lead to a new approach for developing SARS-CoV-2 main protease (Mpro) inhibitors. Initially, the binding affinities of the natural compounds to SARS-CoV-2 Mpro were estimated *via* atomistic simulations. The compound tomatine, thevetine, and tribuloside could bind to SARS-CoV-2 Mpro with nanomolar/high-nanomolar affinities. Secondly, the deep-learning (DL) calculations were performed to chemically alter the top-lead natural compounds to improve ligand-binding affinity. The obtained results were then validated by free energy calculations using atomistic simulations. The outcome of the research will probably boost COVID-19 therapy.

## Abstract 2

The hybrid DeepFrag/atomistic simulation approach could lead to a new scheme for developing SARS-CoV-2 3CLpro/Mpro inhibitors.

## Graphical abstract

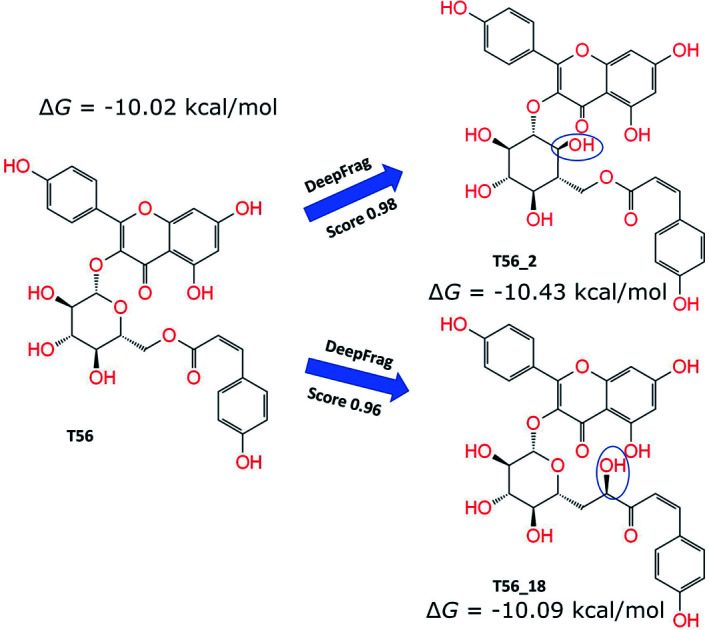

## Introduction

SARS-CoV-2, which belongs to the β-coronavirus genus, shares 79.6% of sequence identity with SARS-CoV.^1^ This virus is supposed to have originated from bats, but other animals, such as pangolins, are also possible intermediate hosts. SARS-CoV-2 has been causing the coronavirus disease 2019 (COVID-19) pandemic,^2^ which has affected more than 182 million patients and is associated with about 4 million deaths worldwide as of July 2021. SARS-CoV-2, a single positive-strand RNA virus with spherical morphology is composed of four main structural proteins, including spike, envelope, membrane and nucleocapsid proteins that are crucial for the synthesis of viral proteins and viral replication.^3^ The spike (S) protein of SARS-CoV-2 is present on the viral surface as a homo-trimer, which is researched thoroughly because this is the part that the virus employs in order to enter human cells by binding to angiotensin-converting enzyme 2 receptor (ACE2).^4^ This receptor is present in different organs in the human body, such as the lung, heart, and liver.^4^

The health burden of coronavirus is increasing significantly with the emergence of new variants that can decrease the effectiveness of vaccines and the complication of co-infection of human patients with other viruses, bacteria, and fungi.^5^ These present a challenge to develop new drugs that can effectively cure or at least reduce the severity of COVID-19. Many drugs have been tested in pre-clinical and clinical trials so far, including remdesivir, hydroxychloroquine, lopinavir/ritonavir, interferon β-1a, tocilizumab, favipiravir, plitidepsin, convalescent plasma infusions, and monoclonal antibodies, among many others, for their effect on SARS-CoV-2 elimination.^6–8^ Especially, numerous studies were carried out to find a promising inhibitor to prevent the SARS-CoV-2 Mpro since it associates with the cleavage of polyproteins to polypeptides accounting for the viral functionalities and replication.^9–17^ However, none of them are really curative for the disease.

Characterizing the binding free energy (Δ*G*) between proteins and ligands is a critical issue in predicting potential inhibitors for inhibiting biological targets.^18–23^ The metric is popularly estimated using computational approaches.^24^ Rigorous calculations usually provide correlated results with the respective experiments.^25^ Required costs and time for therapeutic development are thus reduced.^20,26^ In particular, molecular docking simulations are often used to initially estimate the ligand binding pose and free energy to enzyme targets.^27,28^ Docking simulations can rapidly provide results with appropriate correlation coefficients.^29^ However, molecular docking uses several constraints to accelerate the calculation speed, the obtained results are normally required to refine *via* more accurate approaches. Molecular dynamics simulations are then employed to unravel the outcome of docking calculations.^11,30^ Moreover, in recent years, the development of deep-learning (DL) approaches has brought many benefits for various areas of society. DL has also been employed in CADD^31^ because it is able to learn the mapping from molecular inputs such as structural, physical, and chemical properties to ligand binding affinities and poses. In particular, a deep convolutional neural network can be used to alter the chemical structure of ligands to improve ligand-binding free energy.^32,33^ DL models are also employed to characterize the binding affinity of ligands.^34–36^

In addition, natural compounds historically contribute to pharmacotherapy, especially for infected diseases.^37,38^ Numerous studies have indicated that natural products can prevent SARS-CoV-2,^39,40^ especially SARS-CoV-2 Mpro.^11,41^ Therefore, in this work, we have screened natural compounds for preventing SARS-CoV-2 Mpro using rigorously computational approaches. As well, the top-lead compounds were chemically modified to improve the binding affinity *via* DeepFrag, a deep learning (DL) model.^32^ The binding affinity of these ligands was then validated using atomistic simulations. The calculated improvement was repeated until the ligand-binding affinity was not enhanced. Totally, there are 17, 27, and 34 compounds exhibiting nanomolar, high-nanomolar, and sub-micromolar affinities to SARS-CoV-2 Mpro, respectively. Using a reasonable combination of DL calculations and atomistic simulations could lead to a new approach for developing SARS-CoV-2 Mpro inhibitors.

## Materials and methods

### Structure of SARS-CoV-2 Mpro and ligands

The three-dimensional shape of SARS-CoV-2 Mpro was downloaded from the Protein Data Bank (PDB ID: 7JYC).^42^ The protein structure was obtained *via* X-ray diffraction with a resolution of 1.79 Å. The structure of ligands was downloaded from the PubChem database.^43^ The PubChem identity and two-dimensional structure of ligands are mentioned in the ESI.†^43^ In particular, 41 compounds, denoted from K1 to K41, were found from *Cordyceps*.^44^ 339 compounds, denoted from T1 to T339, are natural compounds reported in the previous study.^45^ 17 natural compounds, denoted from w1 to w17, were tested for binding affinity to SARS-CoV.^46^ Moreover, 60 compounds were generated over DL calculations, whose structures were also reported in the ESI† file.

### Molecular docking simulations

**Fig. 1:**
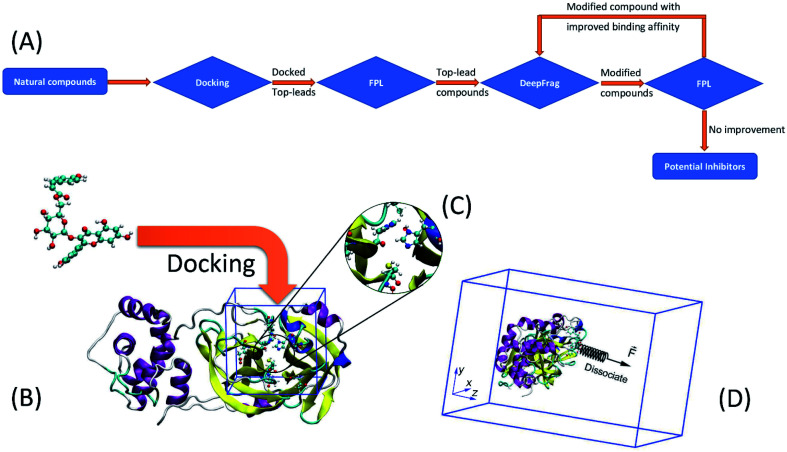
(A) Computational scheme was applied to characterize and design potential inhibitors for SARS-CoV-2 Mpro using atomistic simulations and machine learning calculations. (B) A ligand was docked to SARS-CoV-2 Mpro using AutoDock Vina. (C) The protonation states of the catalytic dyad His41 and Cys145. (D) A ligand was dissociated from the bound state using external-harmonic force 
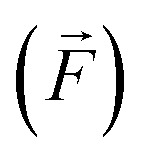
 during FPL simulations. 
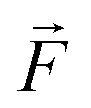
 was put on the ligand center of mass in order to force the ligand to mobilize out of the protease binding cavity.

The ligand-binding pose and affinity were initially assessed *via* AutoDock Vina (Fig. 1A),^47^ which is an appropriate package to perform this task.^25^ In particular, the ligands and receptors were prepared for docking simulations *via* AutoDockTools 1.5.6.^48^ The docking global search parameter exhaustiveness is selected as the default value. The ligand-binding pose was searched in the space of the docking grid, whereas the grid center is the narlaprevir center of mass and the grid size is 2.40 × 2.40 × 2.40 nm. It should be noted that narlaprevir is the native ligand of 7JYC.^42^ Only the best docking mode was recorded for further calculations.

### Fast pulling of ligand (FPL) simulations

GROMACS 5.1.5 (ref. 49) was used to simulate the dissociation process of ligands out of SARS-CoV-2 Mpro binding cavity. In particular, the protease and ions were topologized using the Amber99SB-iLDN force field.^50^ Due to the importance of the catalytic dyad in the biological activity of the protease,^51^ the protonation state of His41 and Cys145 was assigned as described in Fig. 1B. Besides, protonation states of other residues were assigned by GROMACS *via* canonical p*K*_a_ metrics according to the previous work.^25^ A water molecule was parameterized *via* the TIP3P water model.^52^ Moreover, a ligand was represented using the general Amber force field (GAFF)^53^ produced by ACPYPE and AmberTools18 packages.^54,55^ In particular, the geometrical parameters and atomic charges of a ligand were provided from the quantum mechanics calculations using the B3LYP functional with 6-31G(d,p) basis set. During which, ligand atomic charges were fitted by the restrained electrostatic potential (RESP) scheme.^56^ It should be noted that quantum calculations were carried out using the implicit solvent option, *ε* = 78.4.

The complex was inserted into a rectangular periodic boundary condition box as described in Fig. 1C. The box size (*x*, *y*, *z*) is (9.83, 5.92, 8.70) in the unit of nm. The solvated complex thus consists of *ca.* 50 000 atoms, which include a protease, a ligand, water molecules, and Na^+^ ions. Energy minimization simulations were initially carried out to optimize the solvated complex. The system was then relaxed over 0.1 ns of *NVT* and 2.0 ns of *NPT* simulations. The relaxed conformation was employed as the starting shape for steered-molecular dynamics (SMD) simulations. The simulations were performed using parameters referred to in the previous work.^11^ During simulations, the integral was calculated every 2 fs. The simulation temperature was 310 K and the pressure of *NPT* simulation was chosen as 1 atm. A non-bonded pair was available when the distance between two atoms was smaller than 0.9 nm. The fast particle-mesh Ewald electrostatics scheme^57^ was utilized to calculate electrostatic interactions, besides the cutoff scheme was employed to treat van der Waals (vdW) interaction. Each calculation was independently repeated 8 times to guarantee sampling.

During SMD simulations, the ligand was dissociated *via* an external harmonic force, which has a cantilever spring constant *ν* = 600 kJ mol^−1^ nm^−2^ and pulling velocity *k* = 0.005 nm ps^−1^.^58^ The recorded pulling work, 
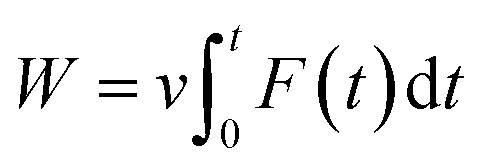
, is associated with the binding free energy, Δ*G*, *via* isobaric-isothermal Jarzynski equality,^59^
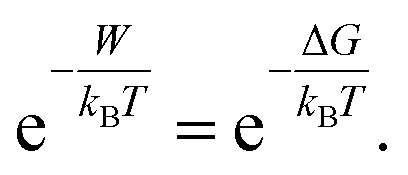


### Deep-learning calculations

DeepFrag,^32,33^ a deep convolutional neural network, was used to predict the chemical modification of the ligand to enhance the binding affinity. In particular, the complex structure of SARS-CoV-2 Mpro and top-lead compounds revealed by FPL simulations were used as the initial conformation of DL calculations. In particular, the PDB files of ligands and protease were uploaded to DeepFrag web application (https://durrantlab.pitt.edu/deepfrag/). The ligand atoms were then selected to check if they could be replaced by another chemical group. The possible alteration was recorded if the DeepFrag score was larger than 0.90.

### Analyzed tools

Before MD simulations, the ligand protonation state was predicted using the chemicalize webserver.^60^ Ligand interaction diagram was generated by the Maestro free package,^61^ in which the hydrogen bond (HB) and side-chain (SC) contacts were predicted using the default option of the Maestro package. In addition, human intestinal absorption (HIA), logP, and toxicity of the compounds were estimated using the PreADMET webserver.^62^

## Results and discussion

### Natural compounds bind to SARS-CoV-2 Mpro

**Fig. 2:**
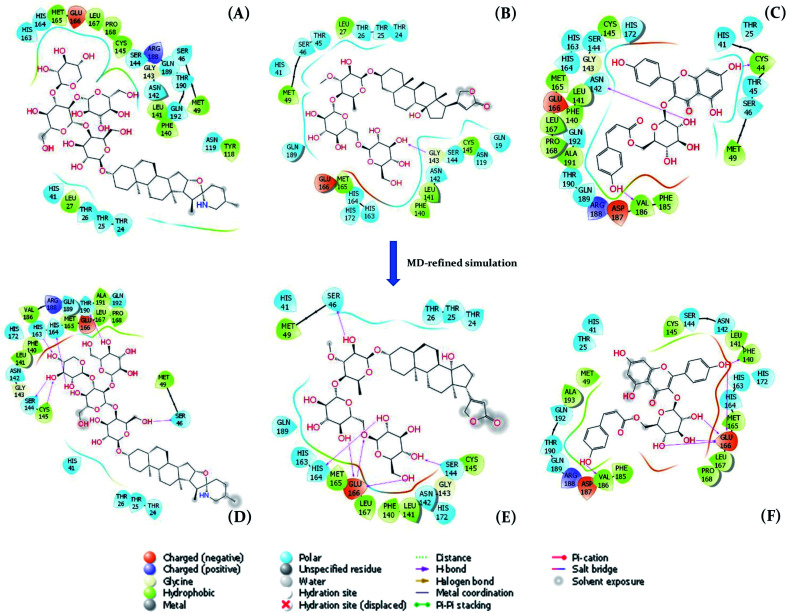
The two-dimensional interaction diagrams between SARS-CoV-2 Mpro and their ligands. (A), (B), and (C) are T82, T17, and T56 binds to SARS-CoV-2 Mpro obtained by AutoDock Vina, respectively. (D), (E), and (F) are T82, T17, and T56 binds to SARS-CoV-2 Mpro obtained by MD-refined simulations, in which the described structure is the clustered shape over the last snapshots of the relaxation simulations.

Molecular docking simulations are normally used to rapidly assess ligand-binding pose and affinity to enzyme targets.^63^ AutoDock Vina,^47^ a free package, was usually used to dock the inhibitor to SARS-CoV-2 Mpro^64,65^ since its results formed appropriate correlation coefficients between docking results and experiments, *R*_Vina_ ranging from 0.60 ± 0.13 to 0.82 ± 0.08,^25,58,66^ and success rates, ρ̂_Vina_ = 67%.^25^ Therefore, in this work, AutoDock Vina^47^ was utilized to find a shortlist of compounds having large docking energy to SARS-CoV-2 Mpro. The docking results are fully described in Table S1 of the ESI† file. Docking energy ranged from −3.1 to −8.9 kcal mol^−1^ with an average value of −6.14 ± 0.06 kcal mol^−1^. In particular, 40 compounds, occupying 10% of total substrates were then re-assessed for the ligand-binding affinity *via* molecular dynamics simulations. The interaction diagrams of these compounds in SARS-CoV-2 Mpro were generated by the Maestro package^61^ and displaced in Fig. 2 and Table S2 of the ESI† file. On average, these ligands adopted 1.2 ± 0.2 HB to the protease, in which ligands favorably contact with the residue Thr26, Cys44, Ser46, Leu141, Asn142, Gly143, and Glu166. Besides, interestingly, T34 and T180 compounds can directly disturb the catalytic dyad since forming HB to Cys145. Moreover, the docking energy of these ligands falls in the range from −7.6 to −8.9 kcal mol^−1^ with a mean of −7.93 ± 0.05 kcal mol^−1^. The obtained affinities are larger than that of the other inhibitors reported in previous studies using AutoDock Vina such as α-ketoamide inhibitors 11n (−6.4 kcal mol^−1^), 11r (−6.9 kcal mol^−1^), and 11s (−7.0 kcal mol^−1^).^67^ Azo imidazole derivatives was also docked to SARS-CoV-2 Mpro *via* AutoDock Vina, in which the docked energies ranged from −6.7 to −8.1 kcal mol^−1^.^68^ Consequently, it is better than the docking energy of 26 inhibitors of SARS-CoV-2 Mpro (ranging from −5.1 to −7.2 kcal mol^−1^) mentioned in the recent work.^65^ However, the obtained affinities were smaller than the top-lead compounds of Natural Product Arlats, which the docking energies adopted in the range from −8.2 to −9.4 kcal mol^−1^.^69^

### Unbinding ligand to refine binding affinity

AutoDock Vina uses numerous approximations such as acquired united-atom model, rigid receptor, and rarely tested ligand positions, the obtained results are thus required to refine *via* MD simulations.^23,58,71^ In this work, FPL simulations were employed to refine the docking outcome,^58^ because the approach formed a good correlation coefficient to the respective experiments with a value, *R*_FPL_, ranging from −0.74 ± 0.11 to −0.76 ± 0.01.^25,58^ It should be noted that the correlation coefficient is a negative mean that required larger pulling work corresponding to the smaller binding free energy. Besides, with the correlation coefficient, the FPL scheme is only behind the free energy perturbation method,^72^ which is known as the most accurate method and required huge computing resources, in ranking ligand-binding affinity.^25^ In the FPL scheme, the system was relaxed to reach equilibrium states before the ligand was forced to dissociate with the protease *via* an external force. During relaxation simulations, the ligand-binding pose was cleared (*cf.*Fig. 2 and Table S2 of the ESI† file). Interestingly, the number of HBs between ligands and the protease was increased over MD-refined simulations, in which the counted contact is 1.9 ± 0.3. The residue Thr24, Thr26, Cys44, Ser46, Asn142, Gly143, Ser144, and Glu166 popularly adopted HB contact to ligands. The change of the important residue list implies the incorrect part of molecular docking simulations.

The ligand would be then forced to mobilize from bound to unbound states. The recorded work of pulling force *W* would be used as a critical term to estimate the ligand-binding free energy according to the formula Δ*G*^Pre^_FPL_ = −0.056 × *W* − 5.512 reported in previous work.^58^ The larger work *W* means the stronger ligand binder. In order to predict the binding free energy of 40 ligands, 320 independent FPL calculations were carried out. The obtained results are reported in Table 1. The recorded pulling forces along the dissociated pathways are mentioned in Table S3 of the ESI† file. The mean rupture force *F*_Max_, which is the maximum pulling force, is also mentioned in Table 1 since it could be used as a metric to rank ligand-binding affinity.^73^ The *F*_Max_values were measured in a range from 376.8 ± 29.2 to 721.5 + 38.2 pN. Besides, the average of pulling works dropped in the range from 29.1 ± 2.6 to 108.6 ± 5.7 kcal mol^−1^ corresponds to the predicted binding free energy Δ*G*^Pre^_FPL_ ranging from −7.14 to −11.59 kcal mol^−1^, respectively. The predicted value of the half-maximal inhibitory concentration IC^Pre^_50_ was thus computed *via* the formula , where *R* is the gas constant and *T* is the absolute temperature. The IC^Pre^_50_ of ligands falls in the range from micromolar to nanomolar affinity (*cf.*Table 1), in which three compounds T82, T17, and T56 adopted a strong binding to SARS-CoV-2 Mpro. The obtained results are well consistent with the HB analyses, in which T82, T17, and T56 formed 6, 5, and 6 HBs to the protease. Consequently, there are 25, 19, and 23 residues that formed SC contacts to T82, T17, and T56, respectively. Therefore, three compounds probably play as highly potent inhibitors for SARS-CoV-2 Mpro. Moreover, 14 compounds adopting sub-nanomolar affinity (Table 1) could efficiently prevent SARS-CoV-2 Mpro. Especially, the binding affinity of 17 top-lead compounds is significantly larger than that of EGCG, which formed a binding affinity of Δ*G*^Pre^_FPL_ = −7.86 kcal mol^−1^ in FPL calculations. Besides, it should be noted that the compound formed an IC_50_ value of 0.874 μM *versus* SARS-CoV-2 Mpro.^70^ The Δ*G*_EXP_ of EGCG was thus calculated as −8.30 kcal mol^−1^ in an assumption that the IC_50_ is equal to *k*_i_. Furthermore, it should be noted that T82, tomatine, is a glycoalkaloid extracted from the tomato plant. Tomatine is popularly used as a plant fungicide and as a precipitating agent for cholesterol.^74^T17, thevetine, is cardiac glycosides obtained from yellow oleander (*Thevetia peruviana*) seeds.^75^ T56, tribuloside, is a flavonoid that can be isolated from *Tribulus terrestris* L.^76^

**Table 1:** The calculated results of 41 compounds to SARS-CoV-2 Mpro using molecular docking and FPL simulations

No.	Code	PubChem ID	Name	Δ*G*_Dock_	*F*_Max_	*W*	Δ*G*^Pre^_FPL_^a^	IC^Pre^_50_ range^b^	Δ*G*_EXP_^c^
1	T82	28523	Tomatine	−8.9	721.5 ± 38.2	108.6 ± 5.7	−11.59	Nanomolar	
2	T17	159331	Thevetine	−7.7	635.7 ± 34.6	86.3 ± 2.3	−10.35	High-nanomolar	
3	T56	10175330	Tribuloside	−7.9	701.2 ± 45.1	80.5 ± 4.2	−10.02	High-nanomolar	
4	T117	5282160	Quercimeritrin	−7.7	634.7 ± 35.0	75.4 ± 4.1	−9.73	Sub-micromolar	
5	T25	31310	Scillaren	−8.3	599.2 ± 44.4	72.1 ± 3.9	−9.55	Sub-micromolar	
6	T61	73568	Corilagin	−8.1	700.2 ± 40.5	72.2 ± 3.8	−9.55	Sub-micromolar	
7	T44	6325292	Gomphrenin III	−7.6	597.7 ± 27.5	65.6 ± 1.7	−9.19	Sub-micromolar	
8	T26	222154	Proscillaridin	−8.2	578.7 ± 28.1	63.0 ± 4.3	−9.04	Sub-micromolar	
9	T33	185586	Melianotriol	−7.7	686.1 ± 36.9	61.8 ± 4.1	−8.98	Sub-micromolar	
10	T52	441840	Adynerin	−8.1	542.3 ± 24.9	61.1 ± 2.3	−8.93	Sub-micromolar	
11	T24	5317157	Equisetrin	−7.9	557.0 ± 29.2	59.1 ± 4.7	−8.82	Sub-micromolar	
12	T3	5281627	Hinokiflavone	−8.6	574.4 ± 41.0	57.7 ± 3.8	−8.74	Sub-micromolar	
13	T202	441295	Ginkgolide C	−7.9	639.4 ± 23.8	55.3 ± 2.4	−8.61	Sub-micromolar	
14	T55	5316647	Cynarine	−7.7	488.7 ± 33.5	55.3 ± 6.1	−8.61	Sub-micromolar	
15	T126	5280805	Rutin	−7.6	539.7 ± 39.7	55.2 ± 4.4	−8.60	Sub-micromolar	
16	T34	185617	Scutellarin	−7.7	543.9 ± 34.8	55.0 ± 4.7	−8.59	Sub-micromolar	
17	T19	10028469	Melianodiol	−7.8	563.8 ± 23.4	54.8 ± 2.9	−8.58	Sub-micromolar	
18	T13	5281600	Amentoflavone	−8.6	508.0 ± 35.7	53.4 ± 3.0	−8.50	Micromolar	
19	T121	32024	Alpha-antiarin	−7.9	558.6 ± 28.0	53.2 ± 3.4	−8.49	Micromolar	
20	T27	11013	Rhodexin A	−7.8	509.3 ± 37.7	51.6 ± 3.3	−8.40	Micromolar	
21	T115	15515703	Jujubogenin	−7.7	603.8 ± 24.0	51.3 ± 2.5	−8.39	Micromolar	
22	T182	3032482	Ecdysterone	−7.7	544.1 ± 37.3	50.3 ± 4.0	−8.33	Micromolar	
23	T14	65071	Limonin	−8.9	540.0 ± 13.0	49.9 ± 1.5	−8.31	Micromolar	
24	W22	3000706	Valinomycin	−7.6	493.2 ± 35.4	47.0 ± 3.1	−8.14	Micromolar	
25	T179	73432	Brusatol	−7.7	483.7 ± 34.3	43.6 ± 3.4	−7.95	Micromolar	
26	T58	10494	Oleanolic acid	−7.6	495.0 ± 39.1	42.2 ± 1.7	−7.87	Micromolar	
27	T65	131900	Peimine	−8.1	460.9 ± 29.4	42.0 ± 2.1	−7.86	Micromolar	
28	T35	3083631	Chlorogenin	−7.8	486.4 ± 42.1	42.0 ± 3.5	−7.86	Micromolar	
29	T119	65064	(−)-Epigallocatechin 3-gallate (EGCG)	−7.5	517.5 ± 24.1	41.9 ± 3.4	−7.86	Micromolar	−8.30
30	T23	72307	Sesamin	−7.7	514.2 ± 34.5	39.8 ± 3.0	−7.74	Micromolar	
31	T107	4970	Protopine	−8.1	546.0 ± 35.2	37.5 ± 2.4	−7.61	Micromolar	
32	T20	167691	Peiminine	−8.1	441.9 ± 34.8	36.9 ± 4.2	−7.58	Micromolar	
33	T7	5270604	Taraxasterol	−7.7	461.9 ± 32.3	36.9 ± 2.3	−7.58	Micromolar	
34	T50	119041	Obacunone	−7.8	440.2 ± 19.1	35.9 ± 1.9	−7.52	Micromolar	
35	T180	98570	Allocryptopine	−8.4	432.8 ± 22.7	34.6 ± 1.8	−7.45	Micromolar	
36	T30	470259	Arnidiol	−7.6	407.9 ± 31.5	34.4 ± 1.4	−7.44	Micromolar	
37	T4	15560423	Kulactone	−7.6	434.8 ± 16.9	34.3 ± 2.6	−7.43	Micromolar	
38	T8	91453	Hecogenin	−7.7	422.4 ± 26.1	33.9 ± 2.8	−7.41	Micromolar	
39	T102	442814	Pachyrrhizone	−7.7	449.3 ± 28.9	32.9 ± 2.8	−7.35	Micromolar	
40	T1	31342	Salasodine	−7.7	376.8 ± 29.2	31.5 ± 2.7	−7.28	Micromolar	
41	T11	5154	Sanguinarine	−8.2	424.6 ± 30.5	29.1 ± 2.6	−7.14	Micromolar	

a The predicted binding free energy Δ*G*^Pre^_FPL_ = −0.056 × *W* − 5.512 kcal mol^−1^.^58^

b The predicted IC^Pre^_50_ was calculated *via* formula 
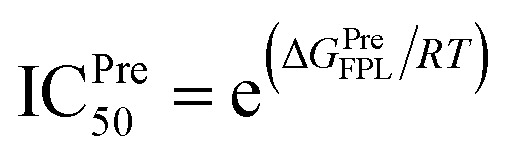
 using hypothesis that IC_50_ equals inhibition constant *k*_i_.

c The experimental affinity Δ*G*_EXP_ was approximately calculated *via* the IC_50_ value (ref. 70) with an assumption that the IC_50_ equal to *k*_i_ (inhibition constant). The calculated error is the standard error of the average (SE). The unit of force and energy in pN and kcal mol^−1^, respectively.

### Design of stronger binding ligand *via* DL + FPL calculations

**Fig. 3:**
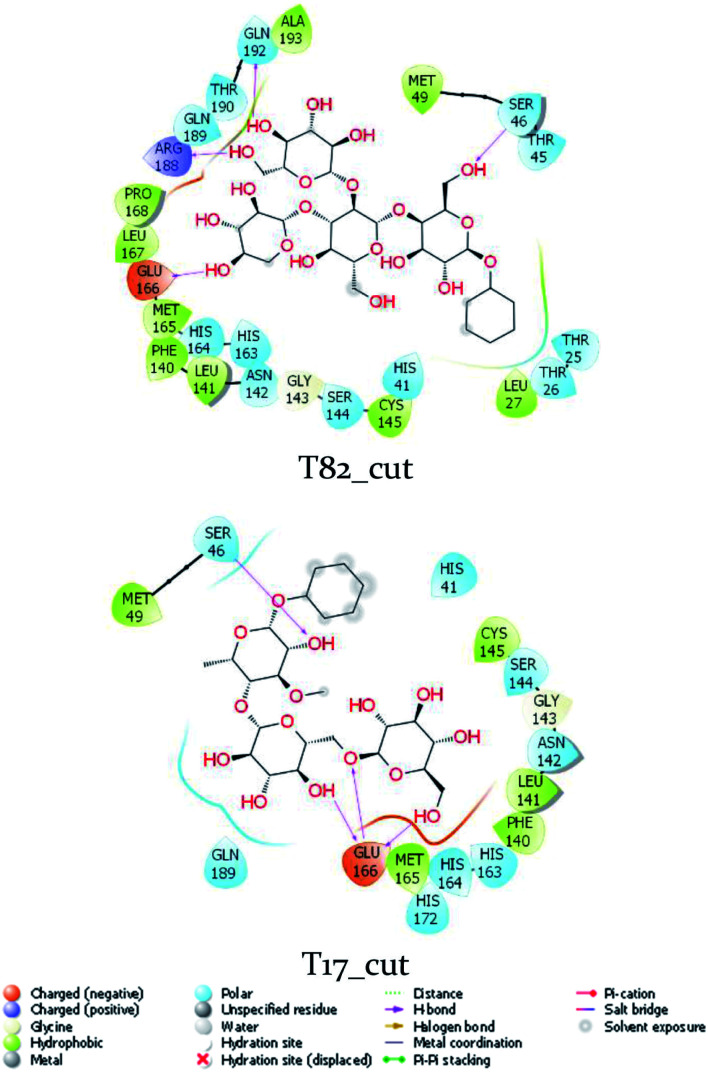
The interaction diagram between truncated T82 and T17 with SARS-CoV-2 Mpro. The diagram was analyzed from MD-refined structures by Maestro free package.

Although the compound T82 formed the strongest binding affinity to SARS-CoV-2 Mpro, the molecule is too big and the steroid group is located outside the binding cavity and fully exposed to the solvent (Fig. 2). Besides, the rest of the molecule fully fitted in the protease binding cavity. The steroid group was thus proposed to be removed from the molecule, resulting in the compound T82_cut fully fitting the binding cavity (Fig. 3). FPL calculations were then performed to predict the ligand-binding affinity. The calculated metrics including *F*_Max_ and *W* were found to be 748.3 ± 48.4 pN and 96.3 ± 5.2 kcal mol^−1^, respectively. The binding free energy was predicted to be −10.90 kcal mol^−1^. Although the binding affinity of T82_cut is smaller than that of T82, the term is larger than that of T17 and T56. Moreover, we also proposed to remove the triterpenoids saponin group from the compound T17 since the group is located outside the binding cavity and fully exposed to the solvent (Fig. 2). FPL calculations indicated that the predicted binding free energy between T17_cut and SARS-CoV-2 Mpro of −9.47 kcal mol^−1^ (Fig. 3). Therefore, in the next step, a deep convolutional neural network, DeepFrag,^32^ was employed to chemically modify the three compounds T82_cut, T17_cut, and T56 with the expectation that the altered compounds will form a stronger binding affinity to the protease.

**Fig. 4:**
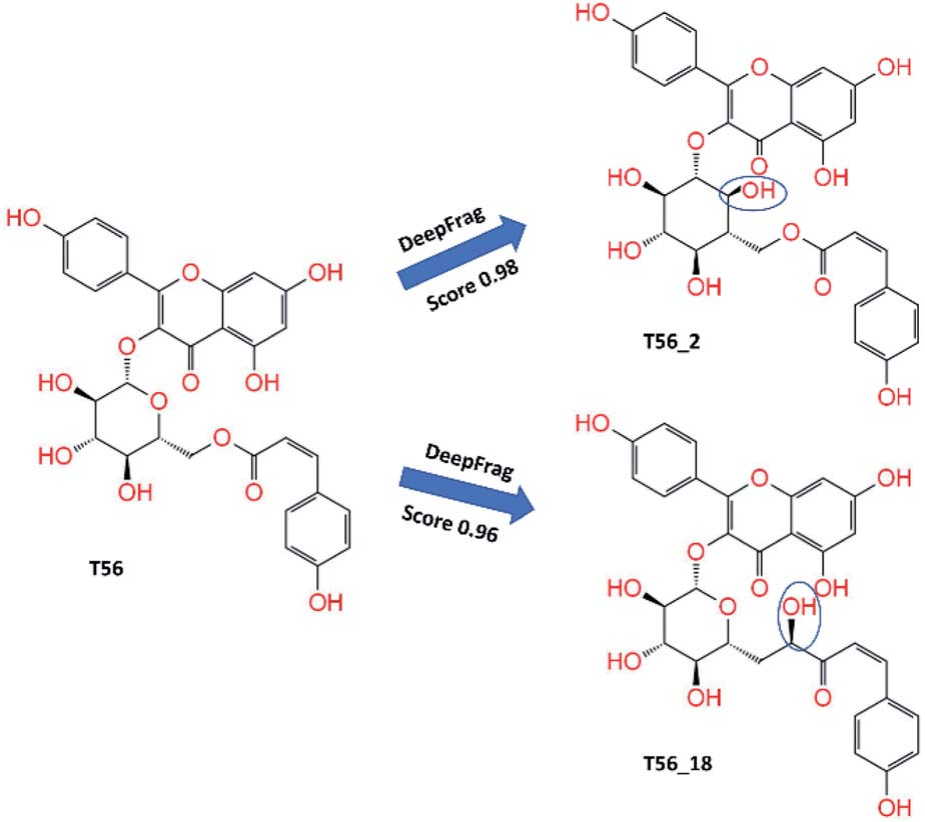
Critical compounds were predicted by DeepFrag calculations. Group atoms, which are noted with the blue curve, are the modified positions.

Total 60 modified compounds were proposed *via* DeepFrag package that probably forms a larger binding affinity to SARS-CoV-2 Mpro. Initially, the compound name was denoted with a type of T82_*x*, T17_*x*, and T56_*x*, in which *x* is the index of the replaced atom (Fig. 4 and S1 of the ESI† file). The MD-refined structure of these compounds T82_cut and T17_cut is described in Fig. 3 and Table S4 of the ESI† file. The binding affinity of DL-predicted compounds would be also revealed *via* FPL calculations. Moreover, the compound T82_22 in the complex with SARS-CoV-2 Mpro was used as the initial structure for DeepFrag prediction because of adopting the largest binding affinity to the protease. Ten compounds, whose names are set as T82_22_*x*, where *x* is the index of the replaced atom, were proposed (*cf.* Fig. S1 of the ESI† file). Two compounds T82_22_16 and T82_22_8 formed a strong interaction with the protease (*cf.* Table S5 of the ESI† file). Furthermore, the DeepFrag package was continuously employed to design 18 modified compounds from the ligands T82_22_16 and T82_22_8, in which these compounds were denoted as T82_22_16_*x* and T82_22_8_*x*, where *x* is the index of the replaced atom (*cf.* Fig. S1 of the ESI† file). The interaction diagram of these ligands with SARS-CoV-2 Mpro was described in Table S6 of the ESI† file. Unfortunately, these compounds formed a lower binding affinity than T82_22_16 and T82_22_8. Therefore, the DeepFrag package would not be used to improve the ligands T82_22_16_*x* and T82_22_8_*x*. In addition, six proposed compounds T117_*x*, where *x* is the index of the replaced atom (*cf.* Table S7 and Fig. S1 of the ESI†) were also predicted. However, the affinity of T117_*x* compounds was not improved comparised to T117.

The obtained binding affinity of the modified ligand *via* FPL simulations is mentioned in Tables 2 and S8 of the ESI† file. The IC^Pre^_50_ of 62 compounds fall in the range from micromolar to nanomolar affinity. 16 compounds formed a strong binding free energy to SARS-CoV-2 Mpro with the IC^Pre^_50_ value in the range of nanomolar value (Table 2). In particular, the pulling work of the top-lead compounds adopted in the range from 105.2 ± 6.8 to 121.6 ± 6.1 kcal mol^−1^ corresponding to the predicted binding free energy ranging from −11.40 to −12.32 kcal mol^−1^. Moreover, the MD-refined structure of the complex was obtained *via* the clustering method with an all-atom cutoff of 0.2 nm. More details in the interaction between the protease and top-lead compounds are shown in Fig. S2 of the ESI† file. In particular, the ligands formed 4.8 ± 0.3 HB and 23.4 ± 0.4 SC contacts to Mpro. Four residues Ser46, His164, Glu166, and Arg188 frequently adopted HB to inhibitors, especially, His164 and Glu165 having contact to >88% ligands. Furthermore, three ligands T82_22, T82_22_40, and T82_22_16_18 gave HB contact with Cys145, which is one of the most important residues located in the binding cavity of the protease. It should be noted that numerous ligands were designed to be able to form a contact with the catalytic dyad (Cys145 and His41) to inhibit the SARS-CoV-2 Mpro biological activity.^9,77,78^ Therefore, it is an additional positive point of the ligands T82_22, T82_22_40, and T82_22_16_18. However, the other ligands also play a potent inhibitor for SARS-CoV-2 Mpro.

**Table 2:** The calculated results of top-lead compounds to SARS-CoV-2 Mpro using DL and FPL calculations

No.	Code	*F*_Max_	*W*	Δ*G*^Pre^_FPL_^a^
1	T82_22_16	953.0 ± 54.0	121.6 ± 6.1	−12.32
2	T82_22_8	940.4 ± 44.8	120.8 ± 2.7	−12.28
3	T82_22_8_14	931.1 ± 28.6	117.0 ± 5.2	−12.06
4	T82_22_30	930.1 ± 39.7	112.4 ± 5.8	−11.81
5	T82_22	870.8 ± 61.6	111.6 ± 6.7	−11.76
6	T82_22_16_40	888.7 ± 39.8	109.7 ± 5.4	−11.65
7	T82_32	857.9 ± 41.9	108.9 ± 3.4	−11.61
8	T82_22_10	881.9 ± 25.3	108.6 ± 3.9	−11.59
9	T82_22_40	919.0 ± 47.6	108.5 ± 5.8	−11.59
10	T82_22_12	818.3 ± 30.5	107.8 ± 3.6	−11.55
11	T82_22_8_24	860.1 ± 45.3	107.6 ± 4.8	−11.54
12	T82_22_16_38	856.3 ± 33.1	106.9 ± 4.0	−11.50
13	T82_22_16_18	856.3 ± 50.3	106.8 ± 6.6	−11.49
14	T82_22_14	835.6 ± 50.8	105.9 ± 5.2	−11.44
15	T82_22_24	880.4 ± 41.2	105.5 ± 2.9	−11.42
16	T82_22_16_10	855.6 ± 58.7	105.2 ± 6.8	−11.40

a The predicted binding free energy Δ*G*^Pre^_FPL_ = −0.056 × *W* − 5.512.^58^ The calculated error is the standard error of the average (SE). The unit of force and energy in pN and kcal mol^−1^, respectively.

Although a compound forms a large binding affinity to SARS-CoV-2 Mpro, the permeability of this compound might be more beneficial in allowing the compound to “meet” the viral protease inside the cells.^9^ The permeability of trial compounds can be predicted *via* logP value,^79^ thus, the logP of designed inhibitors was predicted using PreADMET webserver.^62^ The obtained results are mentioned in Tables S9 and S10 of the ESI† file. Therefore, it may be argued that 11/17 top-lead compounds, which formed nanomolar/sub-micromolar affinity, were suggested to penetrate themselves into the human lung cell and then inhibit viral replication (Table S9 of the ESI† file). Moreover, interestingly, T82 and T17-based compounds showed large solubility, which logP diffuses in the range from −6.35 to −1.32. These compounds would play like α-ketoamide compound 14b, which forms a large binding affinity to SARS-CoV-2 Mpro but is almost inactivated as it inhibits SARS-CoV-2 replication in human lung cells. It is quite reasonable since T82 and T17-based compounds are essentially polysaccharides, which would not adopt much pharmacological potential. However, T56-based compounds formed appropriate permeability with the logP value falls in the range from 1.09 to 2.69 supporting that T56-based compounds can inhibit the SARS-CoV-2 replication in human lung cells. Moreover, HIA and toxicity of the designed inhibitors were also estimated (Table S10 of the ESI† file). The obtained toxicity suggested that all of the designed inhibitors would not poison rats. Besides, all T56-based compounds would be orally absorbed since HIA values are higher than 39%. However, it is hard to orally absorb T17 and T82-based compounds because their HIA values are mostly smaller than 10%.

**Fig. 5:**
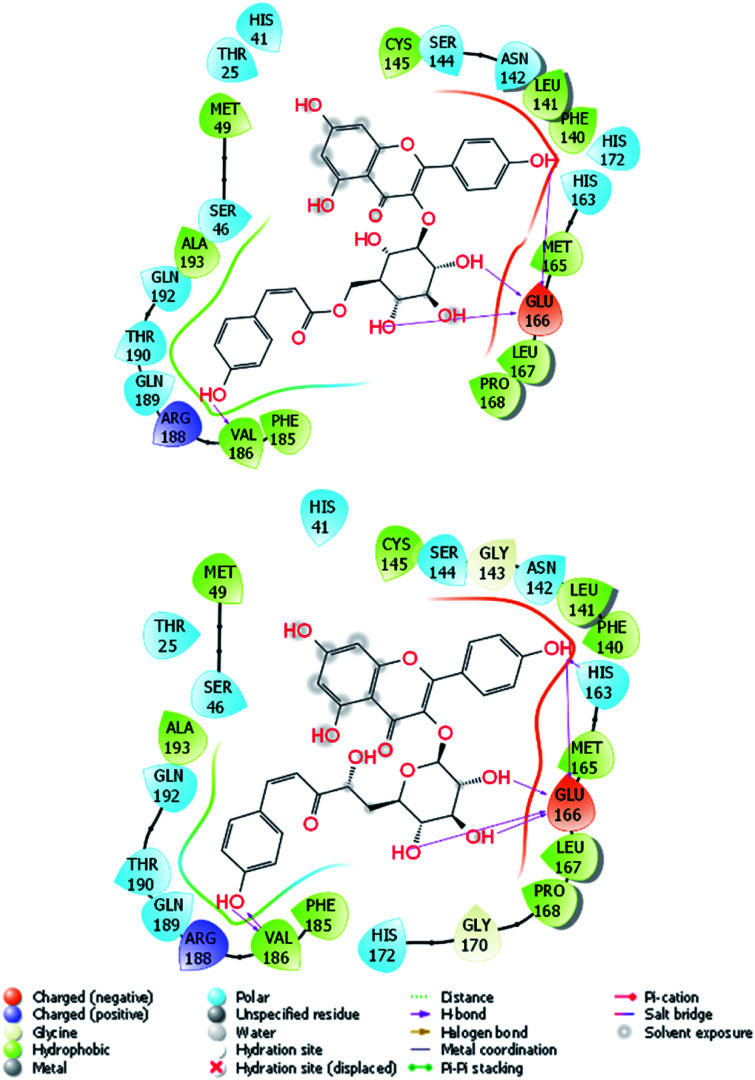
2D interaction diagram of T56_2 and T56_18 compounds to SARS-CoV-2 Mpro. The MD-refined structure of the complexes was obtained using the clustering method with a cutoff of 0.12 nm.

In addition, three T56_*x* compounds including T56_2, T56_18, and T56_8 formed a high-nanomolar affinity to SARS-CoV-2 Mpro (Table 3). In particular, T56_2 and T56_18 bind to the protease with a larger affinity in comparison with the T56 compound, Δ*G*^Pre^_FPL_ = −10.02 kcal mol^−1^. As the interaction diagram in Fig. 5 shows, both T56_2 and T56_18 rigidly formed HBs to Glu166 and Val186 residues. Forming only SC contacts to the Cys145 residue, two compounds probably play as non-covalent binding inhibitors of SARS-CoV-2 Mpro.

**Table 3:** The calculated results of top-lead compounds to SARS-CoV-2 Mpro using DL and FPL calculations

No.	Code	*F*_Max_	*W*	Δ*G*^Pre^_FPL_^a^	IC^Pre^_50_ range^b^
1	T56_2	705.2 ± 18.9	87.9 ± 2.6	−10.43	High-nanomolar
2	T56_18	717.4 ± 51.6	81.8 ± 5.3	−10.09	High-nanomolar
3	T56_8	655.1 ± 22.9	79.7 ± 3.4	−9.98	High-nanomolar

a The predicted binding free energy Δ*G*^Pre^_FPL_ = −0.056 × *W* − 5.512 kcal mol^−1^.^58^

b The predicted IC^Pre^_50_ was calculated *via* the formula 
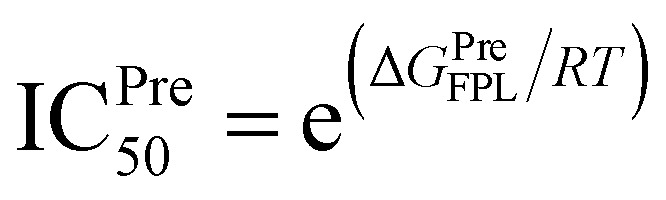
 using hypothesis that IC_50_ equals to inhibition constant *k*_i_. The calculated error is the standard error of the average (SE). The units of force and energy are pN and kcal mol^−1^, respectively.

## Conclusions

Using a reasonable combination of DL calculations and atomistic simulations could lead to a new approach for developing SARS-CoV-2 Mpro inhibitors. In this context, we have demonstrated that natural compounds can bind to SARS-CoV-2 Mpro with a strong binding affinity, which ranges from micromolar to nanomolar values. Tomatine (T82), thevetine (T17), and tribuloside (T56) could form rigid HB and SC contacts to SARS-CoV-2 Mpro. Three compounds thus exhibit nanomolar/high-nanomolar affinities and 14 compounds form a sub-micromolar affinity. However, the permeability of compounds might be advantageous in preventing SARS-CoV-2 replication.^9^ Only 11/17 top-lead compounds were suggested that they can insert themselves into the human lung cell and then inhibit viral replication. These compounds involve tribuloside (T56), quercimeritrin (T117), corilagin (T61), gomphrenin III (T44), proscillaridin (T26), melianotriol (T33), adynerin (T52), hinokiflavone (T3), cynarine (T55), rutin (T126), and melianodiol (T19). The ADME prediction also indicated that they are less toxic substances.

Because tomatine and thevetine are very big compounds with the steroid and triterpenoid saponin groups fully exposed in the solvent, respectively, two truncated compounds T82_cut and T17_cut were proposed by removing the respective groups. Interestingly, two compounds also exhibit strong binding to the protease. Moreover, DL calculations using the DeepFrag package were applied to chemically alter four compounds T82_cut, T17_cut, T56, and T117 with the expectation that the modified compounds would adopt a larger binding affinity. 60 modified compounds were thus suggested. All of the designed compounds formed a large binding affinity to SARS-CoV-2 Mpro, in which Δ*G*^Pre^_FPL_ falls in the range from sub-micromolar to nanomolar affinities. However, only T56 and T117 based compounds adopted an appropriate permeability, suggesting that they are able to inhibit the SARS-CoV-2 replication in the human lung cells. Three modified compounds including T56_2, T56_8, and T56_18 are highly potent inhibitors since adopting high-nanomolar affinities to SARS-CoV-2 Mpro. In addition, the other T56_*x* and T117_*x* compounds inhibit the protease with sub-micromolar affinity. They would thus play the roles of potential inhibitors preventing SARS-CoV-2 replication.

## Conflicts of interest

There are no conflicts to declare.

## Supplementary Material

RA-011-D1RA06534C-s001
