# Supplementary material for: Searching and designing potential inhibitors for SARS-CoV-2 Mpro from natural sources using atomistic and deep-learning calculations
Source: RSC Adv. 2021 Nov 29;11(61):38495–504. doi: 10.1039/d1ra06534c (PMC9044063; doi:10.1039/d1ra06534c)
Supplement: RA-011-D1RA06534C-s001 [file RA-011-D1RA06534C-s001.pdf]

Electronic Supplementary Material (ESI) for RSC Advances.
This journal is © The Royal Society of Chemistry 2021

Searching and Designing Potential Inhibitors for SARS-CoV-2 Mpro from Natural Sources using Atomistic and Deep-Learning Calculations

Nguyen Minh Tam,[ab] Duc-Hung Pham,[c] Dinh Minh Hiep,[d] Phuong-Thao Tran,[e] Duong Tuan Quang,[f]

and Son Tung Ngo[bg*]

[a]Computational Chemistry Research Group, Ton Duc Thang University, Ho Chi Minh City 72915, Vietnam
[b]Faculty of Applied Sciences, Ton Duc Thang University, Ho Chi Minh City 72915, Vietnam
[c]Division of Immunobiology, Cincinnati Children's Hospital Medical Center, Cincinnati, Ohio 45229, United States
[d]Department of Agriculture and Rural Development, Ho Chi Minh City 71007, Vietnam
[e]Hanoi University of Pharmacy, Hanoi 11021, Vietnam
[f]Department of Chemistry, Hue University, Hue City, Thua Thien Hue Province, Vietnam
[g]Laboratory of Theoretical and Computational Biophysics, Ton Duc Thang University, Ho Chi Minh City 72915, Vietnam

*Email: ngosontung@tdtu.edu.vn

Table S1. Docking results of natural compounds.

No.	Compound	PubChem ID	Name	Organism	ΔG_{Dock}
1	T14	65071	Limonin	Citrus	-8.9
2	T82	28523	Tomatine	Tomato	-8.9
3	T13	5281600	Amentoflavone	Garcinia brasiliensis	-8.6
4	T3	5281627	Hinokiflavone	Rhus succedanea	-8.6
5	T180	98570	Allocryptopine	Glaucium arabicum	-8.4
6	T25	31310	Scillaren A	Scilla maritima	-8.3
7	T11	5154	Sanguinarine	Argemone mexicana	-8.2
8	T26	222154	Proscillaridin	Scilla maritima	-8.2
9	T107	4970	Protopine	Corydalis ambigua	-8.1
10	T20	167691	Peiminine	Fritillaria roylei	-8.1
11	T52	441840	Adynerin	Nerium oleander	-8.1
12	T61	73568	Corilagin	Sapium sebiferum	-8.1
13	T65	131900	Peimine	Fritillaria roylei	-8.1
14	T121	32024	Alpha-antiarin	Antiaris toxicaria	-7.9
15	T202	441295	Ginkgolide_C	Ginkgo biloba	-7.9
16	T24	5317157	Equisetrin	Equisetum arvense	-7.9
17	T56	10175330	Tribuloside	Tribulus terrestris	-7.9
18	T19	10028469	Melianodiol	Melia azedarach	-7.8
19	T27	11013	Rhodexin A	Rohdea japonica	-7.8
20	T35	3083631	Chlorogenin	Tribulus terrestris	-7.8
21	T50	119041	Obacunone	Phellodendron amurense	-7.8
22	T102	442814	Pachyrrhizone	Pachyrhizus erosus	-7.7
23	T115	15515703	Jujubogenin	Ziziphus jujuba	-7.7
24	T117	5282160	Quercimeritrin	Hibiscus mutabilis	-7.7
25	T179	73432	Brusatol	Brucea javanica	-7.7
26	T17	159331	Thevetine	Thevetia peruviana	-7.7
27	T182	3032482	Ecdysterone	Achyranthes bidentata	-7.7
28	T1	31342	Solasodine	Solanum xanthocarpum	-7.7
29	T23	72307	Sesamin	Acanthopanax aculeatus	-7.7
30	T33	185586	Melianotriol	Melia azedarach	-7.7
31	T34	185617	Scutellarin	Scutellaria baicalensis	-7.7
32	T55	5316647	Cynarine	Asteraceae	-7.7
33	T7	5270604	Taraxasterol	Centipeda minima	-7.7
34	T8	91453	Hecogenin	Agave americana	-7.7
35	T126	5280805	Rutin	Sophora japonica	-7.6
36	T30	470259	Arnidiol	Centipeda minima	-7.6
37	T44	6325292	Gomphrenin_III	Gomphrena globosa	-7.6
38	T4	15560423	Kulactone	Melia azedarach	-7.6
39	T58	10494	Oleanolic acid	Achyranthes bidentata	-7.6
40	W17	3000706	Valinomycin	Streptomyces	-7.6
41	T119	65064	(-)-epigallocatechin 3-gallate	Green tea	-7.5

42	T114	472761	Tirucallol	Euphorbia thymifolia	-7.5
43	T120	163776	Ginkgolide J	Ginkgo biloba	-7.5
44	T166	5281867	Savinin	Acanthopanax aculeatus	-7.5
45	T207	5281643	Hyperin	Polygonum hydropiper	-7.5
46	T228	9851101	Toosendanin	Melia azedarach	-7.5
47	T29	73145	Beta-amyrin	Euphorbia antiquorum	-7.5
48	T42	441867	Neriifolin	Thevetia peruviana	-7.5
49	T5	92095	Sarsasapogenin	Smilax glabra	-7.5
50	T84	99516	Tigogenin	Agave americana	-7.5
51	T88	112500418	Amaranthin	Gomphrena globosa	-7.5
52	T89	5280441	Vitexin	Crotalaria mucronata	-7.5
53	T10	5318868	Kulolactone	Melia azedarach	-7.4
54	T116	5281807	Puerarin	Pueraria thomsoni	-7.4
55	T162	442015	Columbin	Tinospora sagittata	-7.4
56	T28	73073	Serpentine	Rauwolfia serpentina	-7.4
57	T2	99474	Diosgenin	Schizocapsa plantaginea	-7.4
58	T64	124061	Sophoradiol	Sophora japonica	-7.4
59	T6	160270	Dracorubin	Daemonorops draco	-7.4
60	T83	64945	Ursolic acid	Lamiaceae	-7.4
61	K9	53481915	Adenosine thiamine diphosphate	Cordyceps	-7.3
62	T108	5770	Reserpine	Rauwolfia serpentina	-7.3
63	T12	92097	Taraxerol	Taraxacum officinale	-7.3
64	T161	441828	Inokosterone	Achyranthes bidentata	-7.3
65	T169	6419993	Ginkgolide A	Ginkgo biloba	-7.3
66	T174	5490064	Avicularin	Polygonum aviculare	-7.3
67	T209	6325730	Neocycasin D	Cycas revoluta	-7.3
68	T21	5319341	Melianol	Melia azedarach	-7.3
69	T38	5281847	Rottlerin	Mallotus philippinensis	-7.3
70	T39	5318070	Holarrhenine	Holarrhena antidysenterica	-7.3
71	T40	5320199	Noracronycine	Glycosmis pentaphylla	-7.3
72	T47	14749	Carminic acid	Laccifer lacca	-7.3
73	T111	72326	Betulin	Sophora japonica	-7.2
74	T124	14833699	Canavalia gibberellin I	Canavalia gladiata	-7.2
75	T128	6451060	Ovatodiolide	Anisomeles ovata	-7.2
76	T135	6324617	Ginkgolide B	Ginkgo biloba	-7.2
77	T137	160006	Bruceine A	Brucea javanica	-7.2
78	T16	382580	Cycloartenol	Euphorbia antiquorum	-7.2
79	T192	5280804	Isoquercitrin	Equisetum arvense	-7.2
80	T217	161496	Bruceine B	Brucea javanica	-7.2
81	T37	5280704	Cosmosiine	Euphorbia thymifolia	-7.2
82	T43	503737	Liquiritin	Glycyrrhiza uralensis	-7.2
83	T46	6758	Rotenone	Pachyrhizus erosus	-7.2
84	T60	73299	Hederagenin	Aristolochiaceae	-7.2

85	T68	5281647	Mangiferin	Mangifera indica	-7.2
86	T72	44559213	Glochidiol	Glochidion eriocarpum	-7.2
87	T79	5281813	Wedelolactone	Wedelia calendulacea	-7.2
88	T93	44559215	Glochidonol	Glochidion eriocarpum	-7.2
89	W12	122844	Betulonic acid	Liquidambar formosana	-7.2
90	T101	44567124	Kulinone	Melia azedarach	-7.1
91	T141	5281810	Tectoridin	Belamcanda chinensis	-7.1
92	T15	174863	Lactucopicrin	Lactuca indica	-7.1
93	T170	10211	Byakangelicin	Angelica dahurica	-7.1
94	T31	10246509	Berberine	Argemone mexicana	-7.1
95	T41	160644	Liensinine	Nelumbo nucifera	-7.1
96	T70	5458462	Cycloartocarpin	Artocarpus heterophyllus	-7.1
97	T109	64971	Betulinic acid	Ziziphus jujuba	-7
98	T167	5318980	Icaritin	Herba epimedii	-7
99	T198	10607	Podophyllotoxin	Podophyllum tonkinense	-7
100	T66	151289	Evodiamine	Evodia rutaecarpa	-7
101	T80	6452523	Corybulbine	Corydalis ambigua	-7
102	T81	12314446	Tumulosic acid	Poria cocos	-7
103	T99	72323	Jatrorrhizine	Fibraurea tinctoria	-7
104	W13	442879	Hinokinin	Aristolochia odoratissima	-7
105	T105	11968340	Carthamin	Carthamus tinctorius	-6.9
106	T106	54676038	Dicumarol	Melilotus suaveolens	-6.9
107	T142	5281855	Ellagic acid	Terminalia chebula	-6.9
108	T160	73337	Magnoflorine	Herba epimedii	-6.9
109	T36	6325294	Gomphrenin V	Gomphrena globosa	-6.9
110	T48	73078	Tetrandrine	Radix stephaniae	-6.9
111	T49	114909	Tephrosin	Pachyrhizus erosus	-6.9
112	W9	47936	Forskolin	Plectranthus barbatus	-6.9
113	K41	5326875	Biotinyl-5'-AMP	Cordyceps	-6.8
114	T123	442514	Hematoxylin	Caesalpinia sappan	-6.8
115	T130	135403802	Hematein	Caesalpinia sappan	-6.8
116	T139	5280445	Luteolin	Lonicera japonica	-6.8
117	T150	345501	Deoxypodophyllotoxin	Podophyllum tonkinense	-6.8
118	T151	441080	Ajmaline	Rauwolfia serpentina	-6.8
119	T152	14833701	Canavalia gibberellin II	Canavalia gladiata	-6.8
120	T158	64981	Arctigenin	Arctium lappa	-6.8
121	T171	5280343	Quercetin	Prunus armeniaca	-6.8
122	T173	5384083	Bruceine C	Brucea javanica	-6.8
123	T188	11870307	Chlorogenic acid	Boehmeria nivea	-6.8
124	T194	5281672	Myricetin	Thuja orientalis	-6.8
125	T216	88708	Gentiopicrin	Gentiana scabra	-6.8
126	T220	9576780	Macrozamin	Cycas revoluta	-6.8
127	T22	65752	Rutecarpine	Evodia rutaecarpa	-6.8

128	T32	73191	Nodakenin	Peucedanum decursivum	-6.8
129	T62	100528	Arctiin	Arctium lappa	-6.8
130	W14	44427466	4,4'-O-benzoylisolariciresinol	Chamaecyparis obtusa	-6.8
131	W16	4477	Niclosamide	Mammalian mitochondria	-6.8
132	T100	101301	Corydaline	Corydalis ambigua	-6.7
133	T110	68827	Artemisinine	Artemisia annua	-6.7
134	T112	72378	Lycorine	Narcissus tazetta	-6.7
135	T131	10207	Aloe Emodin	Rheum rhabarbarum	-6.7
136	T140	5281612	Diosmetin	Capsella bursa-pastori	-6.7
137	T146	6293	Alizarin	Rubia cordifolia	-6.7
138	T185	5320070	Neocycasin C	Cycas revoluta	-6.7
139	T187	6450453	Anomalin	Angelica dahurica	-6.7
140	T200	73467	Verbenalin	Verbena officinalis	-6.7
141	T214	12305761	Barbaloin	Aloe	-6.7
142	T221	22524404	Neocycasin B	Cycas revoluta	-6.7
143	T239	5320072	Neocycasin E	Cycas revoluta	-6.7
144	T248	6167	Colchicine	Colchicum autumnale	-6.7
145	T57	3503	Gossypol	Gossypium	-6.7
146	T75	10144	Liriodenine	Nelumbo nucifera	-6.7
147	T85	119204	Roemerine	Nelumbo nucifera	-6.7
148	T86	76330309	Conckurchine	Holarrhena antidysenterica	-6.7
149	T87	5321206	Sebiferic acid	Sapium sebiferum	-6.7
150	T91	11854305	beta-Elemonic acid	Poria cocos	-6.7
151	T92	22524408	Neocycasin G	Cycas revoluta	-6.7
152	T94	3220	Emodin	Rheum rhabarbarum	-6.7
153	T96	10639	Physcione	Cassia occidentalis	-6.7
154	W7	2751794	6,7-Dehydroroyleanone	Tetradenia riparia	-6.7
155	T129	12308618	Artocarpetin	Artocarpus heterophyllus	-6.6
156	T138	222515	Brasilin	Caesalpinia sappan	-6.6
157	T218	5274587	Lotusine	Nelumbo nucifera	-6.6
158	T223	5281408	Rhyncophylline	Uncaria rhynchophylla	-6.6
159	T224	5281756	Mucronatine	Crotalaria mucronata	-6.6
160	T53	614726	Lycorin	Crinum asiaticum	-6.6
161	T69	5316036	Conessimine	Holarrhena antidysenterica	-6.6
162	T71	5484385	Pachymic acid	Poria cocos	-6.6
163	T90	5458461	Artocarpin	Artocarpus heterophyllus	-6.6
164	T9	114917	Tanshinone	Salvia miltiorrhiza	-6.6
165	K2	5375662	9-(beta-D-Ribofuranosyl)zeatin	Cordyceps	-6.5
166	K7	44123306	Adenosine 5'-propyl phosphate	Cordyceps	-6.5
167	T103	5281605	Baicalein	Scutellaria baicalensis	-6.5
168	T113	160597	Anonaine	Annona squamosa	-6.5
169	T122	72301	Hyndarine	Stephania rotunda	-6.5
170	T133	41169	Nornuciferine	Nelumbo nucifera	-6.5

171	T143	54684703	Equisetin	Equisetum arvense	-6.5
172	T175	6713959	Gelsemine	Gelsemium elegans	-6.5
173	T186	6436877	Agathic acid	Erythrophleum fordii	-6.5
174	T193	5280863	Kaempferol	Ginkgo biloba	-6.5
175	T203	200480	Pronuciferine	Nelumbo nucifera	-6.5
176	T206	3055167	Byakangelicol	Angelica dahurica	-6.5
177	T51	441082	Conessine	Holarrhena antidysenterica	-6.5
178	T77	353825	Graveoline	Ruta graveolens	-6.5
179	W3	94162	Sugiol	Calocedrus formosana	-6.5
180	K15	20849086	Succinyladenosine	Cordyceps	-6.4
181	T118	5320073	Neocycasin F	Cycas revoluta	-6.4
182	T125	5280443	Apigenin	Selaginella tamariscina	-6.4
183	T172	5281666	Kaempferide	Alpinia officinarum	-6.4
184	T176	10146	Nuciferine	Nelumbo nucifera	-6.4
185	T184	5281654	Isorhamnetin	Polygonum hydropiper	-6.4
186	T196	5320945	Rhamnazin	Polygonum hydropiper	-6.4
187	T201	98608	Phellopterin	Angelica dahurica	-6.4
188	T45	11772257	Isoconessimine	Holarrhena antidysenterica	-6.4
189	T78	5281650	Mangostin	Garcinia mangostana	-6.4
190	T95	10208	Chrysophanol	Rheum rhabarbarum	-6.4
191	W1	442027	Ferruginol	Chamaecyparis obtusa	-6.4
192	T155	969516	Curcumin	Curcuma longa	-6.4
193	W8	44427461	Diacetoxyabieta	Juniperus formosana	-6.4
194	K21	135501639	N2,N2-Dimethylguanosine	Cordyceps	-6.3
195	T165	5281708	Daidzein	Pueraria thomsoni	-6.3
196	T195	5317436	Alloisoimperatorin	Angelica dahurica	-6.3
197	T236	91520	Catalpol	Rehmannia glutinosa	-6.3
198	T237	124375	Glucogallin	Rheum rhabarbarum	-6.3
199	T258	245005	Aconitine	Aconitum napellus	-6.3
200	T63	101729	Cyclolaudenol	Marsilea quadrifolia	-6.3
201	T73	2543	Cannabinol	Cannabis sativa	-6.3
202	T97	23035	Leucoindigo	Indigo pulverata levis	-6.3
203	W2	11289118	Dehydroabieta-7-one	Chamaecyparis obtusa	-6.3
204	K3	127187	N6-Hydroxymethyladenosine	Cordyceps	-6.2
205	K8	2055	N-Phenyl-alpha-cyano-3,4-dihydroxycinnamamide	Cordyceps	-6.2
206	T156	6688	1,4-Dihydroxyanthraquinone	Rubia cordifolia	-6.2
207	T157	63224	Febrifugine	Dichroa febrifuga	-6.2
208	T177	10212	Imperatorin	Angelica dahurica	-6.2
209	T54	5281607	Chrysin	Oroxylum indicum	-6.2
210	T74	10166	Eleutherin	Eleutherine subaphylla	-6.2
211	W5	44427460	8-Hydroxyabieta-9(11),13-diene-12-one	Chamaecyparis obtusa	-6.2
212	K16	65095	1-Methylinosine	Cordyceps	-6.1

213	K4	96373	1-Methylguanosine	Cordyceps	-6.1
214	T127	5281376	Lathyrol	Euphorbia lathyris	-6.1
215	T168	9851693	Isofebrifugine	Dichroa febrifuga	-6.1
216	T178	33306	Oxypeucadanin	Angelica dahurica	-6.1
217	T208	5281811	Tectorigenin	Belamcanda chinensis	-6.1
218	T226	5321124	Sappanin	Caesalpinia sappan	-6.1
219	T231	174174	Atropine	Datura metel	-6.1
220	T251	182140	Hypophyllanthin	Phyllanthus urinaria	-6.1
221	T257	182644	Nirtetralin	Phyllanthus urinaria	-6.1
222	T265	220401	Colchamine	Colchicum autumnale	-6.1
223	T59	10569	Abietic acid	Resina Pini-Colophonium	-6.1
224	T67	5281616	Galangin	Alpinia officinarum	-6.1
225	W6	14827260	7beta-hydroxydeoxycryptojaponol	Cryptomeria japonica	-6.1
226	K1	441300	Abacavir	Cordyceps	-6
227	K28	135398508	Entecavir	Cordyceps	-6
228	K35	13342874	(S)-5'-Deoxy-5'-(methylsulfinyl)adenosine	Cordyceps	-6
229	T132	26305	Nodakenetin	Peucedanum decursivum	-6
230	T148	63123	Arborine	Glycosmis pentaphylla	-6
231	T163	638024	Piperine	Piper nigrum	-6
232	T197	5184	Scopolamine	Datura metel	-6
233	T222	92769	Indican	Polygonum perfoliatum	-6
234	T240	6321388	Lasiocarpine	Heliotropium indicum	-6
235	T270	5281441	Enhydrin	Enydra fluctuans	-6
236	T76	101616	Lobeline	Lobelia pyramidalis	-6
237	W4	11724205	Cryptojaponol	Cryptomeria japonica	-6
238	K17	439946	2-Methylaminoadenosine	Cordyceps	-5.9
239	K23	107461	N4-Acetylcytidine	Cordyceps	-5.9
240	K24	102175	N6-Methyladenosine	Cordyceps	-5.9
241	K30	440569	Imidazoleacetic acid riboside	Cordyceps	-5.9
242	K31	440004	N6,N6-Dimethyladenosine	Cordyceps	-5.9
243	K5	65102	3'-Amino-3'-deoxyadenosine	Cordyceps	-5.9
244	K6	10492375	5-Carboxy-2'-deoxyuridine	Cordyceps	-5.9
245	T134	73174	Dehydrocostus lactone	Radix saussureae lappae	-5.9
246	T145	23242585	Odorine	Allium odorum	-5.9
247	T153	5318874	Angenomalin	Angelica dahurica	-5.9
248	T183	5281555	Pyrethrin II	Chrysanthemum	-5.9
249	T190	68081	Isoimperatorin	Angelica dahurica	-5.9
250	T191	285698	Matrine	Sophora flavescens	-5.9
251	T256	161464	Leonurine	Leonurus sibiricus	-5.9
252	T98	135426527	Glycosminine	Glycosmis pentaphylla	-5.9
253	K11	92751	Orotidine	Cordyceps	-5.8
254	K18	135601234	5’-Methylthioinosine	Cordyceps	-5.8
255	K26	443234	5’-Dehydroadenosine	Cordyceps	-5.8

256	K27	439176	5’-Methylthioadenosine	Cordyceps	-5.8
257	K36	46173772	Deoxynucleotide	Cordyceps	-5.8
258	K40	135398641	Inosine	Cordyceps	-5.8
259	T104	6433154	Pyrethrin I	Chrysanthemum	-5.8
260	T204	637858	Piperlongumine	Piper longum	-5.8
261	T230	10227	Kokusaginine	Glycosmis pentaphylla	-5.8
262	T255	4990	Anginin	Vigna cylindrica	-5.8
263	K10	135398593	2’-Deoxyinosine	Cordyceps	-5.7
264	K14	72200	2-Aminoadenosine	Cordyceps	-5.7
265	K25	60961	Adenosine	Cordyceps	-5.7
266	K32	159269	Telbivudine	Cordyceps	-5.7
267	K34	46173721	Pyrimidine 5'-nucleotide	Cordyceps	-5.7
268	T144	10362168	Angelol G	Angelica pubescens	-5.7
269	T210	6436243	Costus lactone	Radix saussureae lappae	-5.7
270	T219	5365703	Ambrettolid	Hibiscus abelmoschus	-5.7
271	T227	12314211	Phyllantidine	Phyllanthus urinaria	-5.7
272	T238	571784	Phyllanthine	Phyllanthus urinaria	-5.7
273	T259	440936	Arbutin	Gaultheria fragrantissima	-5.7
274	W15	72303	Honokiol	Magnolia officinalis	-5.7
275	K33	1835	5-Methyldeoxycytidine	Cordyceps	-5.6
276	T149	120697	Eleutherol	Eleutherine subaphylla	-5.6
277	T199	10834	Tropacocaine	Erythroxylum coca	-5.6
278	T205	1548943	Capsaicin	Capsicum annuum	-5.6
279	T233	5370536	Piperic acid	Piper nigrum	-5.6
280	T234	2355	Bergapten	Angelica sinensis	-5.6
281	K20	104762	Mizoribine	Cordyceps	-5.5
282	T242	6303	Cordycepin	Cordyceps	-5.5
283	T159	72300	Magnolol	Magnolia officinalis	-5.5
284	T215	10228	Osthol	Cnidium monnieri	-5.5
285	T229	6760	Skimmianine	Ruta graveolens	-5.5
286	T235	10205	Plumbagin	Plumbago zeylanica	-5.5
287	T282	637775	Sinapinic acid	Brassica juncea	-5.5
288	K13	5789	Thymidine	Cordyceps	-5.4
289	K19	13712	Deoxyuridine	Cordyceps	-5.4
290	K22	6029	Uridine	Cordyceps	-5.4
291	K38	99592	3-Methyluridine	Cordyceps	-5.4
292	T164	5281226	Bixin	Bixa orellana	-5.4
293	T213	5478166	Urushiol	Rhus succedanea	-5.4
294	T241	1148	DL-Tryptophan	Vigna cylindrica	-5.4
295	T246	5280460	Scopoletin	Angelica dahurica	-5.4
296	T263	3806	Juglone	Juglans regia	-5.4
297	K12	15047	Pseudouridine	Cordyceps	-5.3
298	T292	40539	Quisqualic acid	Quisqualis indica	-5.3

299	W10	44427462	Cedrane-3beta,12-diol	Juniperus formosana	-5.3
300	K29	6175	Cytidine	Cordyceps	-5.2
301	T136	101549	Vetiverol	Vetiveria zizanioides	-5.2
302	T181	273569	Alpha-Cyperol	Cyperus rotundus	-5.2
303	T211	160570	Cannabidiolic acid	Cannabis sativa	-5.2
304	T212	5281232	Crocetin	Gardenia jasminoides	-5.2
305	T262	2775	Citropten	Citrus medica L. var. sarcodactylis	-5.2
306	T291	10742	Syringic	Kalanchoe pinnata	-5.2
307	T295	5459896	Cycasin	Cycas revoluta	-5.2
308	K39	439714	Allantoin	Cordyceps	-5.1
309	T225	5316107	Costol	Radix saussureae lappae	-5.1
310	T243	6755	Lawsone	Lawsonia inermis	-5.1
311	T252	442106	Hypaphorine	Erythrina orientalis	-5.1
312	T253	521903	Patchouli alcohol	Pogostemon cablin	-5.1
313	T267	6047	Levodopa	Vicia faba	-5.1
314	T275	689043	Caffeic acid	Kalanchoe pinnata	-5.1
315	W11	10398656	Alpha-Cadinol	Chamaecyparis obtusa	-5.1
316	T232	5281794	Shogaol	Zingiber officinale	-5
317	T249	10235	Cytisine	Sophora flavescens	-5
318	T250	131273	3-Heptadecadienyl pyrocatechol	Rhus succedanea	-5
319	T272	370	Gallic acid	Terminalia chebula	-5
320	T277	994	Polyphenylalanine	Vigna cylindrica	-5
321	T285	440473	Leucenol	Leucaena glauca	-5
322	T289	3862	Mimosine	Mimosa pudica	-5
323	K37	439920	Pyrimidine nucleoside	Cordyceps	-4.9
324	T273	10659	Apiole	Petroselinum sativum	-4.9
325	T290	5816	Adrenalin	Ephedra sinica	-4.9
326	T304	10772	Coixol	Coix lacryma-jobi	-4.9
327	T330	525	Malic acid	Tomato	-4.9
328	T189	10657	Beta-Cadinene	Pogostemon cablin	-4.8
329	T254	5352899	Butylidenephthalide	Angelica sinensis	-4.8
330	T260	5281426	Umbelliferone	Angelica dahurica	-4.8
331	T266	4276	Myristicin	Myristica fragrans	-4.8
332	T274	31211	Zingerone	Zingiber officinale	-4.8
333	T281	636822	Alpha-Asarone	Acorus gramineus	-4.8
334	T288	3314	Eugenol	Camellia sasanqua	-4.8
335	T298	439260	Norepinephrine	Banana	-4.8
336	T245	445070	Farnesol	Acacia farnesiana	-4.7
337	T268	27694	N-Methylheliamine	Nelumbo nucifera	-4.7
338	T283	5144	Safrole	Angelica sinensis	-4.7
339	T286	596375	Chavibetol	Piper betle	-4.7
340	T309	681	Dopamine	Banana	-4.7
341	T313	6274	Histidine	Vigna cylindrica	-4.7

342	T261	5315981	Cnidium lactone	Ligusticum wallichii	-4.6
343	T296	942	Nicotine	Niconitana tabacum	-4.6
344	T302	9294	Ephedrine	Sida rhombifolia	-4.6
345	T305	11092	Paeonol	Paeonia suffruticosa	-4.6
346	T306	66654	Xanthoxyline	Sapium sebiferum	-4.6
347	T310	8815	Estragole	Zanthoxylum nitidum	-4.6
348	T276	5314067	Nothosmyrnol	Rhizoma et Radix Ligustici jeholensis	-4.5
349	T279	10364	Carvacrol	Coleus aromaticus	-4.5
350	T280	441070	Cuscohygrine	Erythroxylum coca	-4.5
351	T300	4133	Methyl salicylate	Acacia farnesiana	-4.5
352	T308	524874	3,5-Dimethoxyallylbenzene	Rhizoma et Radix Ligustici jeholensis	-4.5
353	T311	91460	Ecgonine	Erythroxylum coca	-4.5
354	T312	243	Benzoic acid	Paeonia lactiflora	-4.5
355	T316	26934	Norephedrine	Ephedra sinica	-4.5
356	T325	3532	Guvacine	Areca catechu	-4.5
357	T328	236	DL-Asparagine	Taraxacum officinale	-4.5
358	T244	10545	Ascaridole	Chenopodium ambrosioides	-4.4
359	T269	110680	Hydnocarpic acid	Hydnocarpus anthelmintica	-4.4
360	T271	5315165	Ligusticumic acid	Angelica sinensis	-4.4
361	T299	412	Nornicotine	Niconitana tabacum	-4.4
362	T303	10666	Ricinine	Ricinus communis	-4.4
363	T307	68148	Chavicol	Piper betle	-4.4
364	T329	275	DL-Canavanine	Canavalia gladiata	-4.4
365	T247	5282855	Gorlic acid	Hydnocarpus anthelmintica	-4.3
366	T294	5282942	Ricinoleic acid	Ricinus communis	-4.3
367	T314	938	Nicotinic acid	Niconitana tabacum	-4.3
368	T315	10355	Arecaidine	Areca catechu	-4.3
369	T318	68289	Sesamol	Sesamum orientale	-4.3
370	T278	7439	Carvone	Mentha arvensis	-4.2
371	T322	857	DL-Leucine	Sauropus androgynus	-4.2
372	T331	1182	DL-Valine	Vigna cylindrica	-4.2
373	T332	11096	Pseudopelletierine	Punica granatum	-4.2
374	T319	86786	N-Methylisopelletierine	Punica granatum	-4.1
375	T320	637566	Geraniol	Pelargonium roseum	-4.1
376	T327	160492	Guvacoline	Areca catechu	-4.1
377	T264	72853	Chaulmoogric acid	Hydnocarpus anthelmintica	-4
378	T284	14525	Fenchone	Thuja orientalis	-4
379	T297	1254	Menthol	Mentha arvensis	-4
380	T317	64685	Borneol	Blumea balsamifera	-4
381	T321	5570	Trigonelline	Cycas revoluta	-4
382	T324	2230	Arecoline	Areca catechu	-4
383	T339	602	Alanine	Vigna cylindrica	-4

384	T287	5352710	Aplotaxene	Radix saussureae lappae	-3.9
385	T301	8842	Citronellol	Pelargonium roseum	-3.9
386	T323	866	DL-Lysine	Sauropus androgynus	-3.9
387	T326	7794	Citronellal	Zanthoxylum avicennae	-3.9
388	T334	125468	Tiglic acid	Croton tiglium	-3.9
389	T337	637090	Crotonic acid	Croton tiglium	-3.9
390	T333	92987	Isopelletierine	Punica granatum	-3.8
391	T336	876	Methionine	Vigna cylindrica	-3.8
392	T293	160788	Dihydrosterculic acid	Euphoria longana	-3.7
393	T335	440933	Hygrine	Erythroxylum coca	-3.6
394	T338	12558	Pyrrolizidine	Heliotropium indicum	-3.3
395	T154	247	Betaine	Lycium sinense	-3.2
396	T147	65036	Allicin	Allium sativum	-3.1
397	T18	8082	Piperidine	Piper nigrum	-3.1

Table S2. Interaction diagram of SARS-CoV-2 Mpro + ligands from docking and MD-refined structures.

<table>
<tr><th>N⁰</th><th>Name</th><th>Docking structure</th><th>MD-refined structure</th></tr>
<tr><td>1</td><td>T82</td><td>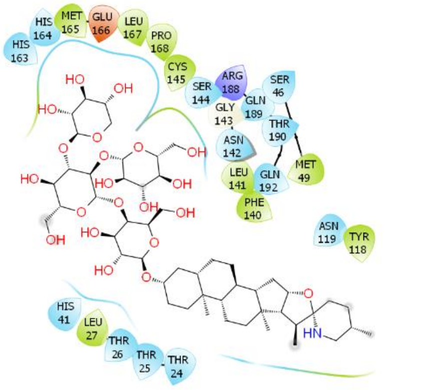
</td><td>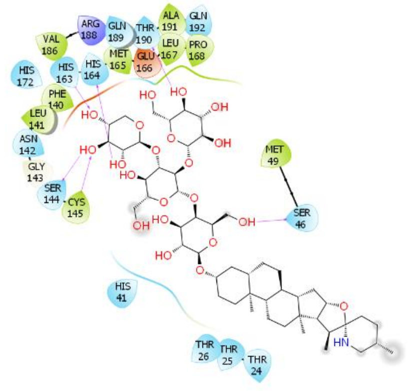
</td></tr>
<tr><td>2</td><td>T17</td><td>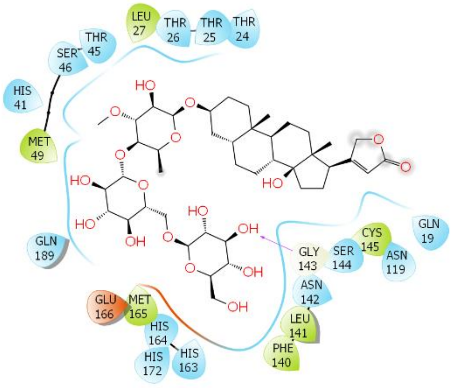
</td><td>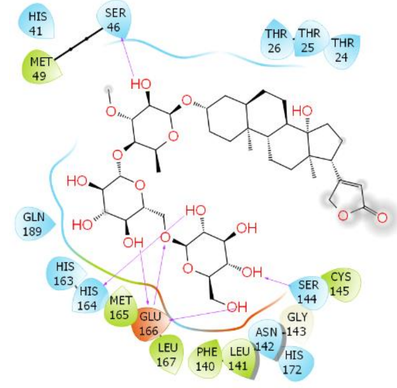
</td></tr>
<tr><td>3</td><td>T56</td><td>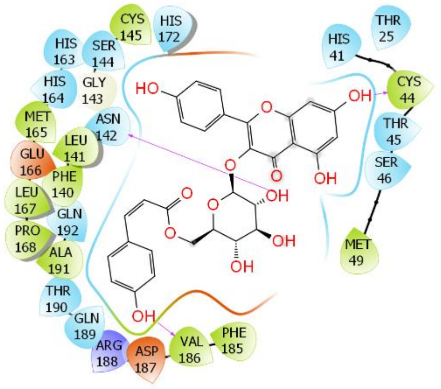
</td><td>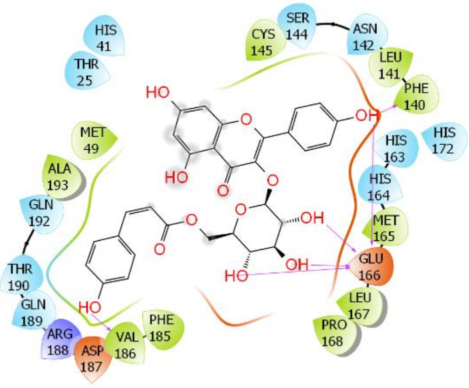
</td></tr>
<tr><td>4</td><td>T117</td><td>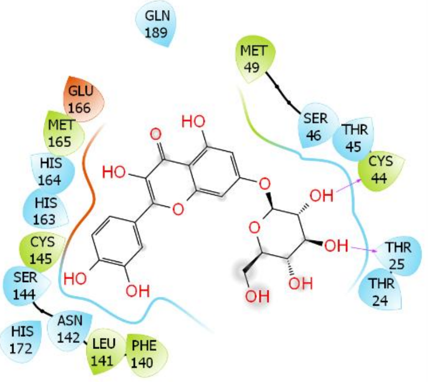
</td><td>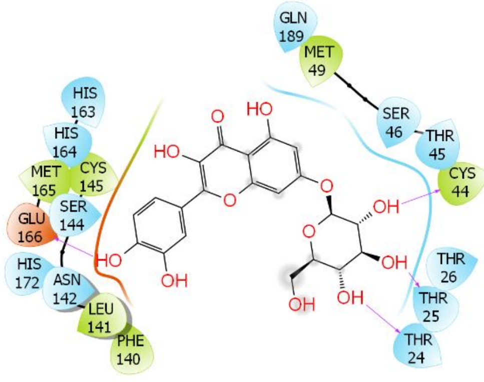
</td></tr>
</table>

5	**T61**	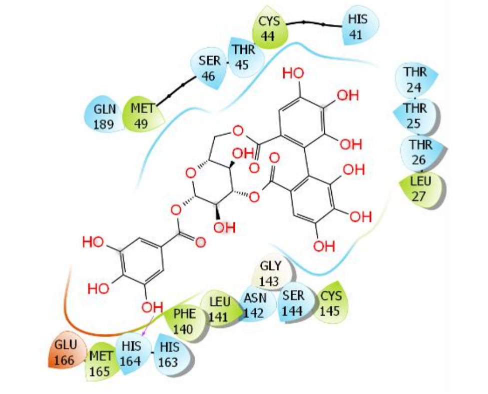	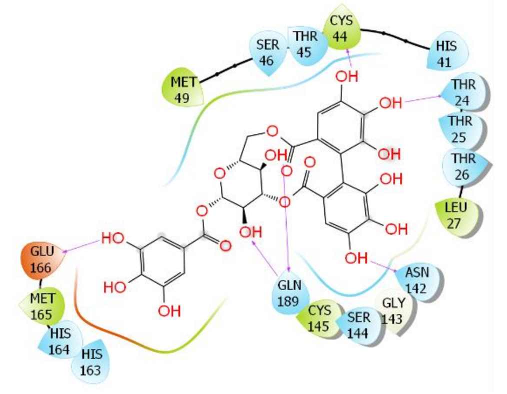
6	**T25**	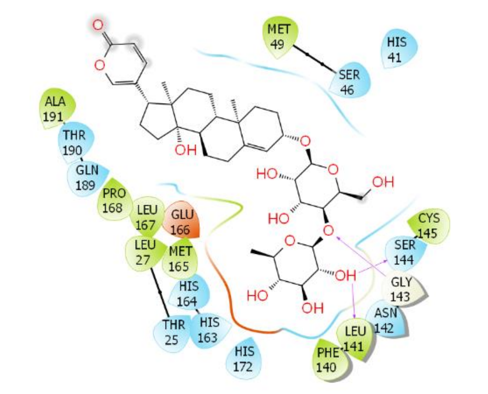	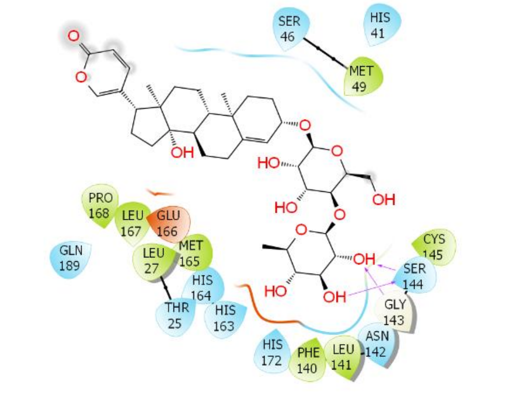
7	**T44**	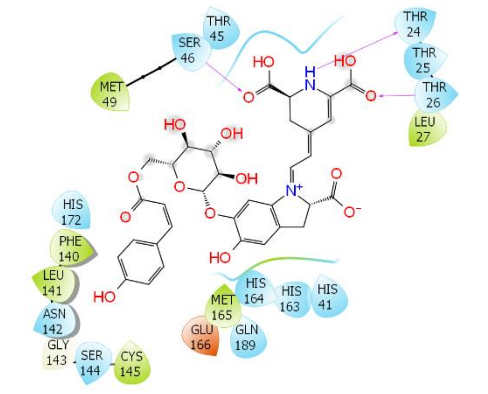	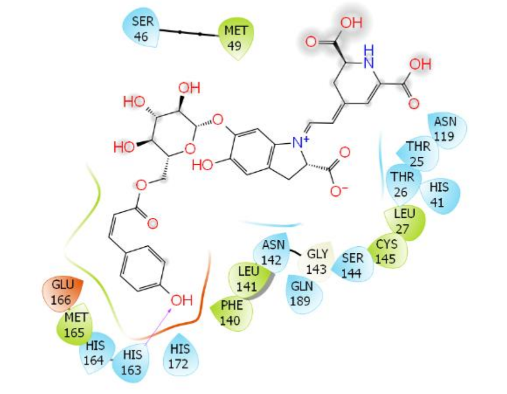
8	**T26**	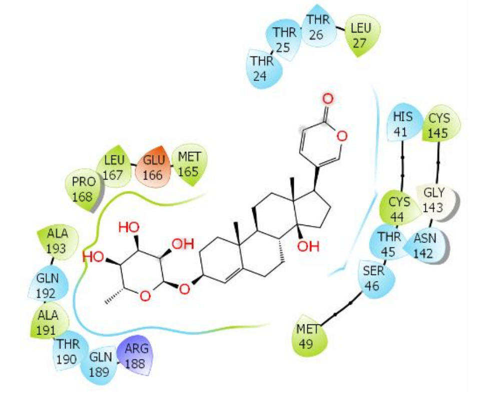	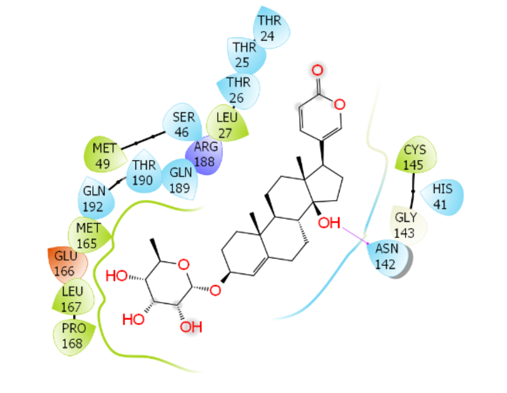

9	**T33**	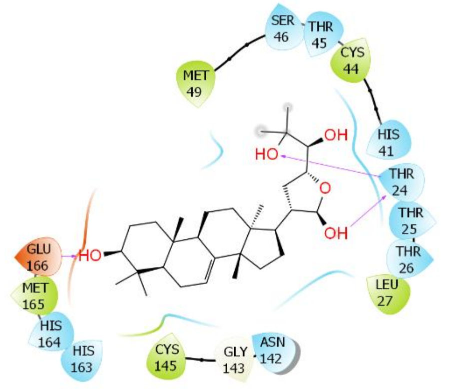	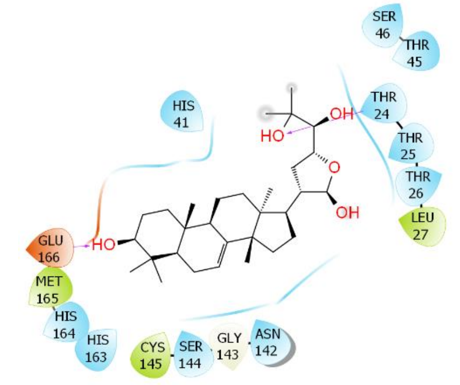
10	**T52**	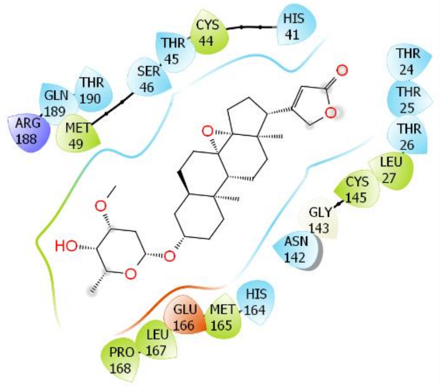	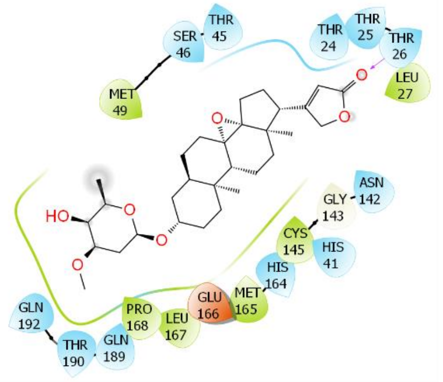
11	**T24**	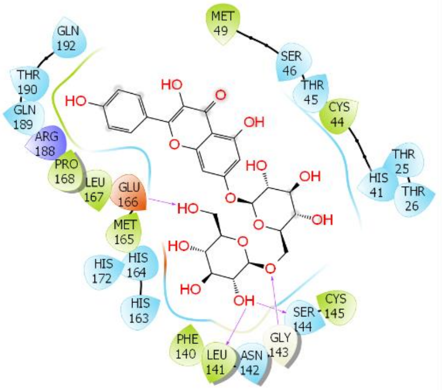	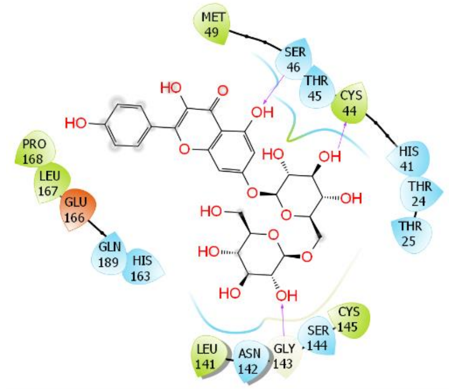
12	**T3**	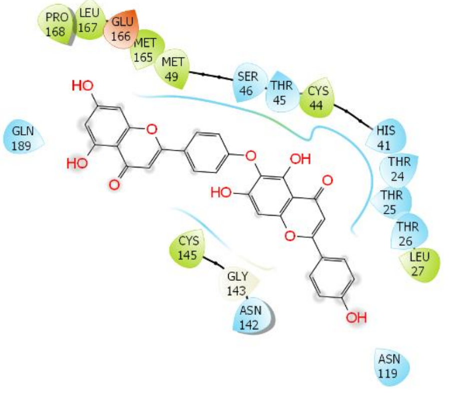	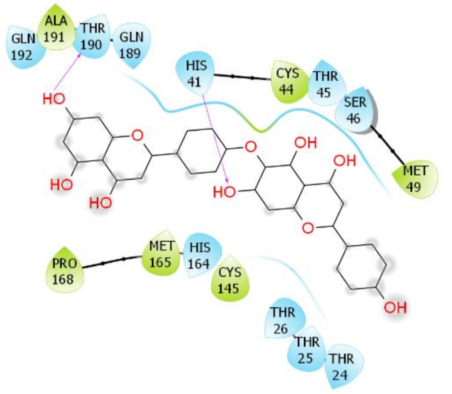

13	**T55**	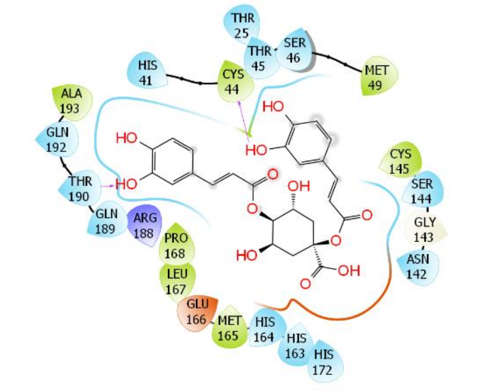	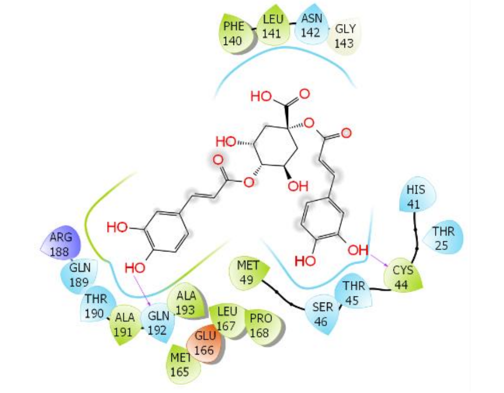
14	**T202**	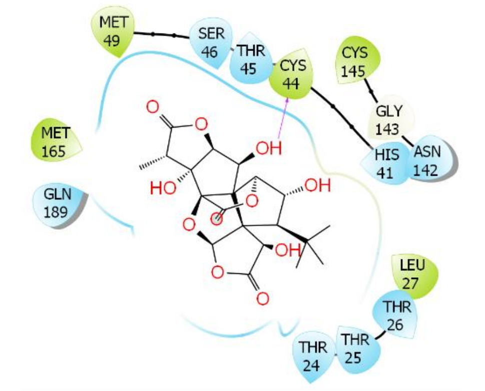	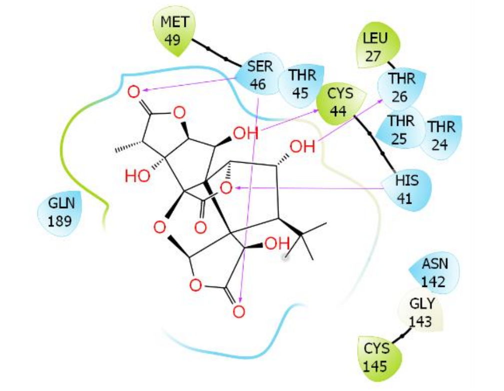
15	**T126**	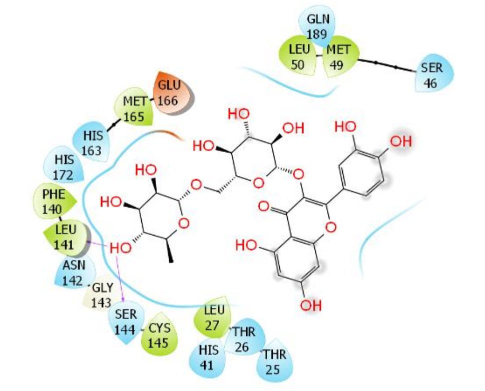	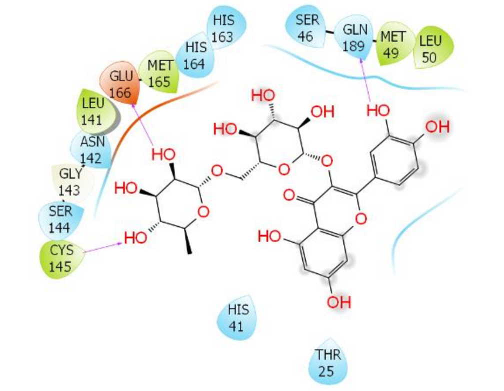
16	**T34**	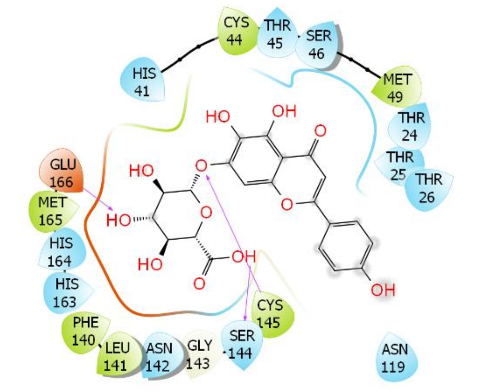	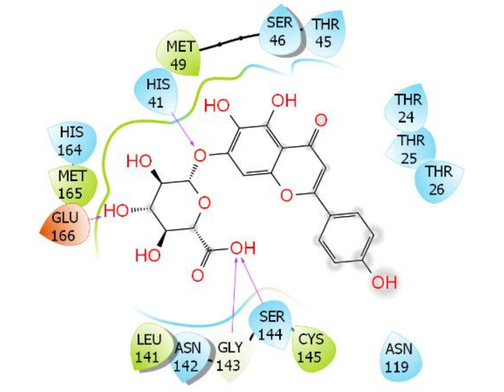

17	**T19**	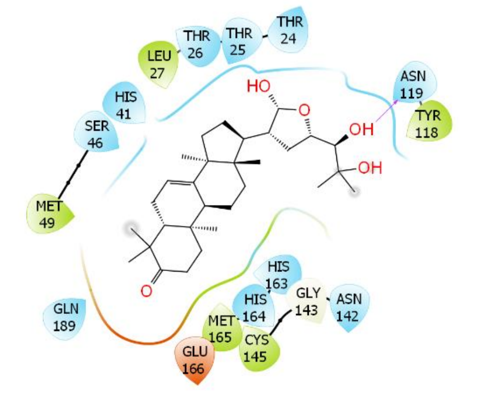	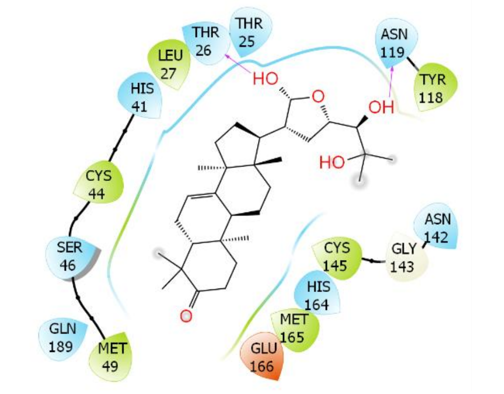
18	**T13**	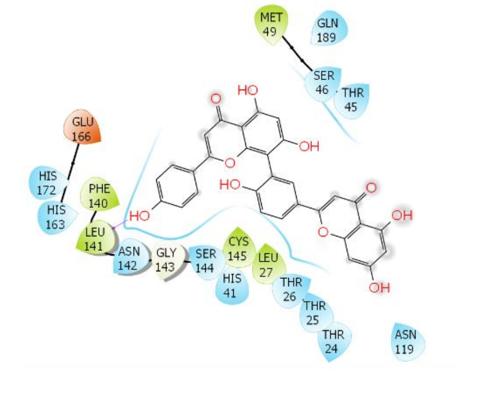	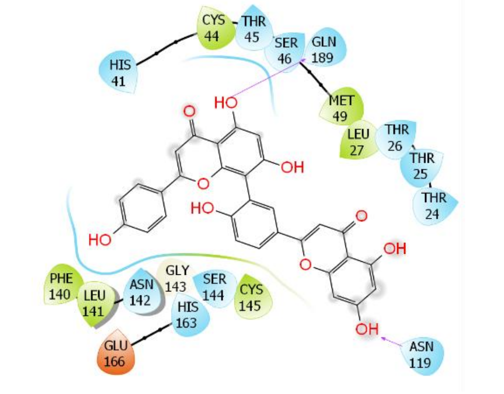
19	**T121**	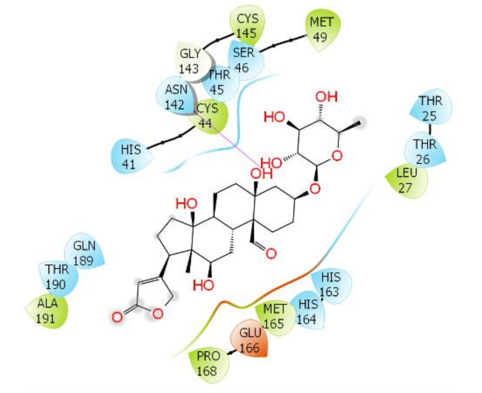	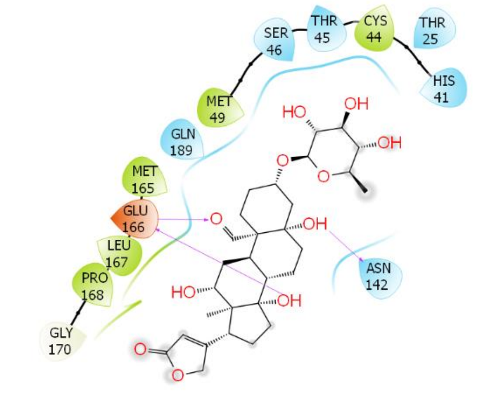
20	**T27**	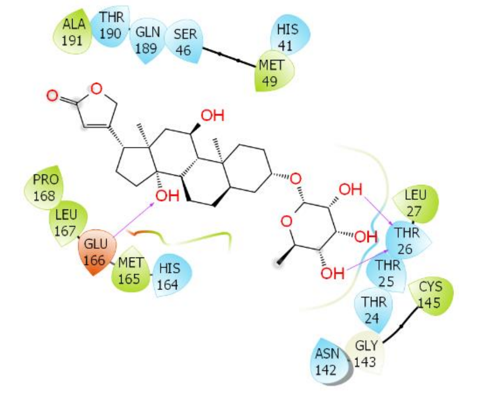	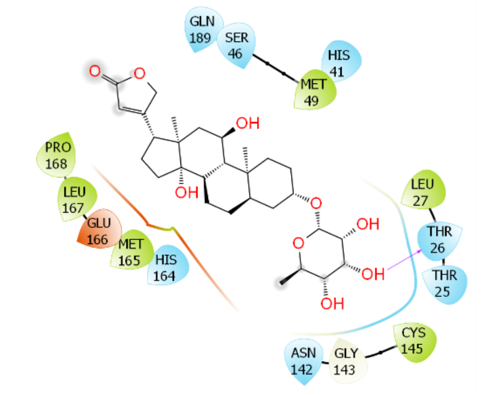

21	**T115**	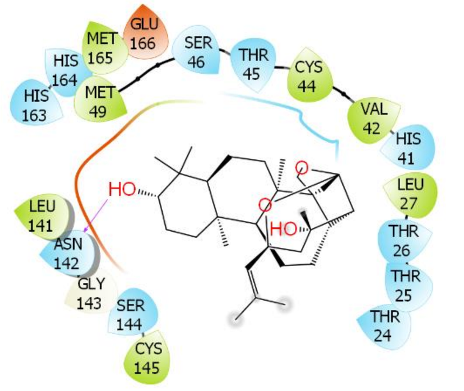	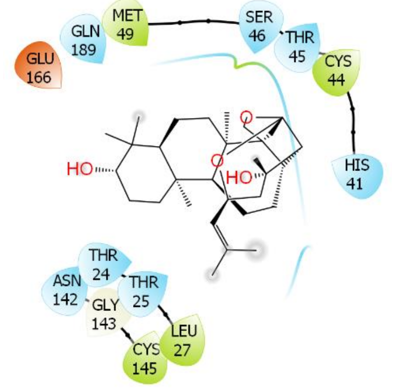
22	**T182**	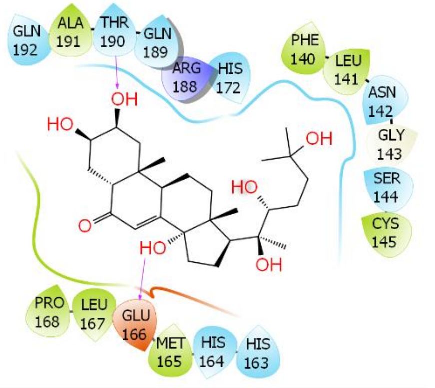	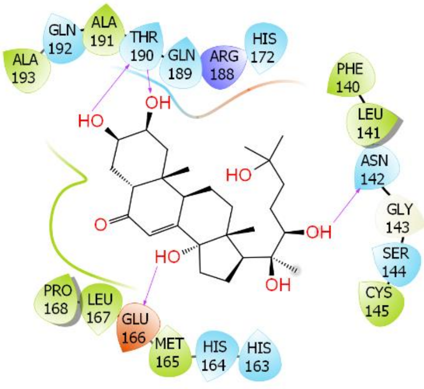
23	**T14**	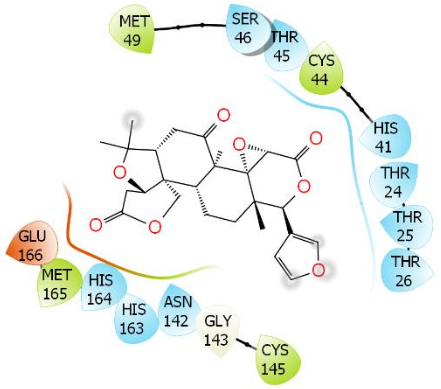	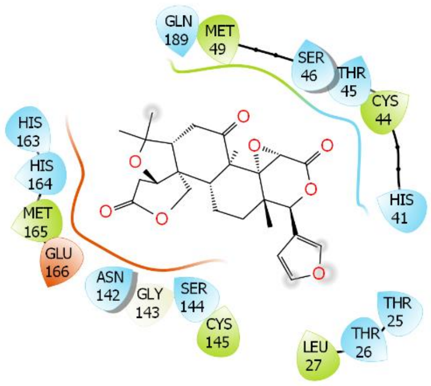
24	**W22**	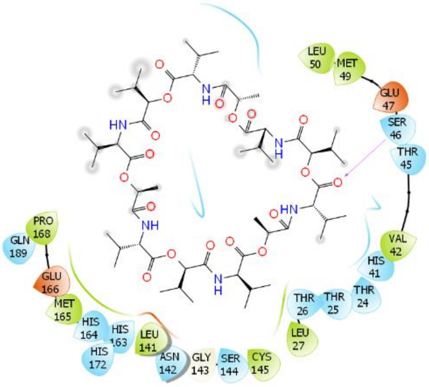	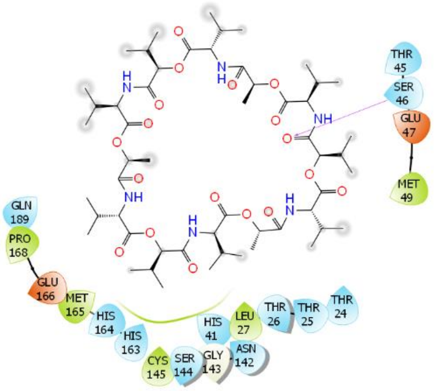

25	**T179**	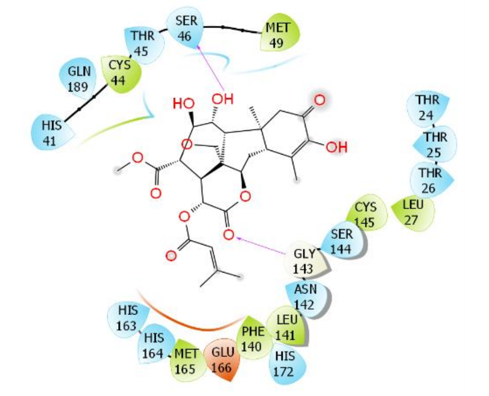	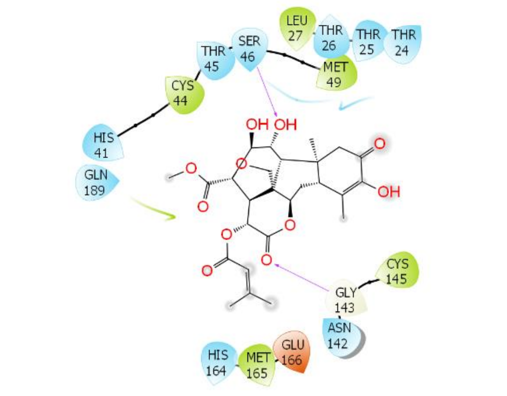
26	**T58**	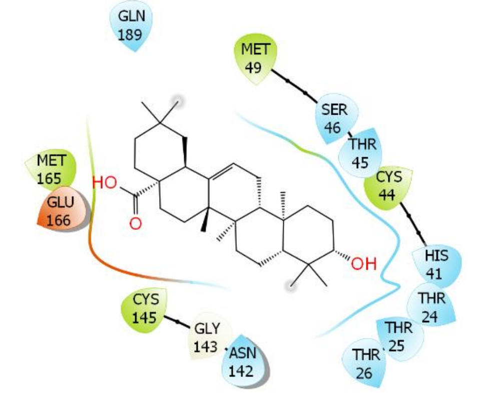	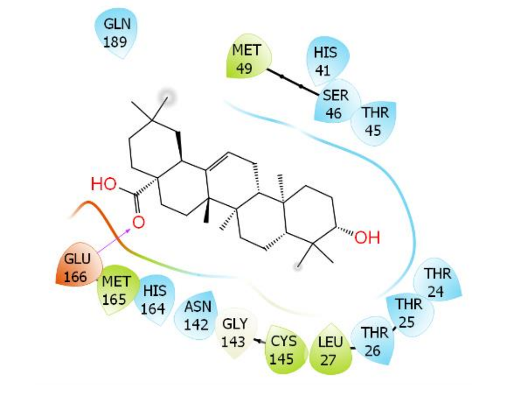
27	**T65**	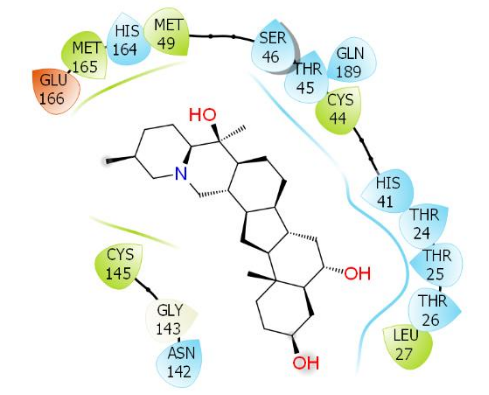	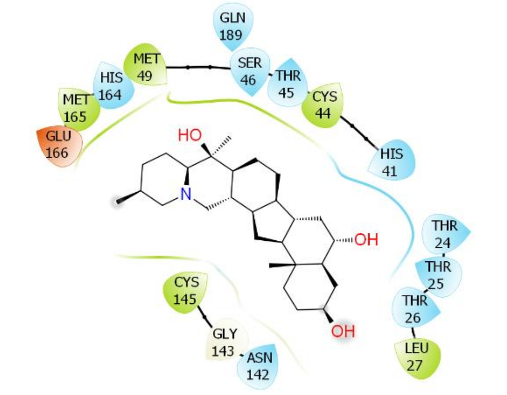
28	**T35**	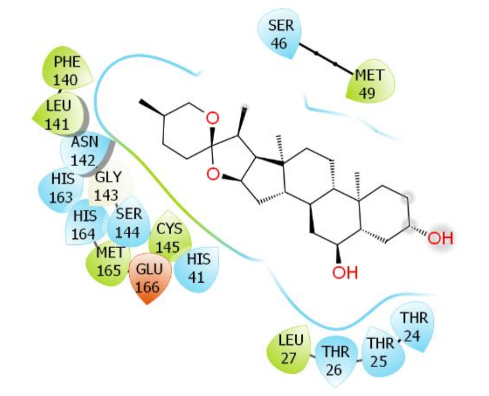	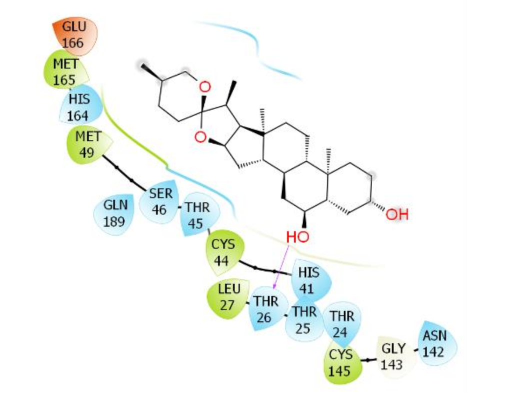

29	**T119**	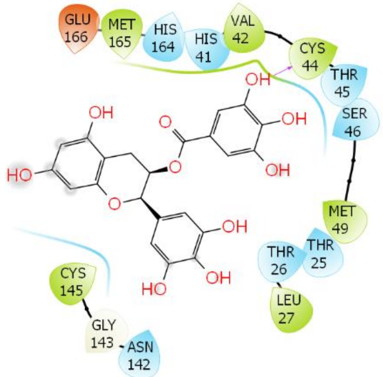	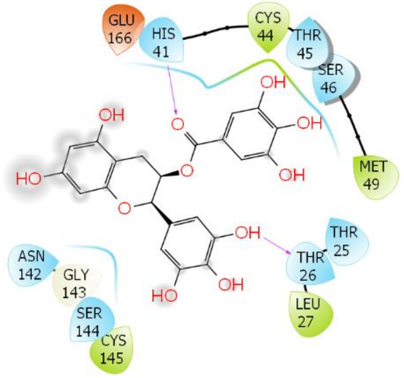
30	**T23**	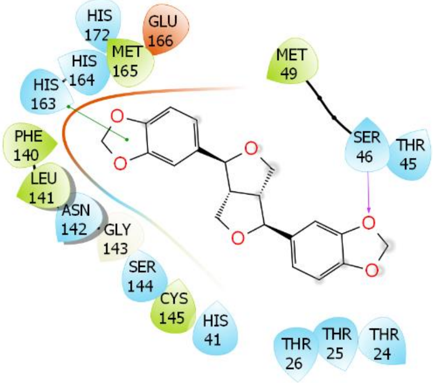	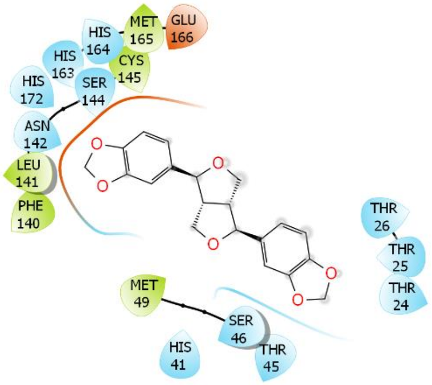
31	**T107**	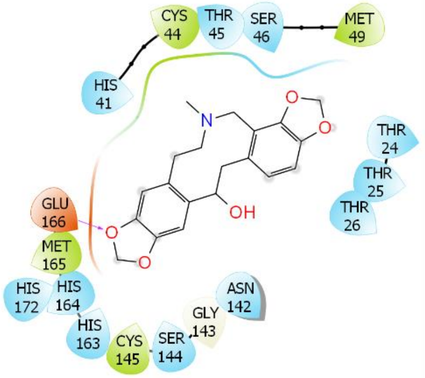	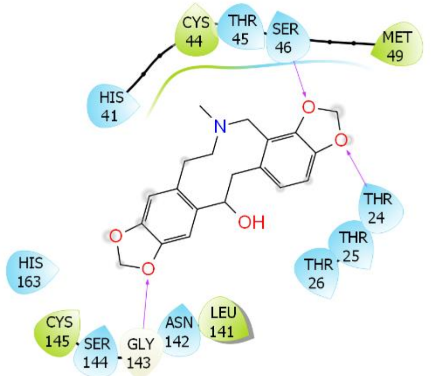
32	**T20**	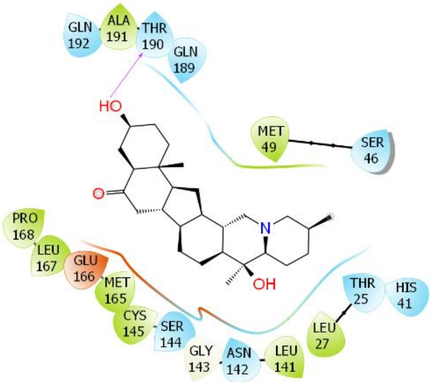	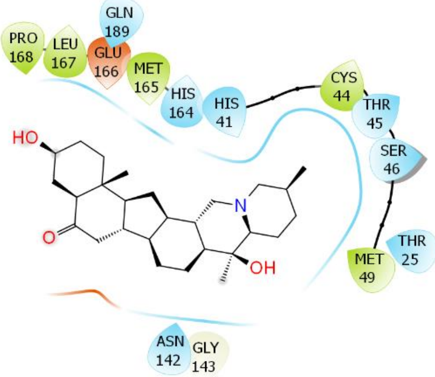

33	**T7**	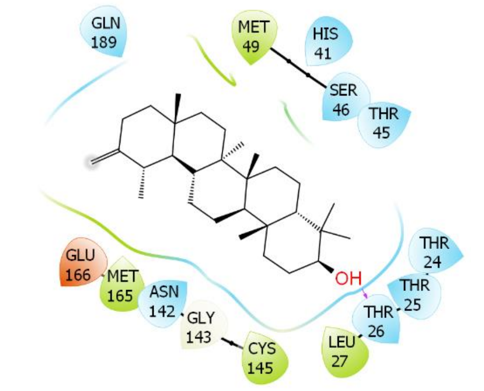	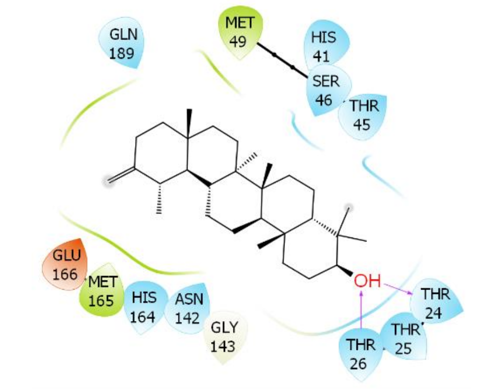
34	**T50**	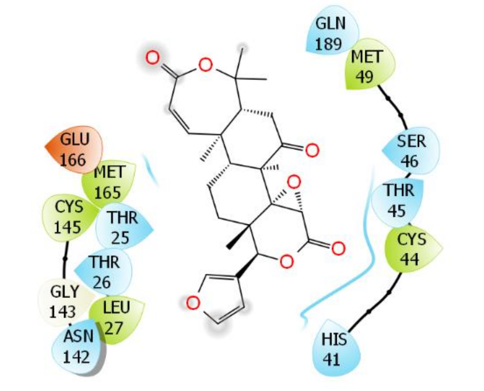	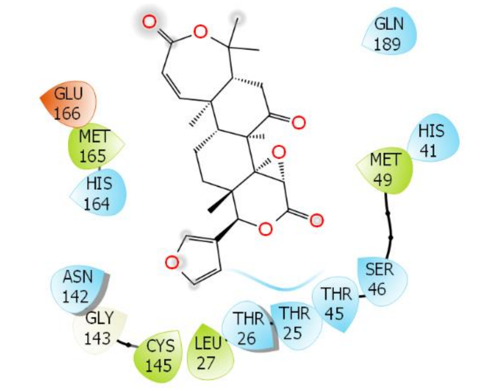
35	**T180**	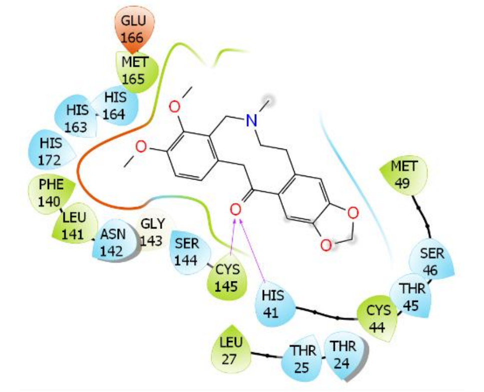	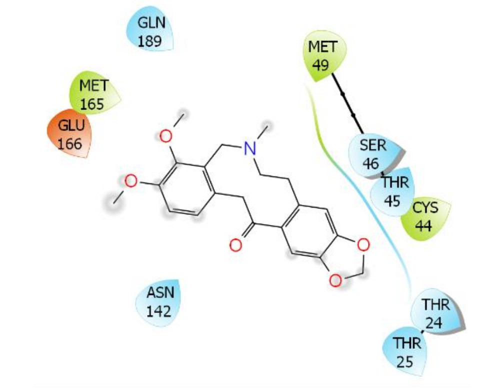
36	**T30**	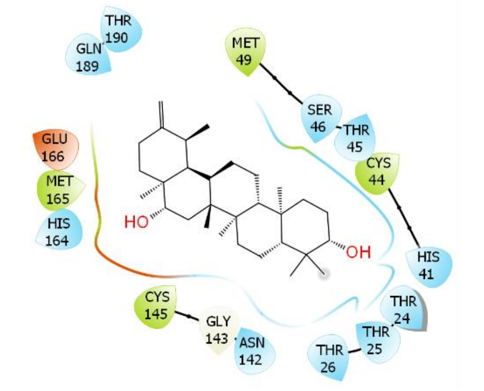	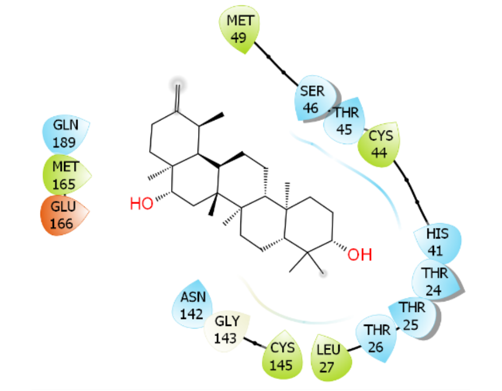

37	**T4**	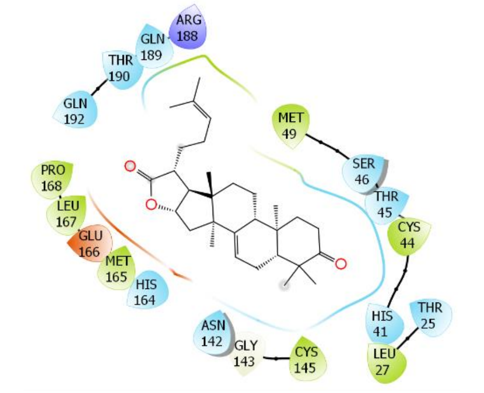	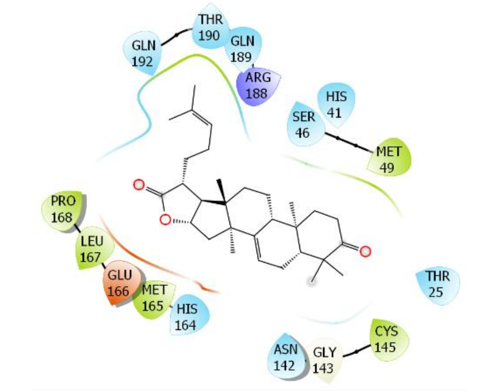
38	**T8**	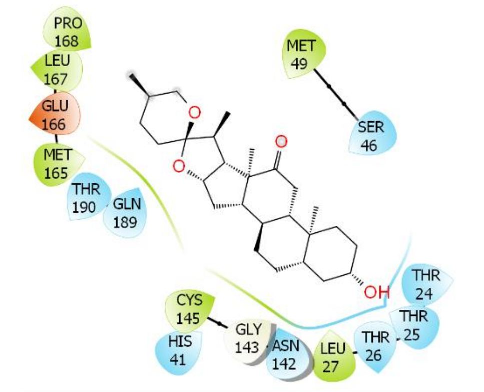	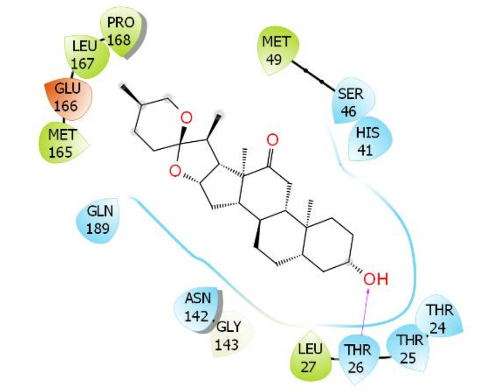
39	**T102**	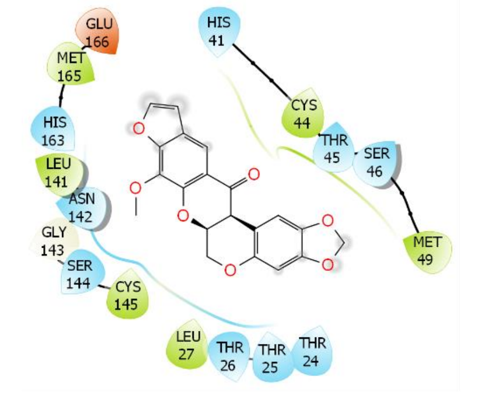	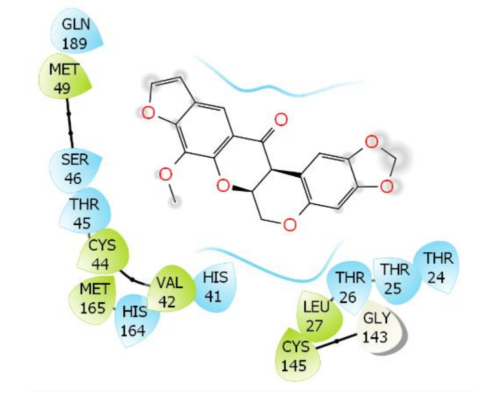
40	**T1**	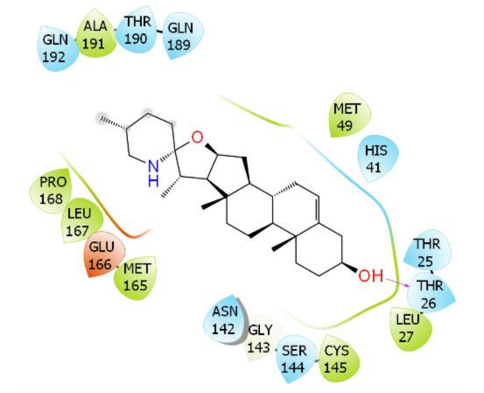	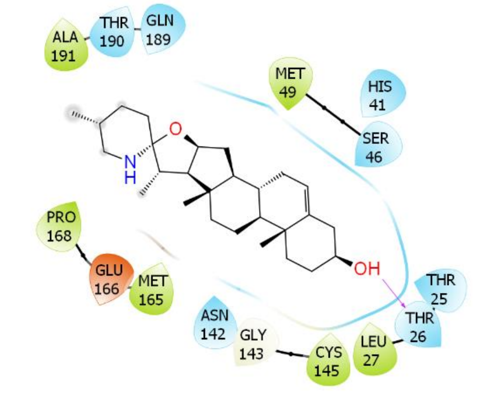

41	**T11**	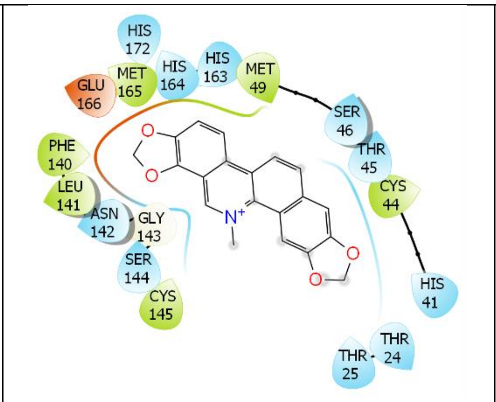HIS 172<br>GLU 166<br>MET 165<br>HIS 164<br>HIS 163<br>MET 49<br>SER 46<br>THR 45<br>CYS 44<br>HIS 41<br>PHE 140<br>LEU 141<br>ASN 142<br>GLY 143<br>SER 144<br>CYS 145<br>THR 25<br>THR 24<br>N+<br>O	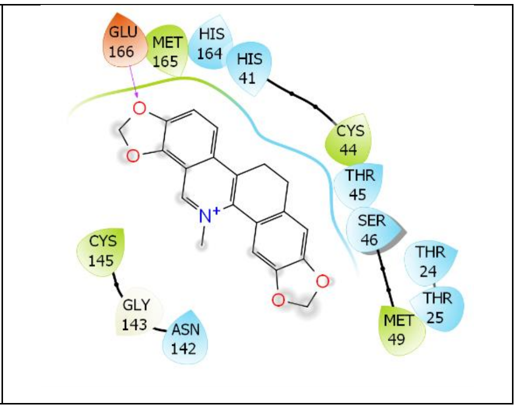GLU 166<br>MET 165<br>HIS 164<br>HIS 41<br>CYS 44<br>THR 45<br>SER 46<br>THR 24<br>THR 25<br>MET 49<br>CYS 145<br>GLY 143<br>ASN 142<br>N+<br>O

Table S3. The pulling force in displacement dependence over FPL simulations

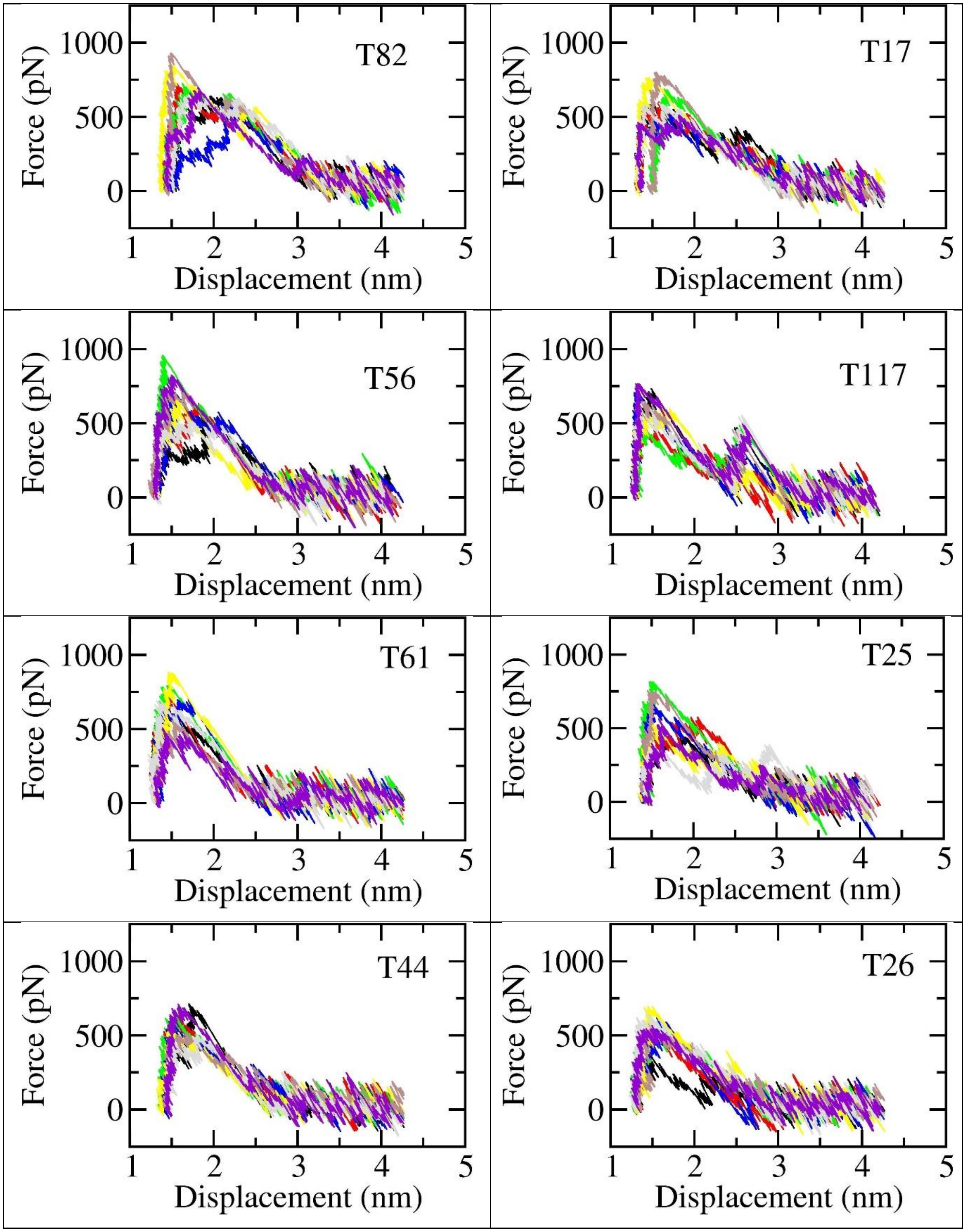

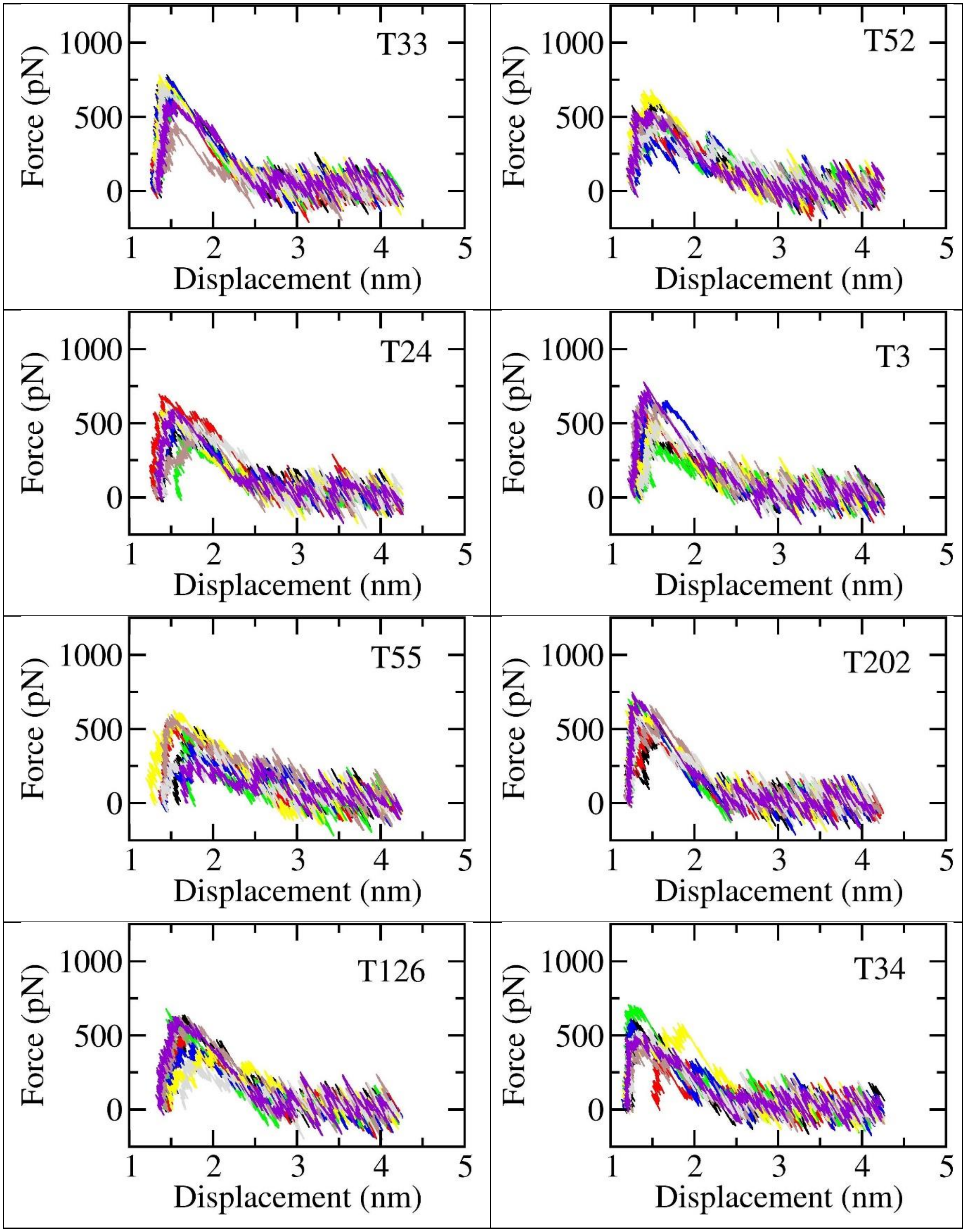

T33
T52
T24
T3
T55
T202
T126
T34
Force (pN)
Displacement (nm)

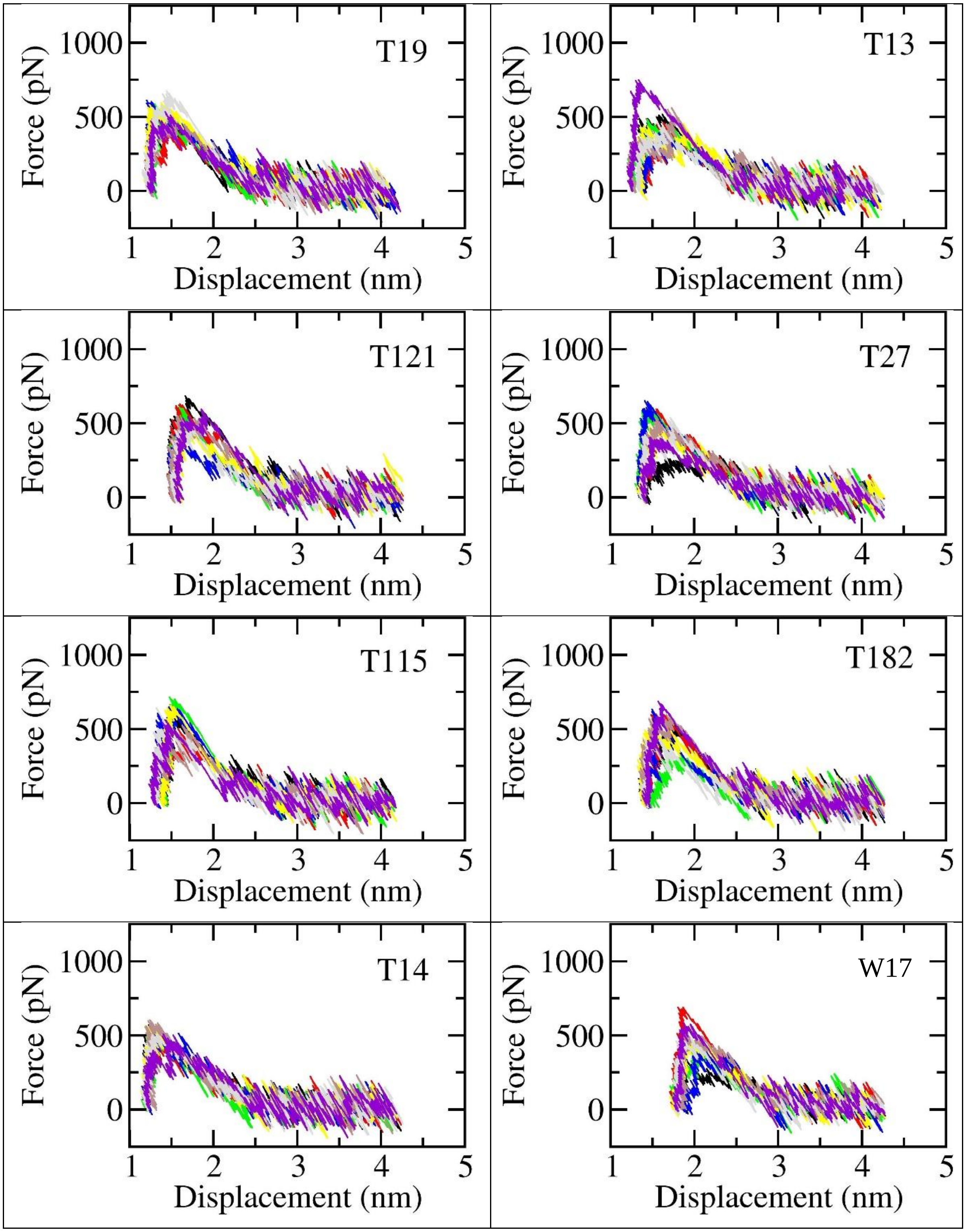
T19
T13
T121
T27
T115
T182
T14
W17
Force (pN)
Displacement (nm)
1000
500
0
1
2
3
4
5

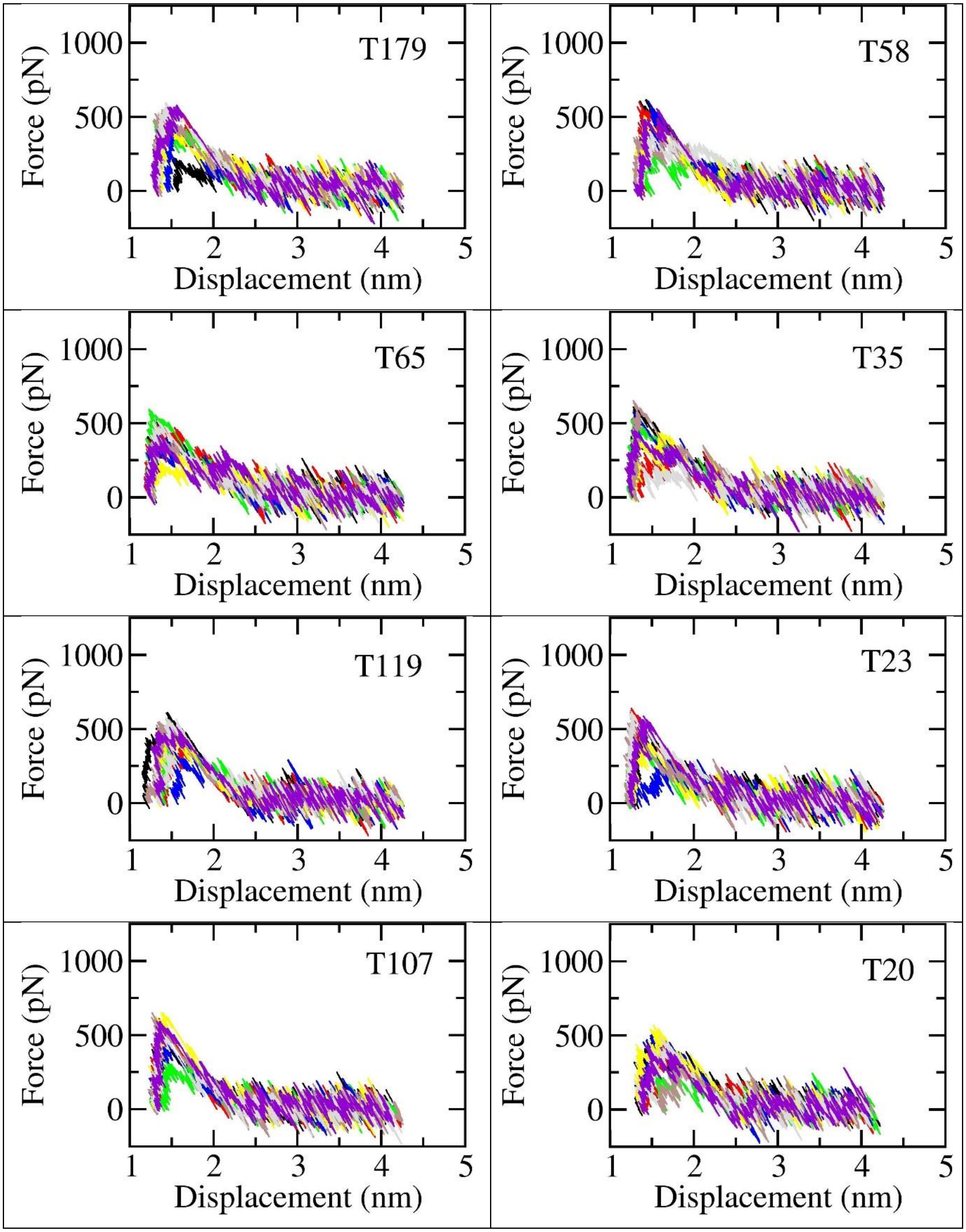
T179
T58
T65
T35
T119
T23
T107
T20
Force (pN)
Displacement (nm)
1000
500
0
1
2
3
4
5

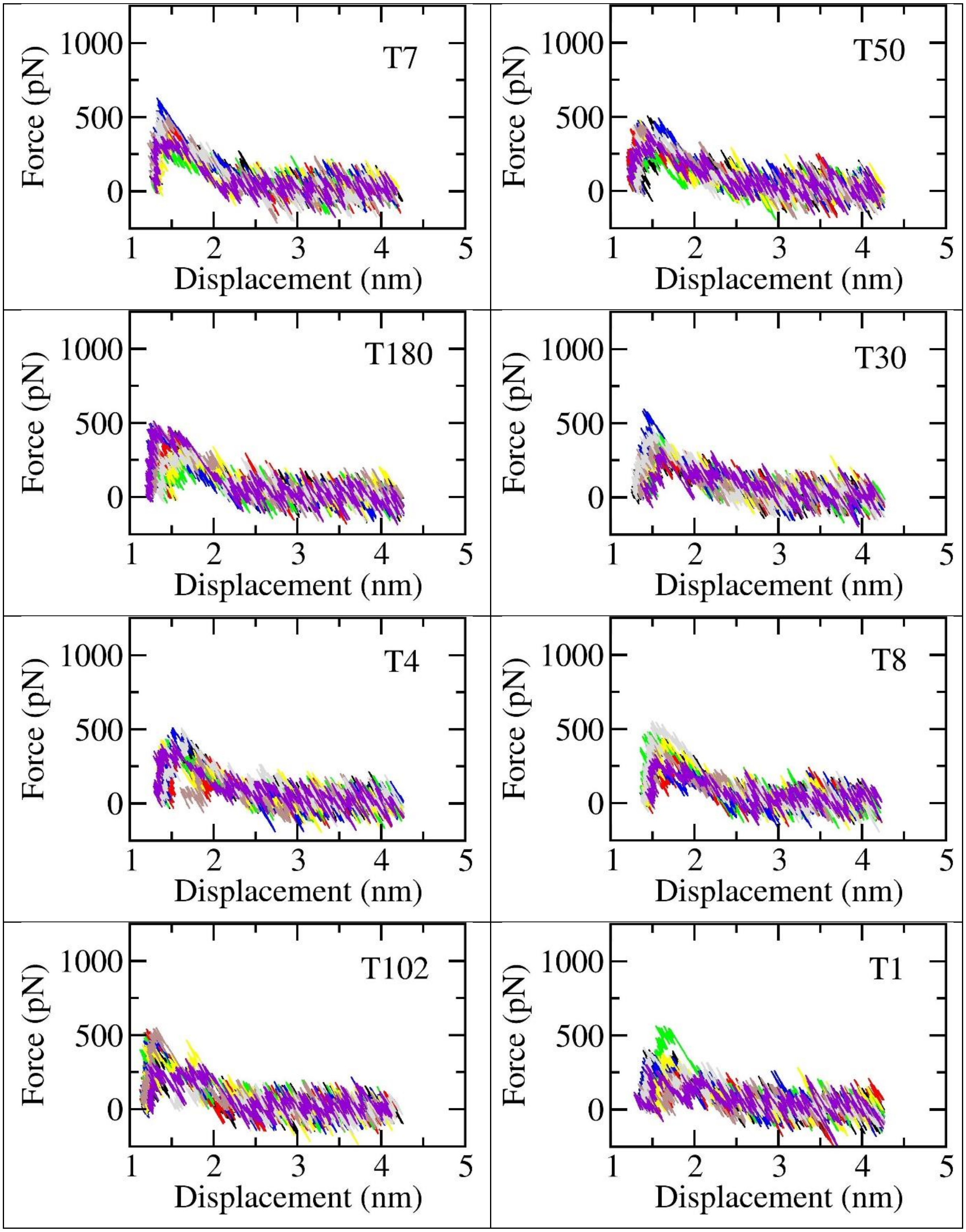
T7
T50
T180
T30
T4
T8
T102
T1
Force (pN)
Displacement (nm)
1000
500
0
1
2
3
4
5

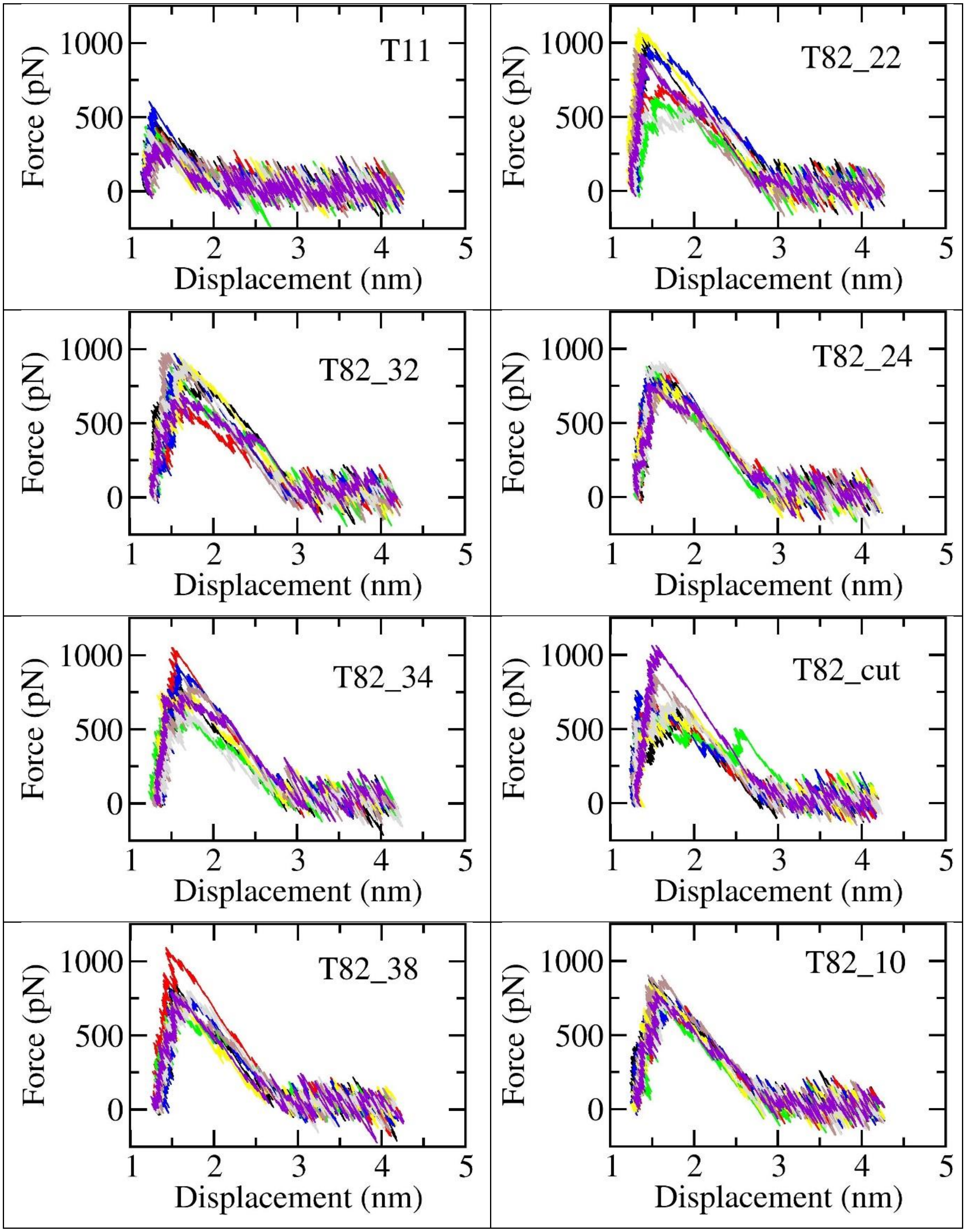
T11
T82_22
T82_32
T82_24
T82_34
T82_cut
T82_38
T82_10
Force (pN)
Displacement (nm)
1000
500
0
1
2
3
4
5

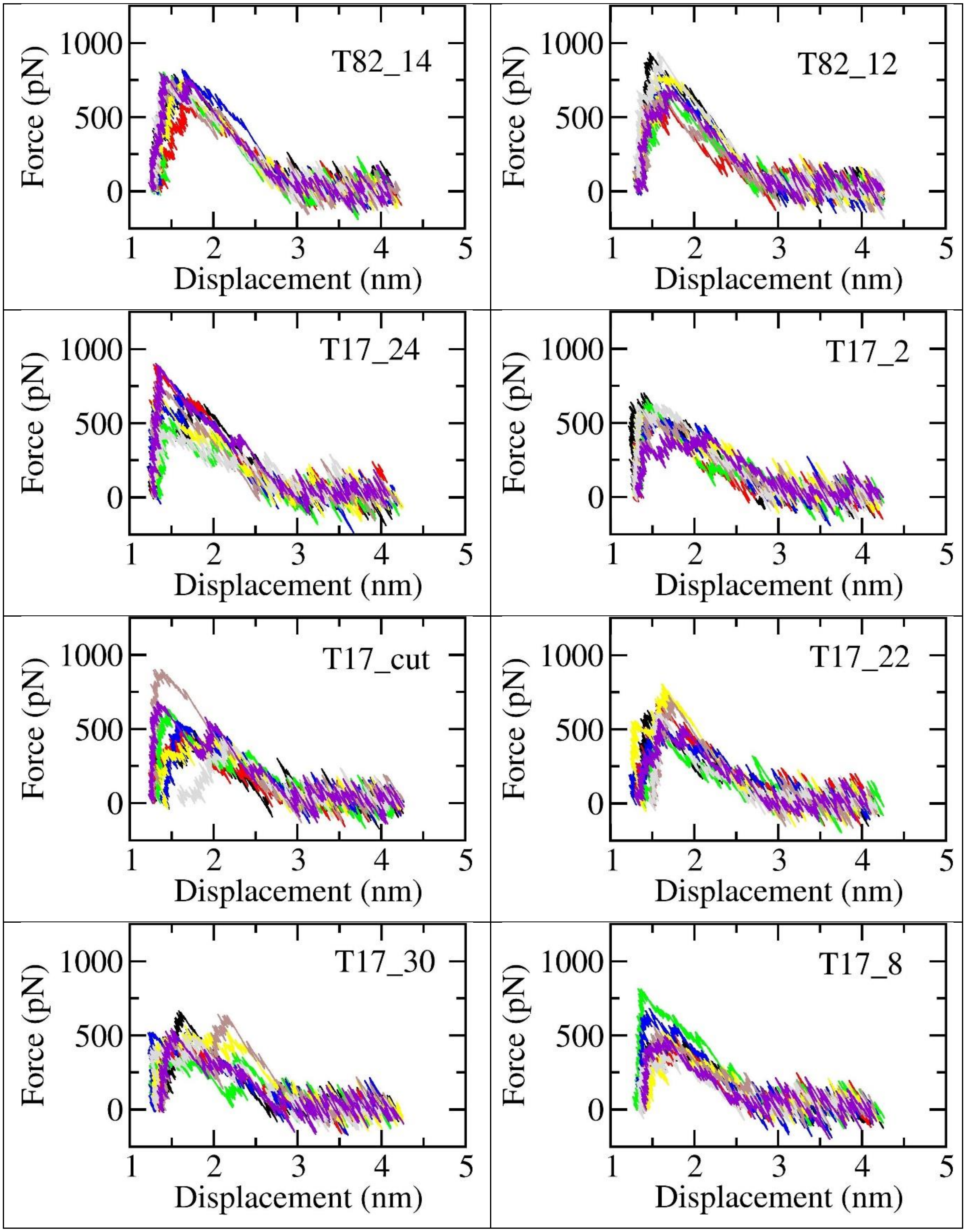
T82_14
T82_12
T17_24
T17_2
T17_cut
T17_22
T17_30
T17_8
Force (pN)
Displacement (nm)

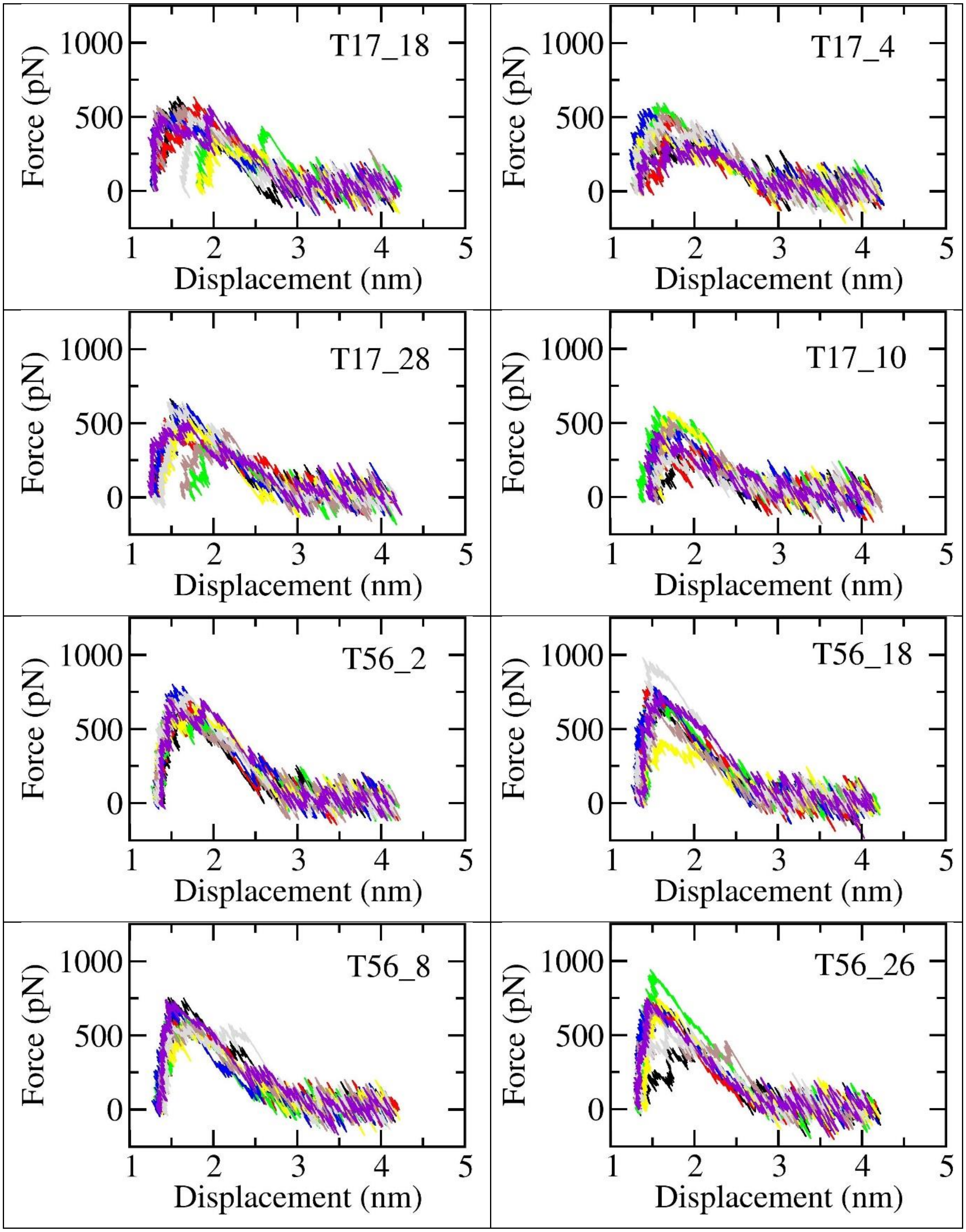
T17_18
T17_4
T17_28
T17_10
T56_2
T56_18
T56_8
T56_26
Force (pN)
Displacement (nm)
0
500
1000
1
2
3
4
5

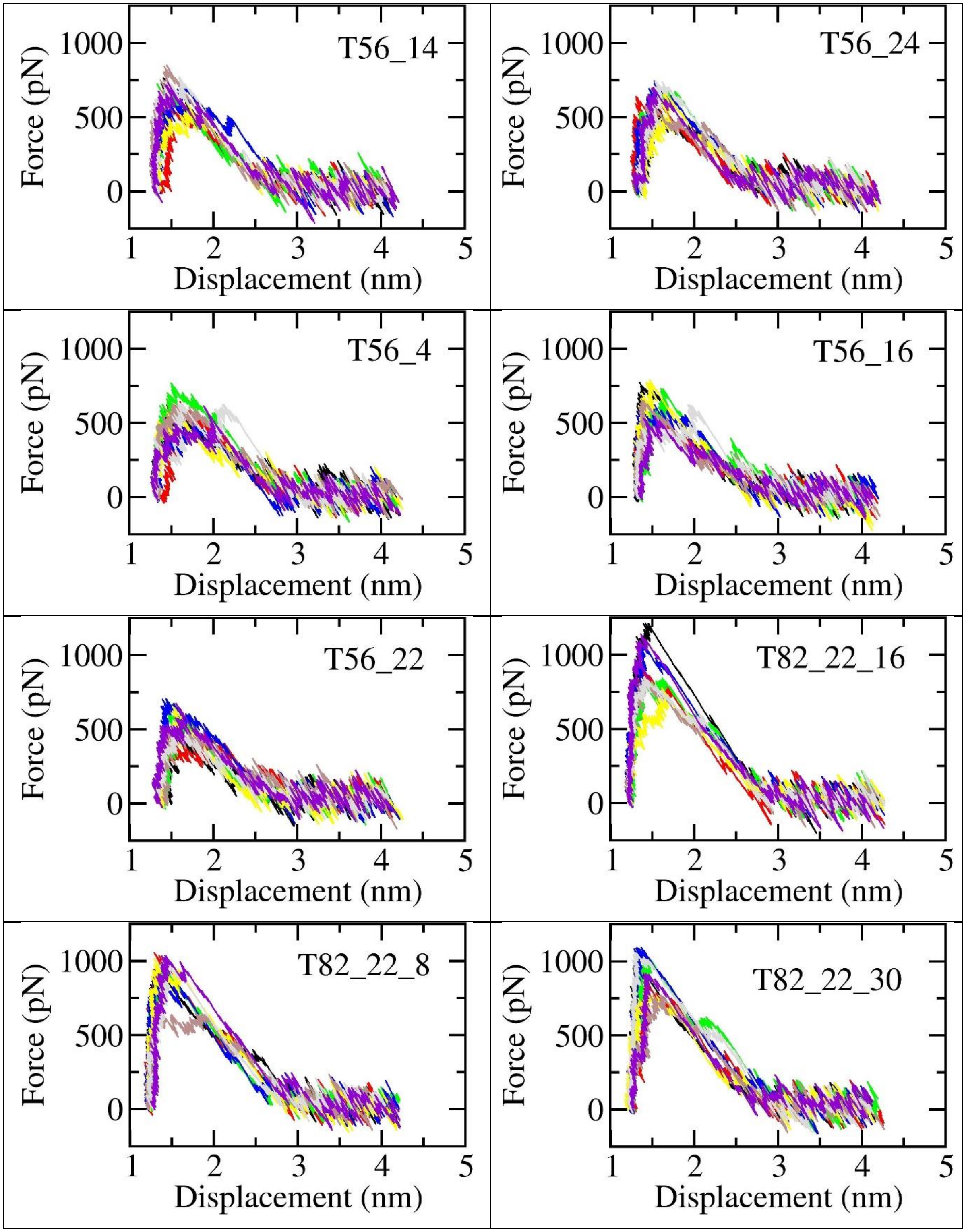

T56_14
T56_24
T56_4
T56_16
T56_22
T82_22_16
T82_22_8
T82_22_30
Force (pN)
Displacement (nm)
1000
500
0
1
2
3
4
5

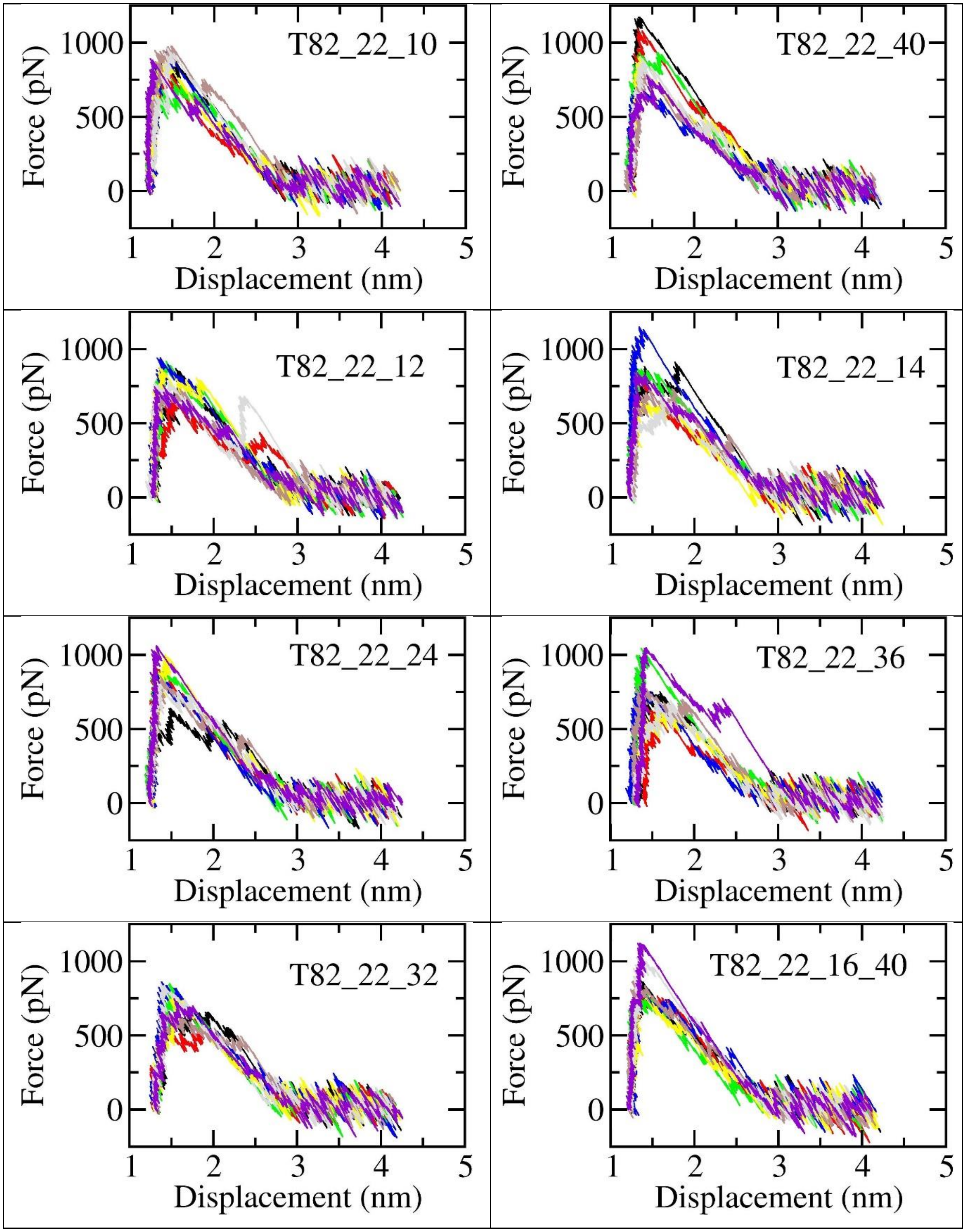

T82_22_10
T82_22_40
T82_22_12
T82_22_14
T82_22_24
T82_22_36
T82_22_32
T82_22_16_40
Force (pN)
Displacement (nm)

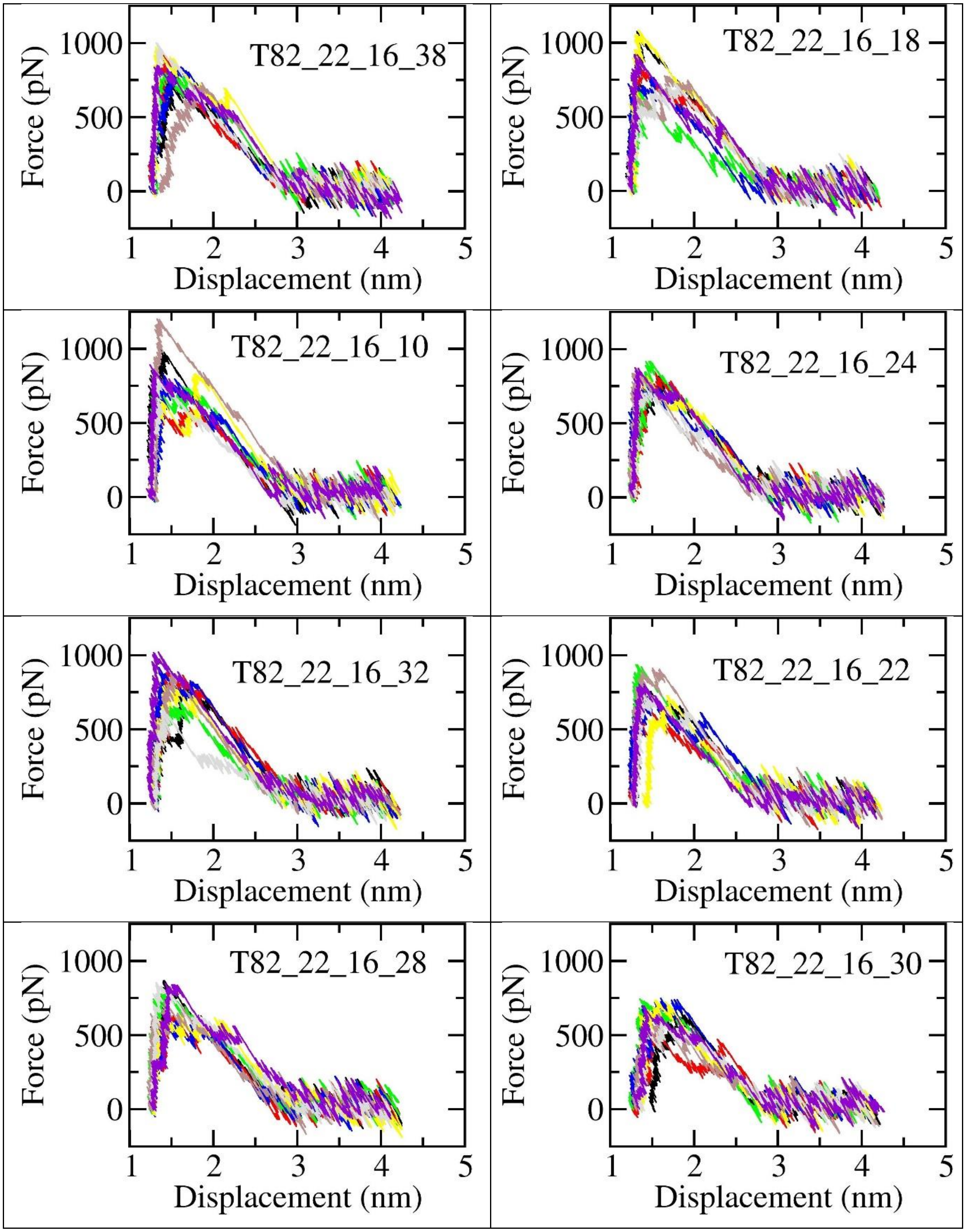
T82_22_16_38
T82_22_16_18
T82_22_16_10
T82_22_16_24
T82_22_16_32
T82_22_16_22
T82_22_16_28
T82_22_16_30
Force (pN)
Displacement (nm)

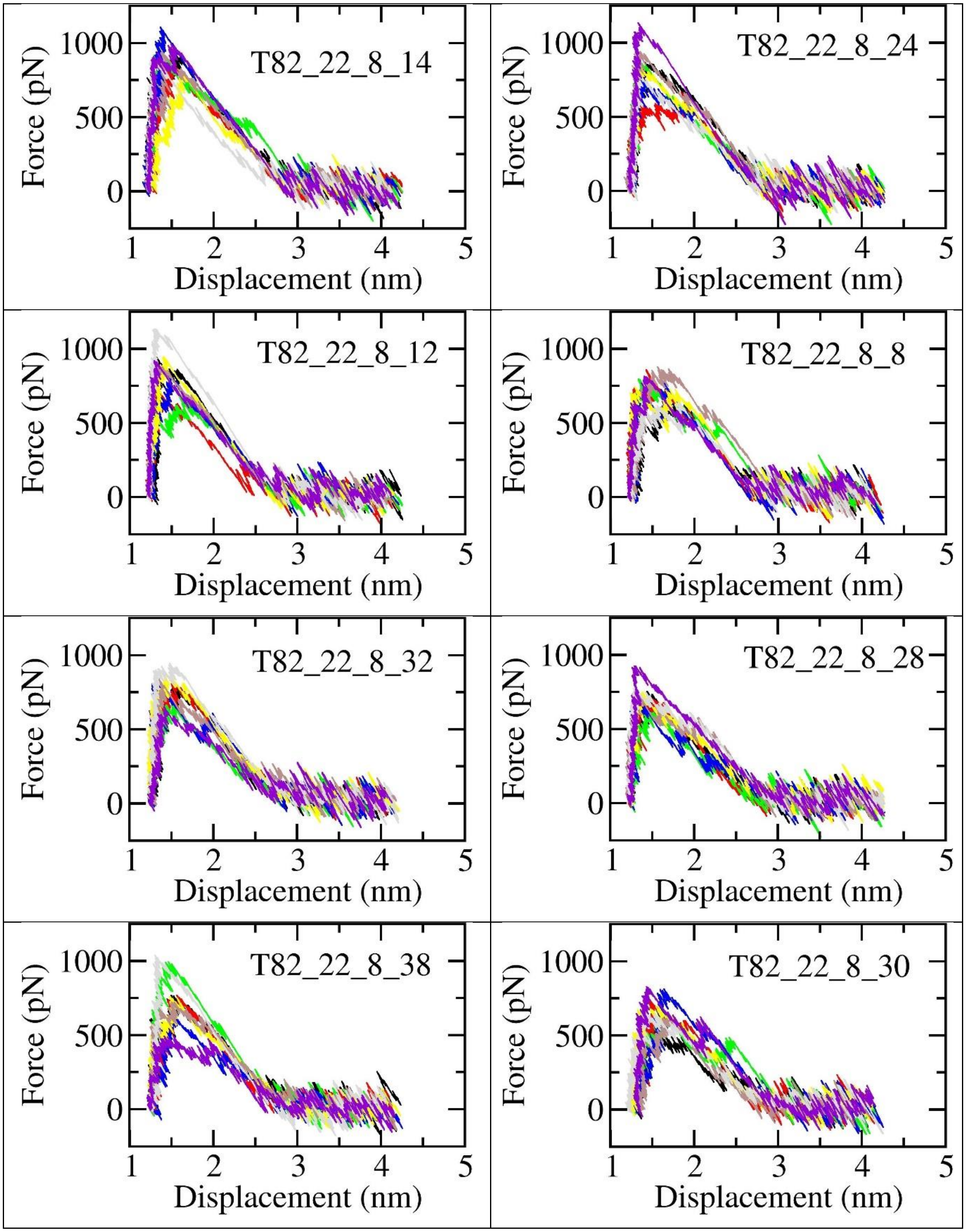
T82_22_8_14
T82_22_8_24
T82_22_8_12
T82_22_8_8
T82_22_8_32
T82_22_8_28
T82_22_8_38
T82_22_8_30
Force (pN)
0
500
1000
Displacement (nm)
1
2
3
4
5

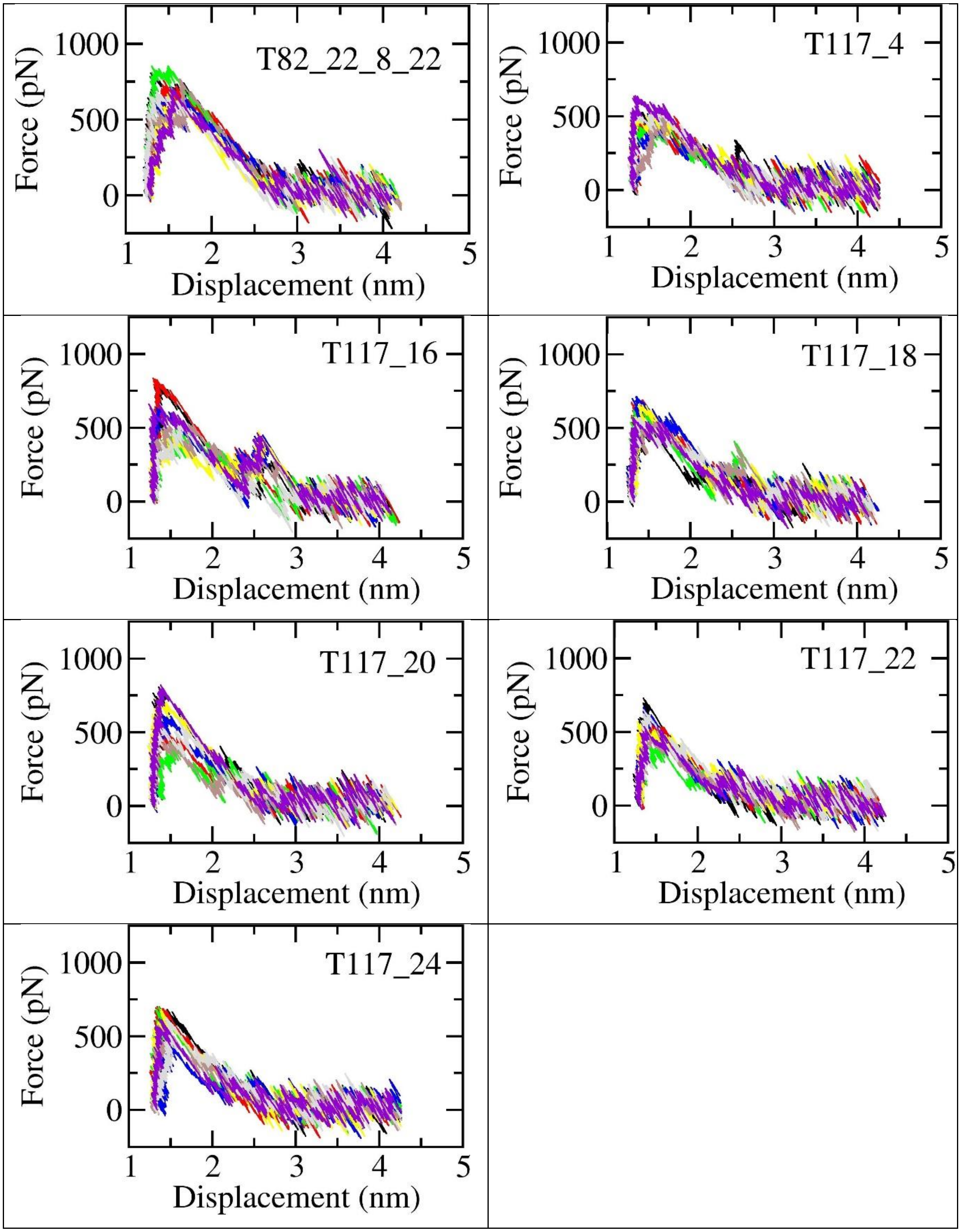

T82_22_8_22
T117_4
T117_16
T117_18
T117_20
T117_22
T117_24
Force (pN)
Displacement (nm)
1000
500
0
1
2
3
4
5

Table S4. MD-refined Structures of SARS-CoV-2 Mpro + Modified Compounds.

N^0	Compound	MD-refined structure
**1**	**T82_22**	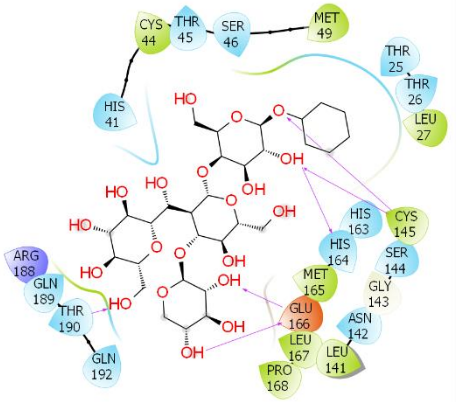
**2**	**T82_32**	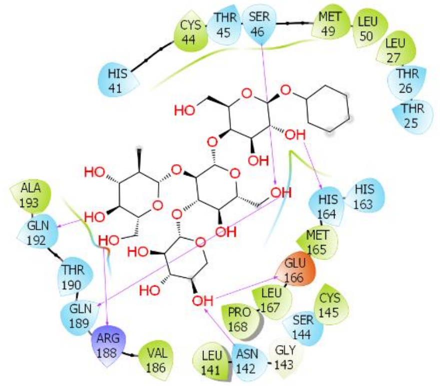
**3**	**T82_24**	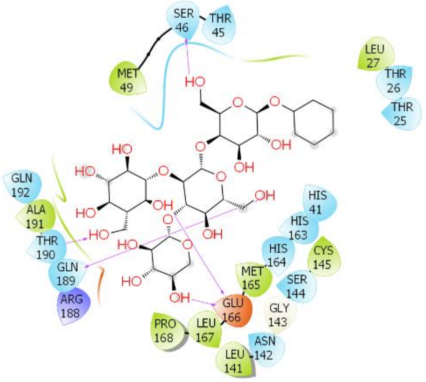
**4**	**T82_34**	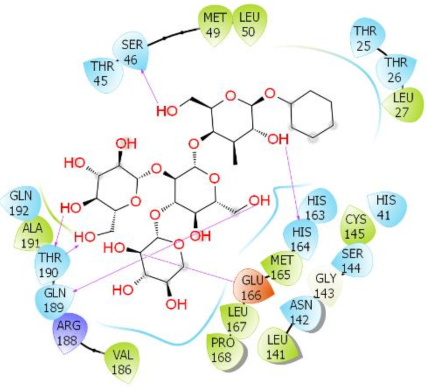

**5**	**T82_cut**	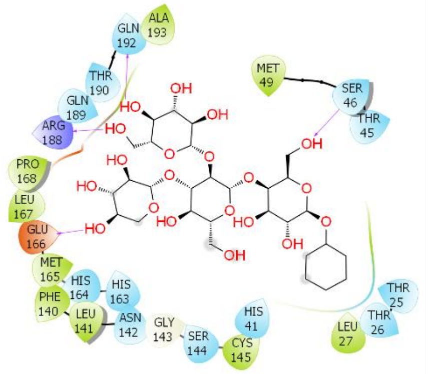
**6**	**T82_38**	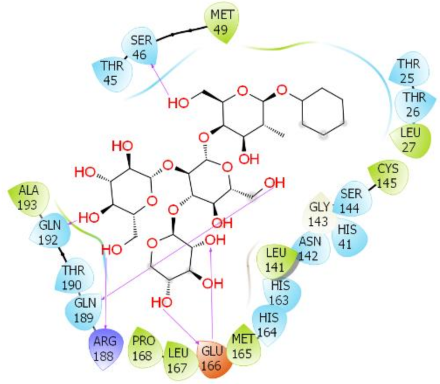
**7**	**T82_10**	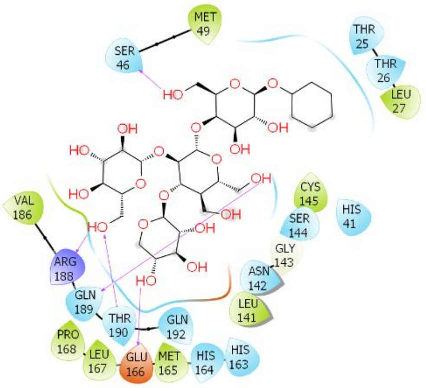
**8**	**T82_14**	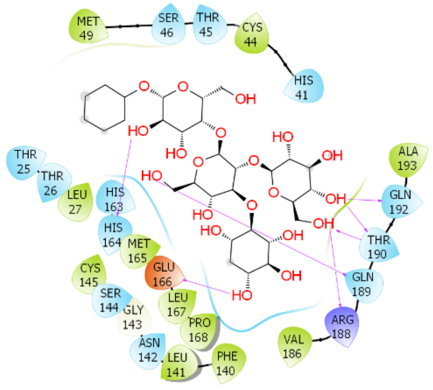

**9**	**T82_12**	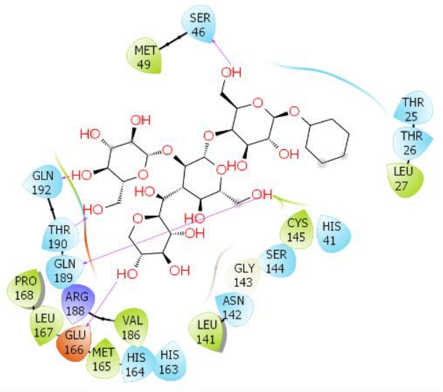
**10**	**T17_24**	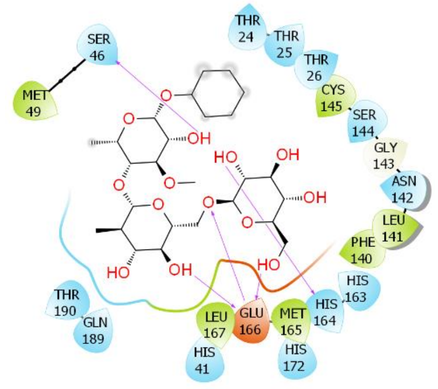
**11**	**T17_2**	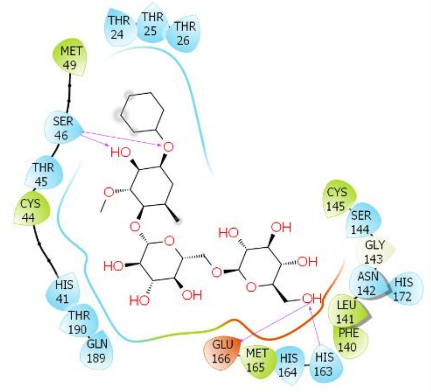
**12**	**T17_cut**	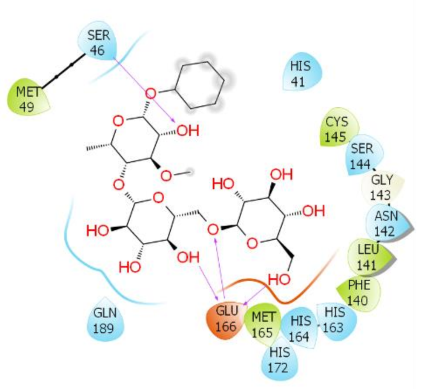

**13**	**T17_22**	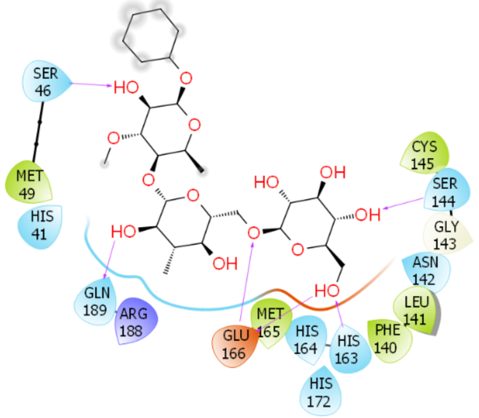
**14**	**T17_30**	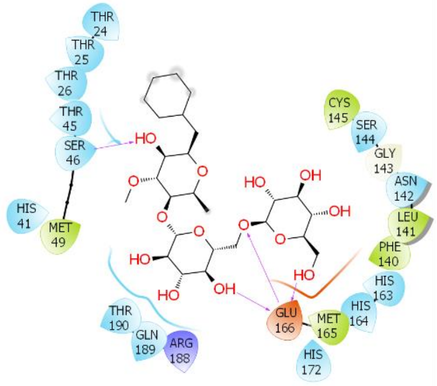
**15**	**T17_8**	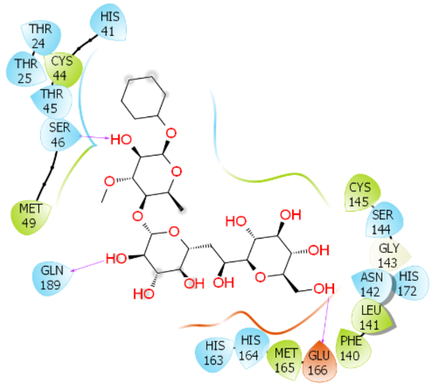
**16**	**T17_18**	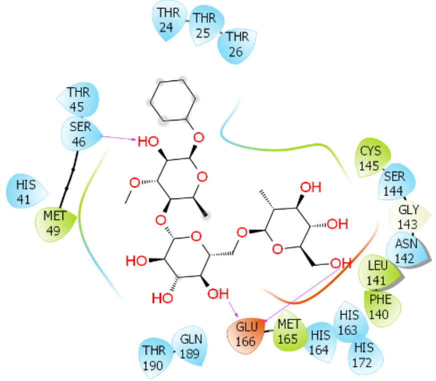

**17**	**T17_4**	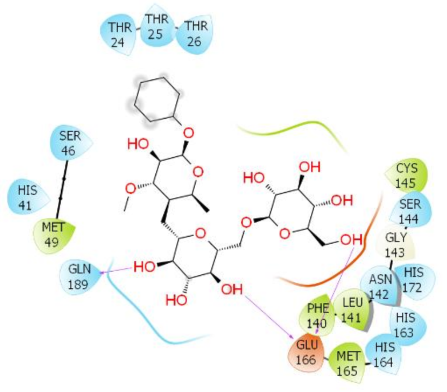
**18**	**T17_28**	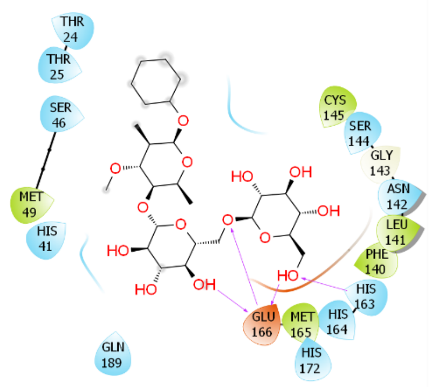
**19**	**T17_10**	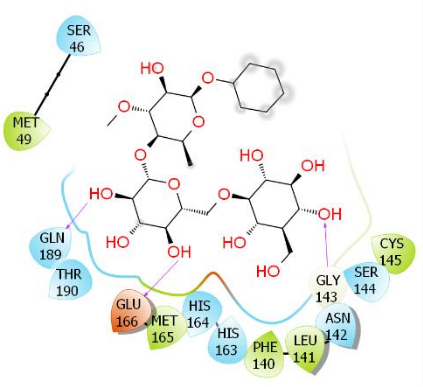
**20**	**T56_2**	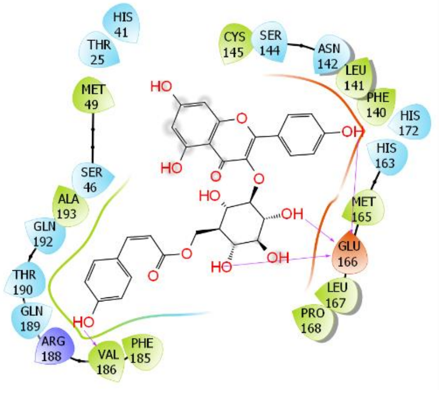

**21**	**T56_18**	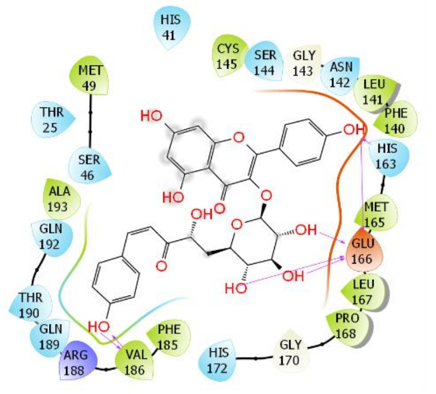
**22**	**T56_8**	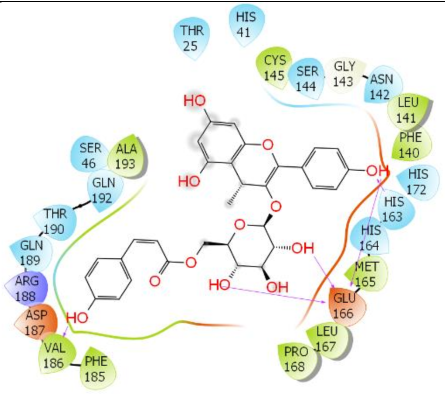
**23**	**T56_26**	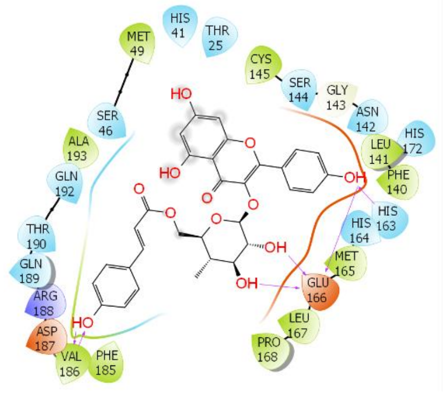
**24**	**T56_14**	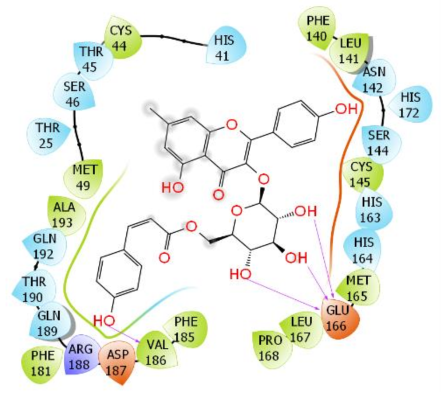

**25**	**T56_24**	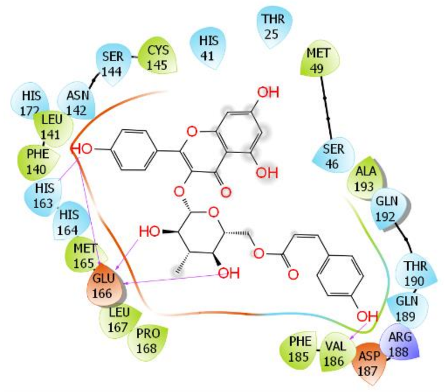
**26**	**T56_4**	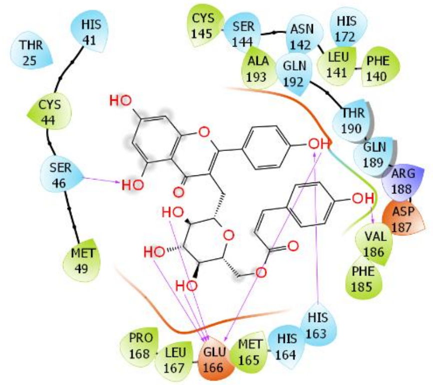
**27**	**T56_16**	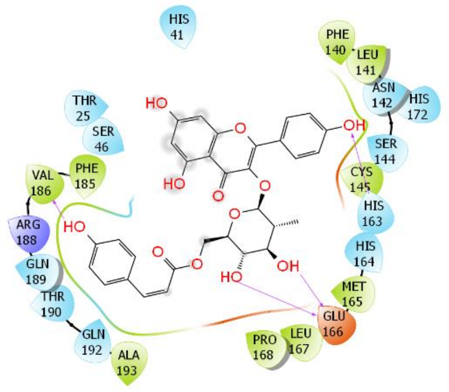
**28**	**T56_22**	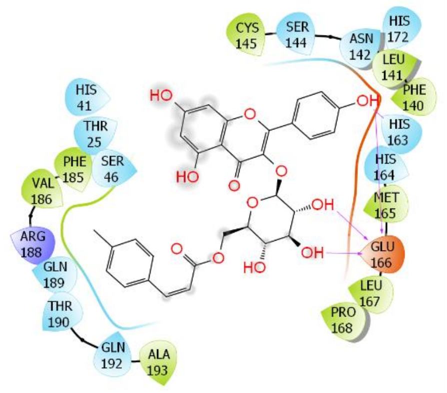

Table S5. MD-refined Structures of SARS-CoV-2 Mpro + Modified Compounds, which were predicted by DeepFrag calculation.

N⁰	Compound	MD-refined structure
**1**	**T82_22_16**	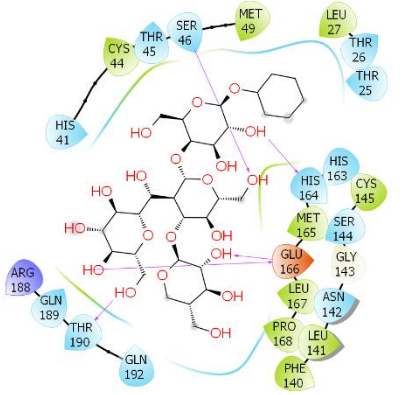
**2**	**T82_22_8**	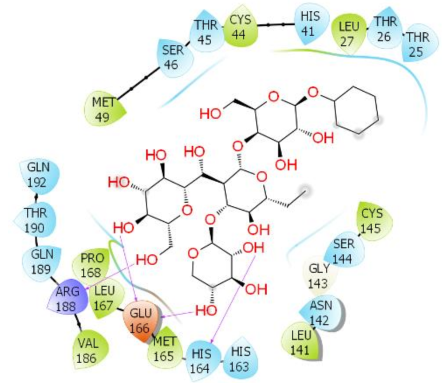
**3**	**T82_22_30**	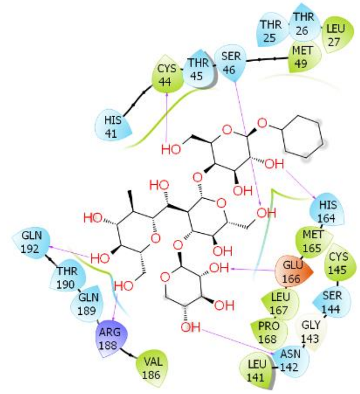
**4**	**T82_22_10**	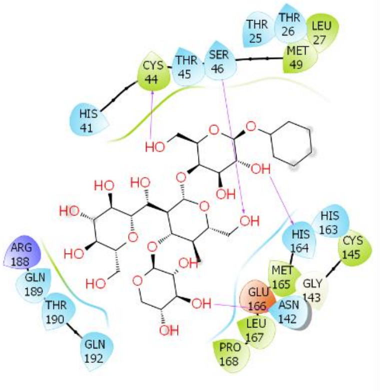

**5**	**T82_22_40**	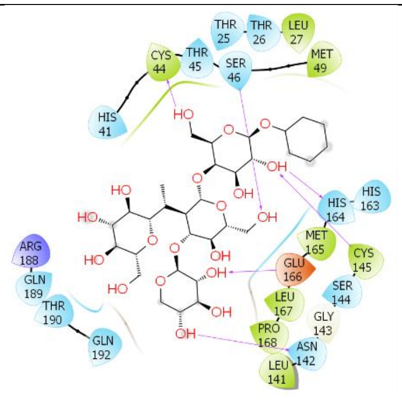
**6**	**T82_22_12**	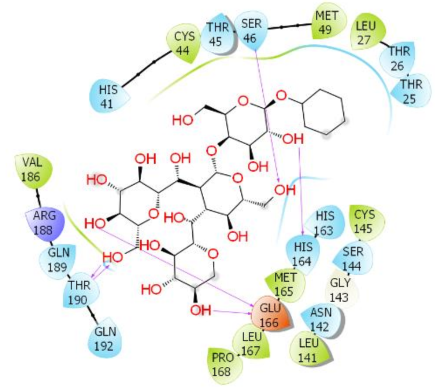
**7**	**T82_22_14**	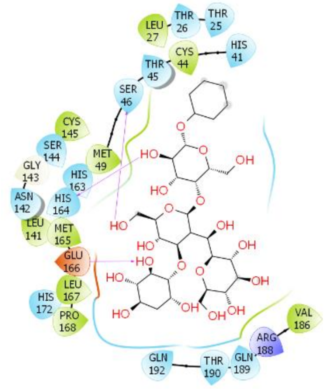
**8**	**T82_22_24**	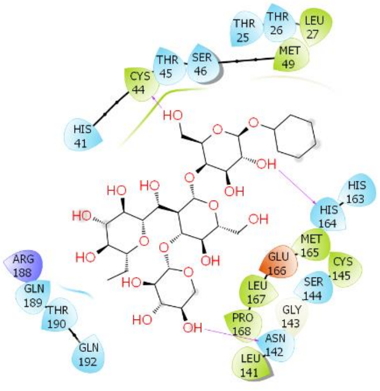

**9**	**T82_22_36**	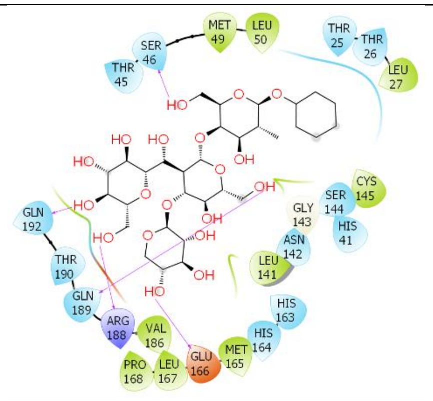
**10**	**T82_22_32**	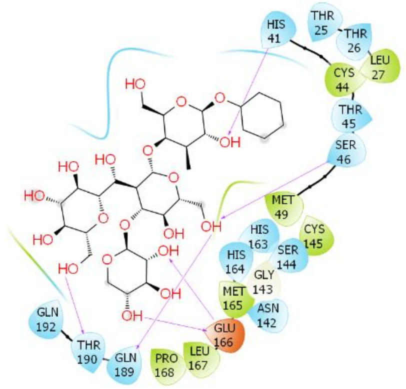

Table S6. MD-refined Structures of SARS-CoV-2 Mpro + Modified Compounds, which were suggested by DeepFrag estimations.

N⁰	Compound	MD-refined structure
**1**	**T82_22_16_40**	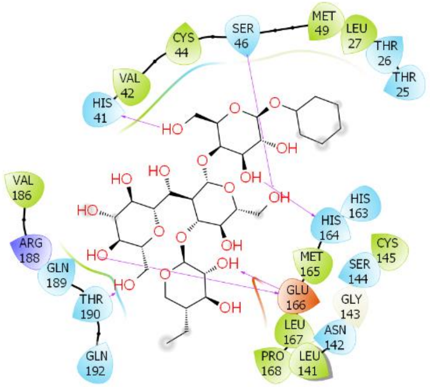
**2**	**T82_22_16_38**	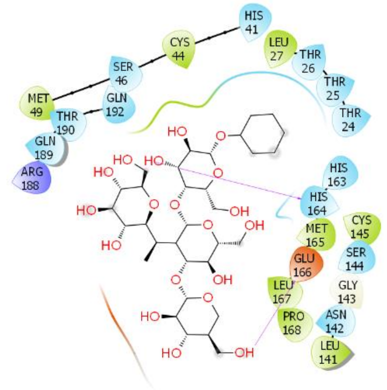
**3**	**T82_22_16_18**	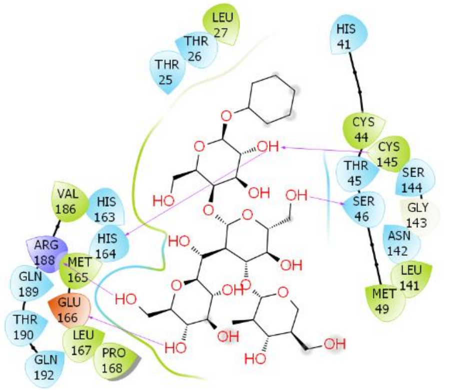
**4**	**T82_22_16_10**	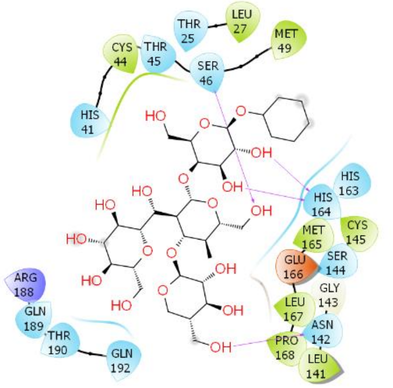

**5**	**T82_22_16_24**	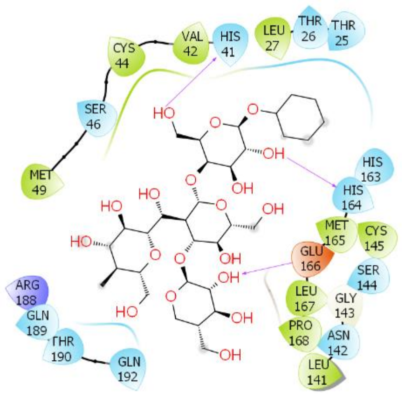
**6**	**T82_22_16_32**	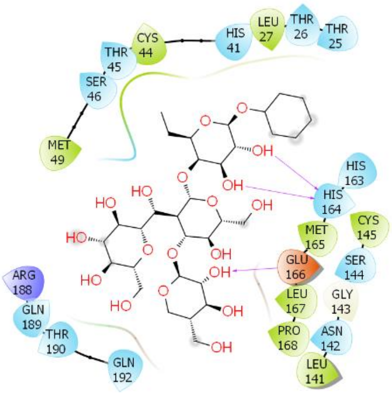
**7**	**T82_22_16_22**	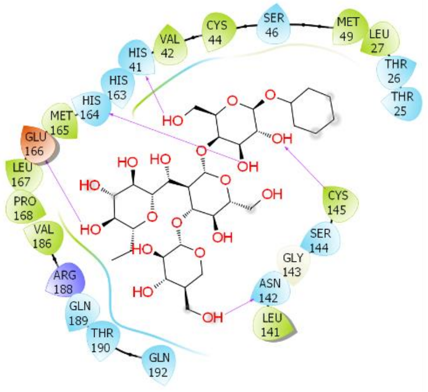
**8**	**T82_22_16_28**	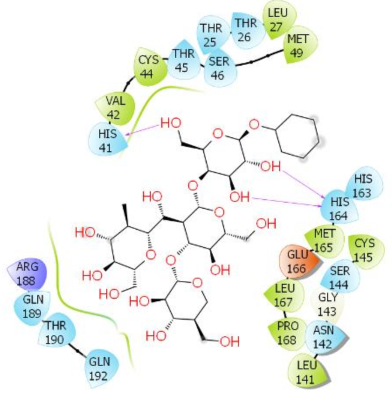

**9**	**T82_22_16_30**	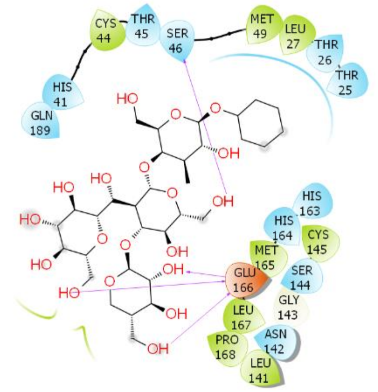
**10**	**T82_22_8_14**	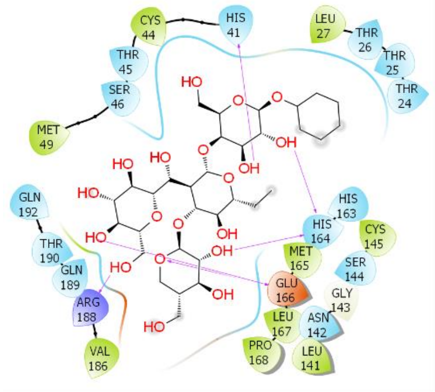
**11**	**T82_22_8_24**	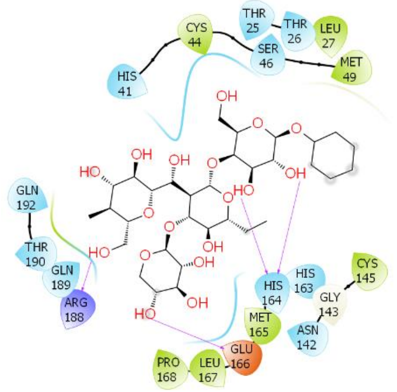
**12**	**T82_22_8_12**	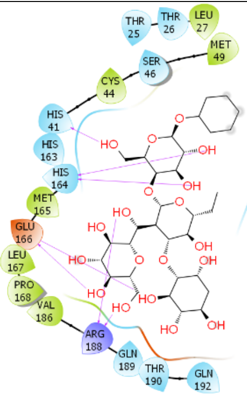

**13**	**T82_22_8_8**	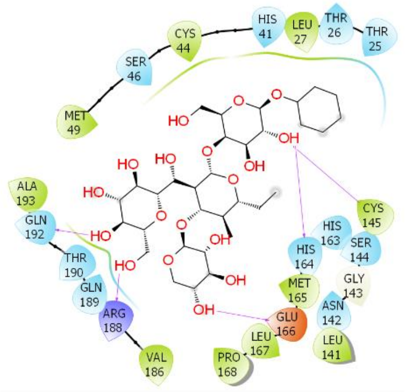
**14**	**T82_22_8_32**	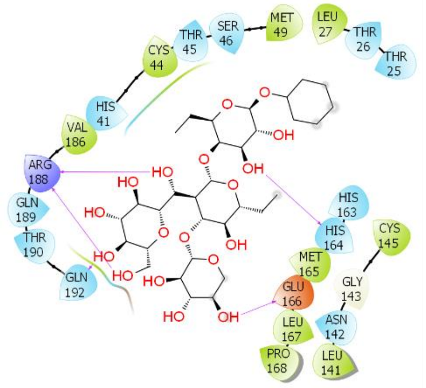
**15**	**T82_22_8_28**	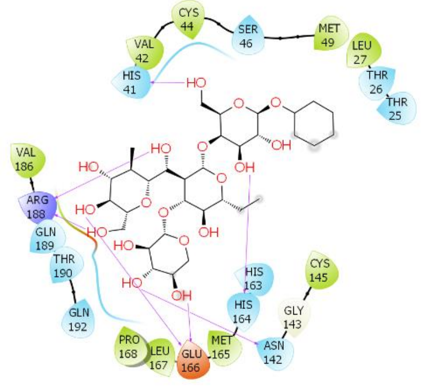
**16**	**T82_22_8_38**	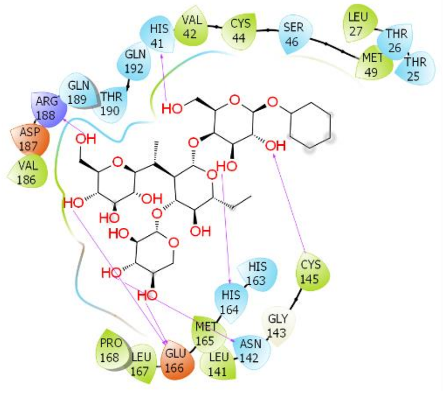

**17**	**T82_22_8_30**	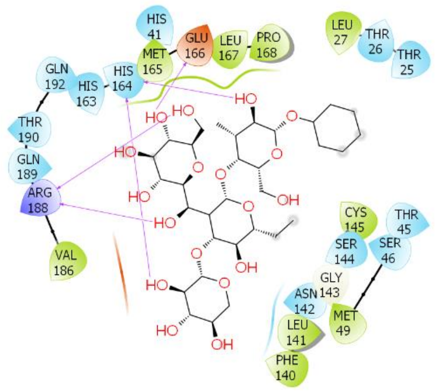
**18**	**T82_22_8_22**	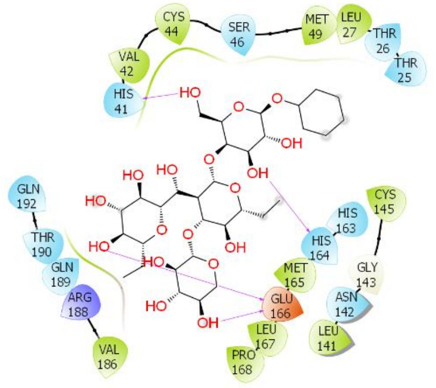

Table S7. MD-refined Structures of SARS-CoV-2 Mpro + Modified Compounds, which were suggested by DeepFrag estimations.

N⁰	Compound	MD-refined structure
**1**	**T117_4**	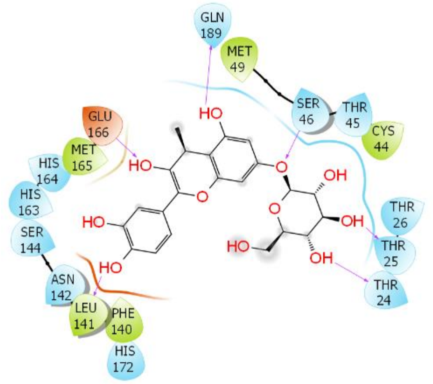
**2**	**T117_16**	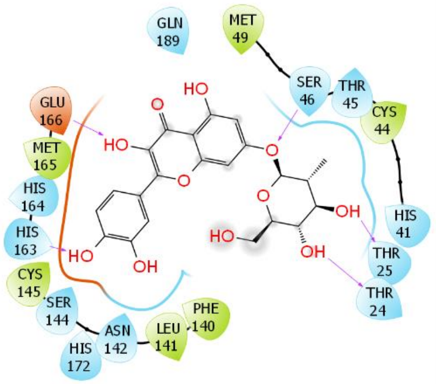
**3**	**T117_18**	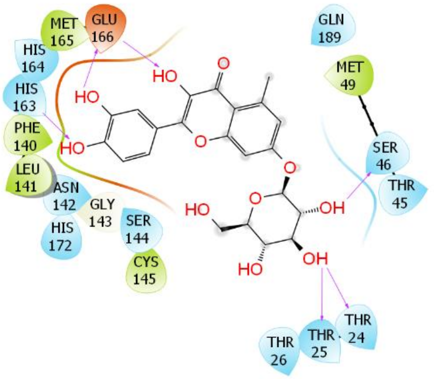
**4**	**T117_20**	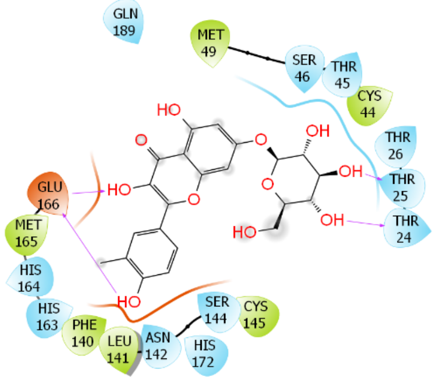

**5**	**T117_22**	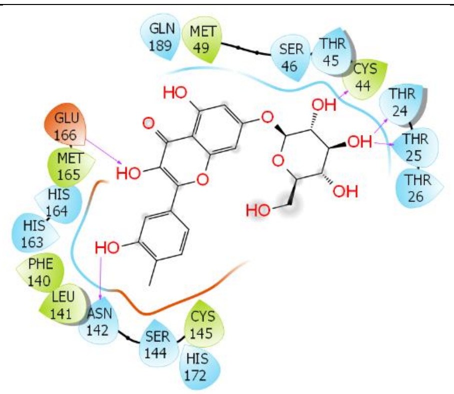
**6**	**T117_24**	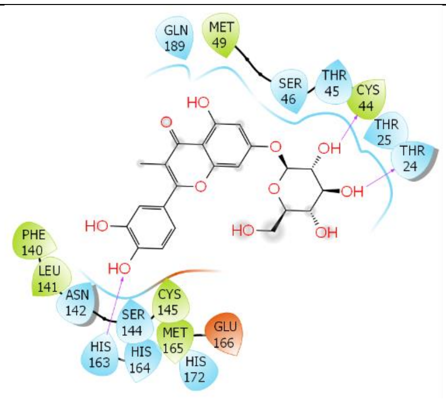

Table S8. The calculated results of 62 modified compounds to SARS-CoV-2 Mpro using DL and FPL calculations.

N⁰	Compound	F_{Max}	W	$\Delta G_{\mathrm{FPL}}^{\mathrm{Pre}}$ [a]	IC_{50}^{Pre} range [b]
1	**T82_22**	870.8 ± 61.6	111.6 ± 6.7	-11.76	Nanomolar
2	**T82_32**	857.9 ± 41.9	108.9 ± 3.4	-11.61	Nanomolar
3	**T82_24**	834.0 ± 19.1	103.0 ± 2.9	-11.28	High-nanomolar
4	**T82_34**	796.2 ± 48.3	97.0 ± 4.9	-10.94	High-nanomolar
5	**T82_cut**	748.3 ± 48.4	96.3 ± 5.2	-10.9	High-nanomolar
6	**T82_38**	820.2 ± 38.2	95.9 ± 5.2	-10.88	High-nanomolar
7	**T82_10**	797.7 ± 22.1	95.0 ± 3.9	-10.83	High-nanomolar
8	**T82_14**	762.9 ± 21.5	94.0 ± 3.3	-10.78	High-nanomolar
9	**T82_12**	772.3 ± 38.6	90.2 ± 5.1	-10.56	High-nanomolar
10	**T17_24**	713.2 ± 49.9	81.2 ± 5.5	-10.06	High-nanomolar
11	**T17_2**	593.1 ± 25.9	72.2 ± 2.9	-9.56	Sub-micromolar
12	**T17_cut**	588.1 ± 49.3	70.7 ± 6.5	-9.47	Sub-micromolar
13	**T17_22**	625.5 ± 34.1	67.7 ± 4.6	-9.30	Sub-micromolar
14	**T17_30**	540.6 ± 28.8	65.8 ± 4.0	-9.19	Sub-micromolar
15	**T17_8**	557.4 ± 42.5	61.3 ± 4.7	-8.95	Sub-micromolar
16	**T17_18**	536.4 ± 30.0	61.0 ± 5.5	-8.93	Sub-micromolar
17	**T17_4**	495.8 ± 26.7	59.5 ± 3.2	-8.84	Sub-micromolar
18	**T17_28**	547.5 ± 30.6	59.3 ± 5.5	-8.83	Sub-micromolar
19	**T17_10**	460.1 ± 35.5	46.7 ± 3.9	-8.13	Micromolar
20	**T56_2**	705.2 ± 18.9	87.9 ± 2.6	-10.43	High-nanomolar
21	**T56_18**	717.4 ± 51.6	81.8 ± 5.3	-10.09	High-nanomolar
22	**T56_8**	655.1 ± 22.9	79.7 ± 3.4	-9.98	High-nanomolar
23	**T56_26**	713.9 ± 39.0	79.0 ± 3.8	-9.94	Sub-micromolar
24	**T56_14**	720.0 ± 26.1	76.5 ± 3.5	-9.80	Sub-micromolar
25	**T56_24**	665.2 ± 23.5	74.6 ± 2.5	-9.69	Sub-micromolar
26	**T56_4**	619.5 ± 23.5	74.3 ± 3.7	-9.68	Sub-micromolar
27	**T56_16**	665.3 ± 29.5	74.2 ± 2.5	-9.67	Sub-micromolar
28	**T56_22**	583.4 ± 30.0	60.7 ± 3.3	-8.91	Sub-micromolar
29	**T82_22_16**	953.0 ± 54.0	121.6 ± 6.1	-12.32	Nanomolar
30	**T82_22_8**	940.4 ± 44.8	120.8 ± 2.7	-12.28	Nanomolar
31	**T82_22_30**	930.1 ± 39.7	112.4 ± 5.8	-11.81	Nanomolar
32	**T82_22_10**	881.9 ± 25.3	108.6 ± 3.9	-11.59	Nanomolar
33	**T82_22_40**	919.0 ± 47.6	108.5 ± 5.8	-11.59	Nanomolar
34	**T82_22_12**	818.3 ± 30.5	107.8 ± 3.6	-11.55	Nanomolar
35	**T82_22_14**	835.6 ± 50.8	105.9 ± 5.2	-11.44	Nanomolar
36	**T82_22_24**	880.4 ± 41.2	105.5 ± 2.9	-11.42	Nanomolar
37	**T82_22_36**	797.9 ± 53.7	98.9 ± 8.2	-11.05	High-nanomolar
38	**T82_22_32**	752.6 ± 30.8	93.9 ± 3.2	-10.77	High-nanomolar
39	**T82_22_16_40**	888.7 ± 39.8	109.7 ± 5.4	-11.65	Nanomolar
40	**T82_22_16_38**	856.3 ± 33.1	106.9 ± 4.0	-11.50	Nanomolar
41	**T82_22_16_18**	856.3 ± 50.3	106.8 ± 6.6	-11.49	Nanomolar
42	**T82_22_16_10**	855.6 ± 58.7	105.2 ± 6.8	-11.40	Nanomolar
43	**T82_22_16_24**	836.1 ± 17.6	103.3 ± 2.1	-11.30	High-nanomolar
44	**T82_22_16_32**	828.1 ± 39.9	98.3 ± 6.5	-11.02	High-nanomolar
45	**T82_22_16_22**	794.9 ± 30.6	95.2 ± 4.6	-10.84	High-nanomolar
46	**T82_22_16_28**	749.4 ± 34.8	94.6 ± 4.3	-10.81	High-nanomolar
47	**T82_22_16_30**	672.4 ± 25.0	82.2 ± 2.6	-10.12	High-nanomolar
48	**T82_22_8_14**	931.1 ± 28.6	117.0 ± 5.2	-12.06	Nanomolar
49	**T82_22_8_24**	860.1 ± 45.3	107.6 ± 4.8	-11.54	Nanomolar
50	**T82_22_8_12**	883.2 ± 45.7	100.1 ± 6.0	-11.12	High-nanomolar
51	**T82_22_8_8**	763.3 ± 30.2	99.6 ± 4.2	-11.09	High-nanomolar
52	**T82_22_8_32**	804.1 ± 32.2	99.1 ± 5.0	-11.06	High-nanomolar
53	**T82_22_8_28**	750.7 ± 26.0	91.1 ± 4.6	-10.61	High-nanomolar
54	**T82_22_8_38**	769.5 ± 57.9	90.7 ± 6.1	-10.59	High-nanomolar
55	**T82_22_8_30**	698.6 ± 30.6	85.8 ± 4.4	-10.32	High-nanomolar
56	**T82_22_8_22**	737.2 ± 22.2	85.5 ± 4.5	-10.30	High-nanomolar
57	**T117_16**	630.3 ± 41.6	72.0 ± 4.9	-9.54	Sub-micromolar
58	**T117_18**	610.2 ± 25.1	65.9 ± 3.1	-9.20	Sub-micromolar
59	**T117_20**	621.2 ± 49.1	61.3 ± 4.7	-8.94	Sub-micromolar
60	**T117_4**	541.1 ± 17.2	60.7 ± 3.0	-8.91	Sub-micromolar
61	**T117_22**	582.7 ± 31.5	56.0 ± 2.2	-8.64	Sub-micromolar
62	**T117_24**	627.9 ± 26.2	55.3 ± 2.4	-8.61	Sub-micromolar

[a]The predicted binding free energy $\Delta G_{\mathrm{FPL}}^{\mathrm{Pre}} = -0.056 \times W - 5.512$.[1] [b]The predicted IC_{50}^{Pre} was calculated via formula $IC_{50}^{\mathrm{Pre}} = e^{(\Delta G_{\mathrm{FPL}}^{\mathrm{Pre}}/RT)}$ using hypothesis that IC_{50} equals to inhibition constant k_{i}. The calculated error is the standard error of the average (SE). The unit of force and energy in pN and kcal mol^{-1}, respectively.

Table S9. The permeability/solubility of the top-lead compounds in Table 1, which formed form sub-micromolar to nanomolar affinities.

N⁰	Code	Pubchem ID	Name	LogP[a]	IC_{50}^{Pre} range[b]
1	**T82**	28523	Tomatine	-0.7	Nanomolar
2	**T17**	159331	Thevetine	-1.6	High-nanomolar
3	**T56**	10175330	Tribuloside	2.5	High-nanomolar
4	**T117**	5282160	Quercimeritrin	0.4	Sub-micromolar
5	**T25**	31310	Scillaren	-0.3	Sub-micromolar
6	**T61**	73568	Corilagin	0.1	Sub-micromolar
7	**T44**	6325292	Gomphrenin III	0.9	Sub-micromolar
8	**T26**	222154	Proscillaridin	1.9	Sub-micromolar
9	**T33**	185586	Melianotriol	5.0	Sub-micromolar
10	**T52**	441840	Adynerin	3.1	Sub-micromolar
11	**T24**	5317157	Equisetrin	-1.4	Sub-micromolar
12	**T3**	5281627	Hinokiflavone	4.4	Sub-micromolar
13	**T202**	441295	Ginkgolide C	-1.4	Sub-micromolar
14	**T55**	5316647	Cynarine	1.5	Sub-micromolar
15	**T126**	5280805	Rutin	-1.3	Sub-micromolar
16	**T34**	185617	Scutellarin	0.8	Sub-micromolar
17	**T19**	10028469	Melianodiol	4.7	Sub-micromolar

[a]logP of the compounds was obtained from PubChem database.

Table S11. The PreADMET results of 62 designed inhibitors.

N⁰	Compound	HIA (%)	LogP	Carcino (Rat)
1	**T82_22**	0.00	-5.66	negative
2	**T82_32**	0.39	-3.94	negative
3	**T82_24**	0.00	-5.52	negative
4	**T82_34**	0.39	-3.92	negative
5	**T82_cut**	0.03	-4.95	negative
6	**T82_38**	0.39	-3.94	negative
7	**T82_10**	0.06	-5.05	negative
8	**T82_14**	0.00	-5.25	negative
9	**T82_12**	0.00	-5.66	negative
10	**T17_24**	8.07	-1.33	negative
11	**T17_2**	5.10	-1.84	negative
12	**T17_cut**	3.51	-2.35	negative
13	**T17_22**	8.07	-1.32	negative
14	**T17_30**	5.10	-1.70	negative
15	**T17_8**	2.46	-2.78	negative
16	**T17_18**	8.07	-1.33	negative
17	**T17_4**	5.10	-2.09	negative
18	**T17_28**	8.07	-1.33	negative
19	**T17_10**	2.46	-2.91	negative
20	**T56_2**	39.28	1.09	negative
21	**T56_18**	39.30	1.23	negative
22	**T56_8**	64.13	2.55	negative
23	**T56_26**	73.73	2.69	negative
24	**T56_14**	74.03	2.57	negative
25	**T56_24**	73.73	2.69	negative
26	**T56_4**	61.18	2.11	negative
27	**T56_16**	73.73	2.68	negative
28	**T56_22**	74.03	2.57	negative
29	**T82_22_16**	0.00	-5.76	negative
30	**T82_22_8**	0.17	-4.23	negative
31	**T82_22_30**	0.17	-4.63	negative
32	**T82_22_10**	0.17	-4.63	negative
33	**T82_22_40**	0.17	-4.44	negative
34	**T82_22_12**	0.00	-6.35	negative
35	**T82_22_14**	0.00	-5.95	negative
36	**T82_22_24**	0.17	-4.23	negative
37	**T82_22_36**	0.17	-4.64	negative
38	**T82_22_32**	0.17	-4.63	negative
39	**T82_22_16_40**	0.22	-4.17	negative
40	**T82_22_16_38**	0.22	-4.54	negative
41	**T82_22_16_18**	0.22	-4.74	negative
42	**T82_22_16_10**	0.22	-4.73	negative
43	**T82_22_16_24**	0.22	-4.73	negative
44	**T82_22_16_32**	0.22	-4.32	negative
45	**T82_22_16_22**	0.22	-4.32	negative
46	**T82_22_16_28**	0.22	-4.73	negative
47	**T82_22_16_30**	0.22	-4.73	negative
48	**T82_22_8_14**	0.22	-4.32	negative
49	**T82_22_8_24**	0.72	-3.20	negative
50	**T82_22_8_12**	0.00	-4.52	negative
51	**T82_22_8_8**	0.72	-3.20	negative
52	**T82_22_8_32**	0.72	-2.80	negative
53	**T82_22_8_28**	0.72	-3.20	negative
54	**T82_22_8_38**	0.72	-3.01	negative
55	**T82_22_8_30**	0.00	-5.66	negative
56	**T82_22_8_22**	0.39	-3.94	negative
57	**T117_4**	18.77	0.35	negative
58	**T117_16**	27.45	0.72	negative
59	**T117_18**	27.72	0.35	negative
60	**T117_20**	27.73	0.35	negative
61	**T117_22**	27.72	0.35	negative
62	**T117_24**	27.72	0.61	negative

Table S11. The toxicity results of 41 natural compounds, which were reported in Table 1.

N^0	Compound	Carcino (Rat)
1	**T82**	negative
2	**T17**	negative
3	**T56**	negative
4	**T117**	negative
5	**T25**	negative
6	**T61**	negative
7	**T44**	negative
8	**T26**	negative
9	**T33**	negative
10	**T52**	negative
11	**T24**	negative
12	**T3**	negative
13	**T202**	negative
14	**T55**	negative
15	**T126**	negative
16	**T34**	negative
17	**T19**	negative
18	**T13**	negative
19	**T121**	negative
20	**T27**	negative
21	**T115**	negative
22	**T182**	negative
23	**T14**	negative
24	**W7**	negative
25	**T179**	negative
26	**T58**	negative
27	**T65**	negative
28	**T35**	negative
29	**T23**	negative
30	**T107**	negative
31	**T20**	negative
32	**T7**	negative
33	**T50**	negative
34	**T180**	negative
35	**T30**	negative
36	**T4**	negative
37	**T8**	negative
38	**T102**	negative
39	**T1**	negative
40	**T82**	negative
41	**T17**	negative

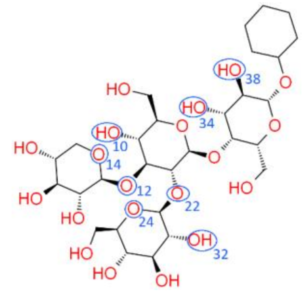

T82_cut

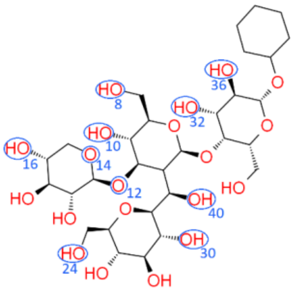

T82_22

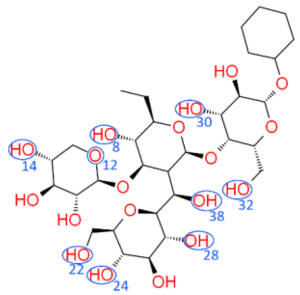

T82_22_8

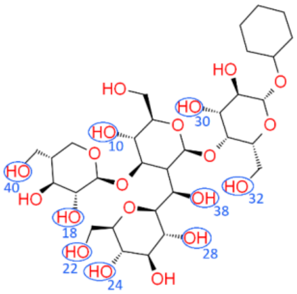

T82_22_16

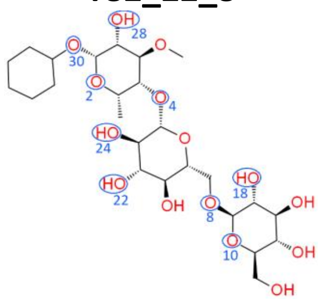

T17_cut

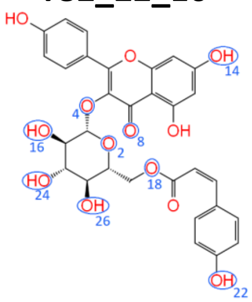

T56

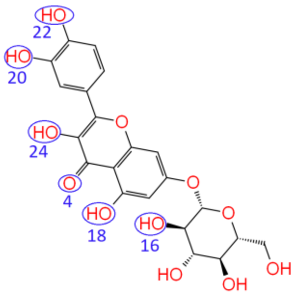

T117

Figure S1. The modified positions of the studied compounds, in which the numbers correspond to the atomic index.

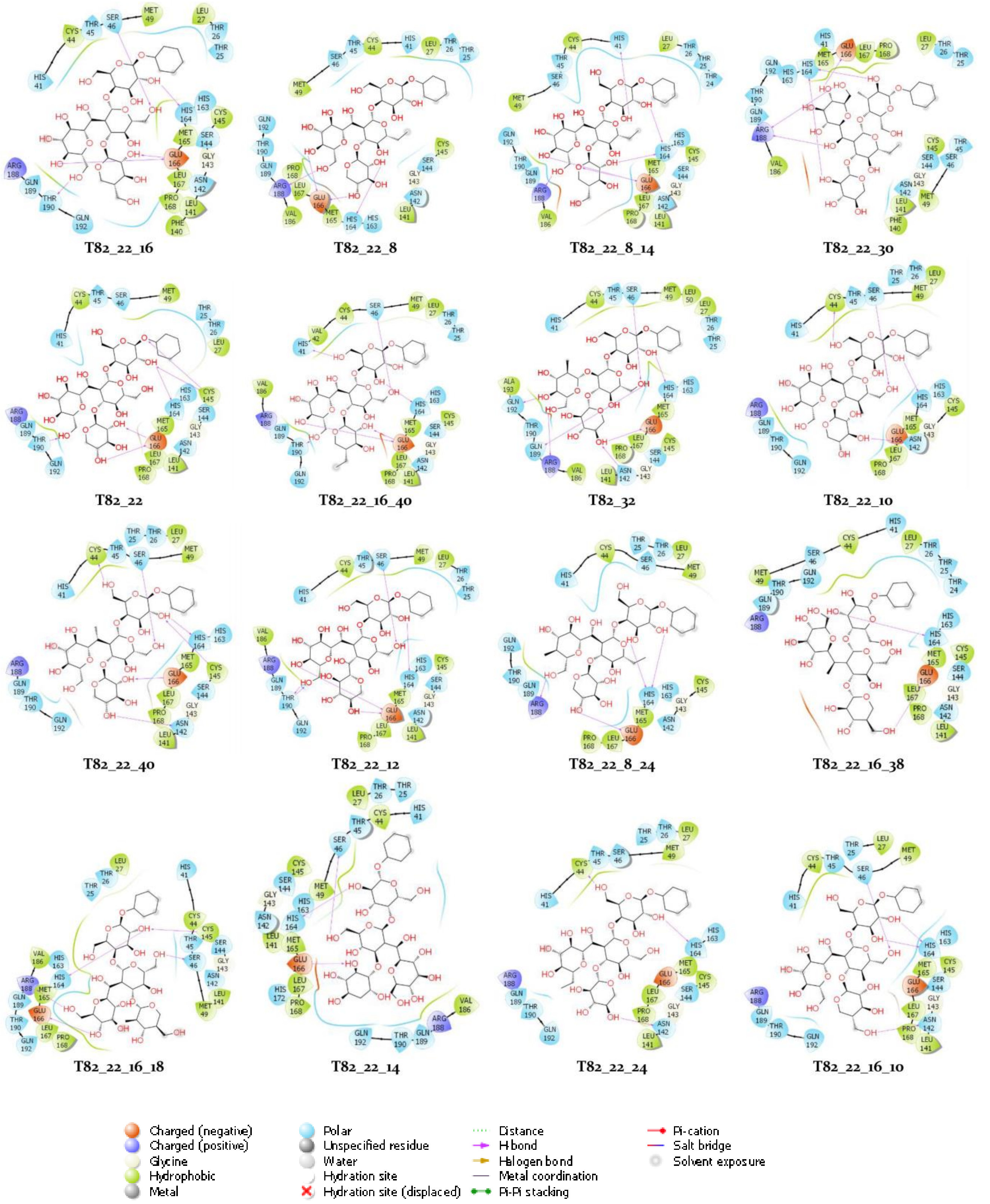

Figure S2. 2D interaction diagram of top-lead designed inhibitors to SARS-CoV-2 Mpro. The MD-refined structure of the complexes was obtained using the clustering method with a cutoff of 0.12 nm.

Reference

1. Pham, M. Q.; Vu, K. B.; Han Pham, T. N.; Thuy Huong, L. T.; Tran, L. H.; Tung, N. T.; Vu, V. V.; Nguyen, T. H.; Ngo, S. T., Rapid prediction of possible inhibitors for SARS-CoV-2 main protease using docking and FPL simulations. *RSC Adv* **2020,** *10* (53), 31991-31996.
